# A Biotic Game Design Project for Integrated Life Science and Engineering Education

**DOI:** 10.1371/journal.pbio.1002110

**Published:** 2015-03-25

**Authors:** Nate J. Cira, Alice M. Chung, Aleksandra K. Denisin, Stefano Rensi, Gabriel N. Sanchez, Stephen R. Quake, Ingmar H. Riedel-Kruse

**Affiliations:** Department of Bioengineering, Stanford University, Stanford, California, United States of America

## Abstract

Engaging, hands-on design experiences are key for formal and informal Science, Technology, Engineering, and Mathematics (STEM) education. Robotic and video game design challenges have been particularly effective in stimulating student interest, but equivalent experiences for the life sciences are not as developed. Here we present the concept of a "biotic game design project" to motivate student learning at the interface of life sciences and device engineering (as part of a cornerstone bioengineering devices course). We provide all course material and also present efforts in adapting the project's complexity to serve other time frames, age groups, learning focuses, and budgets. Students self-reported that they found the biotic game project fun and motivating, resulting in increased effort. Hence this type of design project could generate excitement and educational impact similar to robotics and video games.

## Abstract 2

Can games teach life sciences device design? This article in our education series describes a "biotic game design course" to motivate student learning at the interface of life sciences and device engineering.

*This Education article is part of the Education Series*.

Hands-on robotic and video game design projects and competitions are widespread and have proven particularly effective at sparking interest and teaching K–12 and college students in mechatronics, computer science, and Science, Technology, Engineering, and Mathematics (STEM). Furthermore, these projects foster teamwork, self-learning, design, and presentation skills [1,2]. Such playful and interactive media that provide fun, creative, open-ended learning experiences for all ages are arguably underdeveloped in the life sciences. Most hands-on education occurs in traditionally structured laboratory courses with a few exceptions like the International Genetically Engineered Machine (iGEM) competition [3]. Furthermore, there is an increasing need to bring the traditional engineering and life science disciplines together. In order to fill these gaps, we present the concept of a biotic game design project to foster student development in a broad set of engineering and life science skills in an integrated manner (Fig. 1). Though we primarily discuss our specific implementation as a cornerstone project-based class [4], alternative implementations are possible to motivate a variety of learning goals under various constraints such as student age and cost (see supplements for all course material).

**Fig 1:**
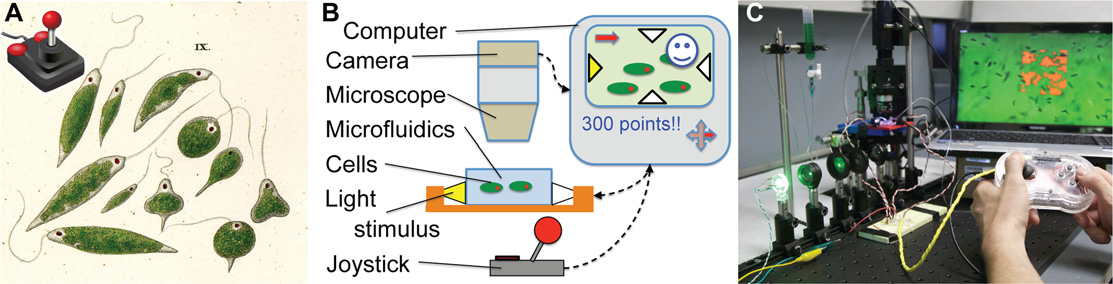
We developed a bioengineering devices course that employed biotic game design as a motivating project scheme. **A:** Biotic games enable human players to interact with cells. **B:** Conceptual overview of a biotic game setup. **C:** Students built and played biotic games. Image credits: **A** C64 joystick by Speed-link, 1984 (http://commons.wikimedia.org/wiki/File:Joystick_black_red_petri_01.svg); *Euglena viridis* by C. G. Ehrenberg, 1838; **C** Photo, N. J. C.

Biotic games are games that operate on biological processes (Fig. 1) [5]. The biotic games we present here involve the single-celled phototactic eukaryote, *Euglena gracilis*. These microscopic organisms are housed in a microfluidic chip and are displayed in a magnified image on a video screen. Players interact with these cells by modulating the intensity and direction of light perpendicular to the microfluidic chip via a joystick, thereby influencing the cells’ phototactic motion. Software tracks the position of individual euglena with respect to virtual objects overlaid on the screen, creating myriad opportunities for creative game design and play. For example, in a simple game, points might be scored when a cell hits a virtual box (see S1 Video).

The biotic game design project we developed was intended to motivate all the broad categories of theoretical and hands-on skills for creating any integrated instrument intended to house and to interface with biological materials, i.e., optics, electronics, sensing, actuation, microfluidics, fabrication, image processing, programming, and creative design. We termed the synthesis of these skills “biotics” in analogy to mechatronics. Our intended audience for this course was bioengineering undergraduate students at Stanford University who already had some programming experience but little to no experience in device design, fabrication, and integration. We also incorporated bioethics into the curriculum to emphasize the social responsibility of every engineer and demonstrate the potential for the biotic game project to motivate multiple fields. The course we taught spanned ten weeks, divided roughly equally into a set of technical units and the biotic game project, with two 4-hour lab sections and a single 1.5-hour lecture each week. For details and all course documents, please refer to the supplemental material.

The technical section of the course focused on developing hands-on skills and theoretical understanding related to devices in a conventionally structured laboratory setting. We introduced students to fundamental electronics concepts and components such as voltage, current, resistors, capacitors, LEDs, filters, operational amplifiers, motors, microcontrollers (Arduino Uno), and breadboards. We followed a similar traditional approach in introducing optics, presenting the thin lens equation, ray tracing, conjugate planes, basic optical system design, and Köhler illumination. We covered additional topics in less detail: MATLAB programming, particle tracking, computer-aided design (CAD), fabrication, and microfluidics (learning objectives are provided at the beginning of each unit in the supplemental material).

During the project-based section, students built their own biotic games. We left specific choices of implementation, architecture, and design to the students to encourage creativity and exploration but required students to revisit the technical skills they learned in the first section by integrating some specific requirements into their games (Fig. 2). Students built a bright field microscope with Köhler illumination and projected their images onto a webcam (optics). Glass and polydimethylsiloxane (PDMS) components comprised the microfluidic chip (microfluidics) and housed the euglena (microbiology). The holder for the chip and euglena-steering LEDs was designed in Solidworks (CAD) and 3-D printed (fabrication). The students constructed a polycarbonate housing for the game controller using a band saw and drill press (fabrication). The students revisited electronic breadboarding and soldering when creating the electronic circuits to communicate between the LEDs, joystick, microcontroller, and computer. Finally, they used MATLAB to program the microcontroller, implement real time image recognition, and provide the user interface for the game experience (image processing and programming).

**Fig 2:**
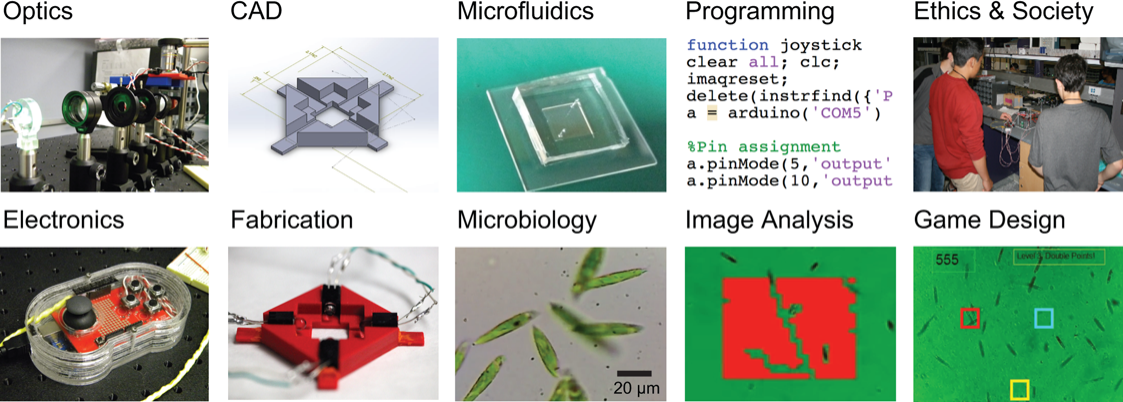
Biotic game-based courses encourage students to integrate a versatile set of relevant STEM topics. Image credits: Taken by N. J. C. (credit for the work and artifacts to the students who took the course).

We challenged students to consider the ethical implications [6] of manipulating life in a game context before building their projects. Although phototaxis experiments with euglena are commonplace in education, and have hitherto raised no ethical concerns, the equivalent manipulation in the form of a game warrants its own ethical analysis as provided by Harvey et al. [7]. The students read and discussed this paper, then wrote a 200-word essay on whether they found it permissible or not to make and play biotic games. Students had the choice to switch to a nongame project of equivalent complexity. All students found euglena-based games permissible, pointing out that “they are nonsentient and cannot feel pain,” followed by a diverse range of considerations such as “the euglena are still free to act as they please,” “there needs to be an educational intention,” or “a pet…provides a way…to work on responsibility and caring.” Based on further student-initiated discussions that spontaneously emerged throughout the course, we believe that biotic games are effective in providing a stimulating, student-relevant, in-class context for bioethics.

We motivated the game design project to the students as having educational potential at two levels, i.e., learning by building and learning by playing; we lectured them about the needs and opportunities for new approaches to K–12 STEM education [8,9]. The students were then asked to consider building a game that had educational value for the player. Educational value has many aspects, which was reflected in students’ statements regarding their intended educational outcomes for their games on their course project websites. These ranged from more factual learning objectives (“learn about…” “…inner working,” “…structural detail,” “… light responses,” “…euglena behavior”) to objectives affecting attitude (“spark interest,” “generate fascination,” “encourage to explore,” “respect for life”). We also had a game designer give a guest lecture to the students. For pragmatic reasons, we requested the students keep games very simple (ideally having just a single in-game objective) and cap game duration at one minute. Before, during, and after their projects, students received feedback from instructors as well as from their peers on their games from technical and user perspectives.

The games that the students ultimately produced were diverse and creative (Fig. 2 and S1 Video), including single and multiplayer scenarios, games where euglena hit virtual targets, and games where euglena pushed virtual objects. Games that involved pushing objects across the screen (relying on collective motion of many organisms) were generally more consistent at correlating player strategy to scored points than those that involved hitting target objects. The quality and robustness of these integrated projects naturally varied, and individual groups placed more or less emphasis on different aspects based on personal preferences and learning goals (for example, fabricating a more elaborate housing for the game controller versus programming more complex game mechanics). A key point was that the students did not rely on prepared materials or platforms to develop their games but rather had to design, build, and test their game setups from scratch, thereby revisiting and deepening the primary learning goals of the course with some freedom to follow their own learning aspirations (Fig. 2). The final project deliverables were a two-minute project demonstration video, a website describing the elements of the project, and a game that all instructors and students played on the final day (Fig. 1B), which led to lots of laughter as well as in-depth discussions on technical details.

Many students self-reported that they enjoyed the project and that it led to increased motivation and effort during the course. In response to the question “Do you think you were motivated to try harder or had more fun (and thereby learned more) during your final project because you were making a game (rather than just building a technical instrument, for example)? If so—please give some examples:” 15 out of 17 students responded “Very/definitely” on a five point scale. As examples, students listed: “wanted to make the best game,” “want to make it clever and cool in the eyes of classmates who are play testing,” “motivated during final push,” “willing to put in more time,” “was fun”/”made it fun,” “create a game that actually works,” “reinforced what was learned before,” and “provided room for creativity.” These comments reflect the overall excitement we saw for the biotic game project. While these responses do not constitute rigorous proof regarding course effectiveness (which will require more detailed and controlled assessments in the future), we consider this course a success based on our teaching experiences.

45 students have now taken this class over the past three years, with 18 students in our most recent offering. We used each year to iterate and improve our implementation. For example, we changed the organism and stimulus from *Paramecia* galvanotaxis [5] to *Euglena* phototaxis, which gave more reliable long-term responses. We also added a simple microfluidics unit enabling students to build more robust organism housing chambers. We changed the microscope structure from LEGO to Thorlabs parts (essentially trading the emphasis on 3-D structural design, flexibility, and cost for a more in-depth focus on high-end optics and their alignment). Finally, we explicitly asked the students to design and fabricate a housing for the game controller to better incorporate fabrication skills like using a band saw and tapping screw threads. So far, we primarily used MATLAB as the programming component given its widespread use in education and research and the available Arduino interface. However, MATLAB is not particularly well-suited to support game design and is also not free, making translation into lower resource settings challenging. For the future, we are considering moving to smartphone-based control (such as Android) given that these mobile environments are very flexible and increasingly used for control of scientific and consumer instruments and are becoming more widespread in education. We also see the opportunity to better emphasize and teach the approach of iterative design; for example, by letting students prototype and test their game ideas on paper [10] and simple programming environments like Scratch [11] first, before attempting the full implementation. It would likely also be very rewarding for the students to be able to take their project home at the end of the course. In summary, many different course design decisions can be made based on specific intended educational outcomes. Not all of these can be fit into one course at the same time, and clear decisions should be made on how to balance covering a breadth of topics with depth on a selected few.

As a preliminary test of another age range, time frame, and budget, we taught a greatly simplified 3-hour workshop where high school and middle school students assembled a low-cost microscope and microfluidics chamber, attached it to a smartphone, and stimulated euglena using a preprogrammed Arduino-based controller (see supplements). We had no game interface implemented yet on the phone, but the students could observe the euglena responses to the light stimuli. All students were able to complete the project and take their microscopes home. Over half of our undergraduate student teams also volunteered to present their game projects for this outreach event which took place multiple weeks after their class had ended. This separate experience suggests that the biotic game concept holds promise for reaching a wider age range in a shortened timespan and at a greatly reduced budget, and that completed games can be used in outreach activities. We are currently developing a kit modeled after this unit.

In conclusion, we consider biotic games promising in motivating integrated, hands-on learning at the interface of life science and engineering. Our efforts so far indicate that this concept could be adapted to various age groups and learning goals with the potential for wider future impacts on education. We draw upon the analogy to robotics, where microcontrollers went from initially unfathomable as an educational tool to the vision of Papert and collaborators and their use of programmable robotics with children [12], eventually leading to multiple commercial realizations (LEGO mindstorm, Arduino, etc.), a large public following, and a major role in education both in the classroom and through competitions such as First Robotics [1]. We also see additional potential for integrating more creative and artistic aspects into STEM, i.e., leading to generalized Science, Technology, Engineering, Arts, and Mathematics (STEAM) disciplines [13]. We invite others to join us in these endeavors—all instructional materials are available in the appendix for further adaptations and educational use.

## Supporting Information

S1 Text Guide—Read first. This overview document explains the contents of the supplemental material. It is to be used as a guide.
(DOCX)

S2 Text pdf format of S1 Text. (PDF)

S3 Text Syllabus. This document provides the schedule, high-level objectives, and logistical information for the course.
(DOCX)

S4 Text pdf format of S3 Text. (PDF)

S5 Text Module 01 measurement and error analysis HW LS. This module introduces students to fundamentals of measurement and error analysis by measuring the height of drawn pencil lines using two different methods. Students begin learning basic electronics skills.
(DOCX)

S6 Text pdf format of S5 Text. (PDF)

S7 Text Module 02 electronics HW LS. This module provides homework and lab instructions for the first electronics unit involving voltage, current, and resistance.
(DOCX)

S8 Text pdf format of S7 Text. (PDF)

S9 Text Module 03 electronics HW LS. This module provides homework and lab instructions for the second electronics unit involving filters.
(DOCX)

S10 Text pdf format of S9 Text. (PDF)

S11 Text Module 04 electronics HW LS. This module provides homework and lab instructions for the third electronics unit involving operational amplifiers.
(DOCX)

S12 Text pdf format of S11 Text. (PDF)

S13 Text Module 05 electronics HW LS. This module provides homework and lab instructions for the fourth electronics unit involving Arduino microcontrollers. Students make a simple game.
(DOCX)

S14 Text pdf format of S13 Text. (PDF)

S15 Text Module 06 electronics HW LS. This module provides homework and lab instructions for the fifth electronics unit which involves pulse width modulation, motors, and transistors.
(DOCX)

S16 Text pdf format of S15 Text. (PDF)

S17 Text Module 07 to 11 optics HW. This document provides all homework problems for the optics section of the course.
(DOCX)

S18 Text pdf format of S17 Text. (PDF)

S19 Text Module 07 to 11 optics LS. This document provides all laboratory instructions for the optics section of the course.
(DOCX)

S20 Text pdf format of S19 Text. (PDF)

S21 Text Module 12 microfluidics. This module provides homework and lab instructions for the microfluidics section of the course. Students learn basic fabrication, terminology, and physics for small devices.
(DOCX)

S22 Text pdf format of S21 Text. (PDF)

S23 Text Module 13 to 20 final project. This document provides requirements, notes, and starter modules on various components of the final biotic game project.
(DOCX)

S24 Text pdf format of S23 Text. (PDF)

S25 Text Alternative biotic game project implementation. This document goes over the time, resources, and instructions needed to run a short version of the biotic game project.
(DOCX)

S26 Text pdf format of S25 Text. (PDF)

S1 Video Project video. This video shows examples of the student biotic game projects.
(MOV)

S1 Code Game-run code. This document provides MATLAB code for an example game. Note that it is provided as is and may depend on specific hardware, software, or toolboxes.
(ZIP)

## Abbreviations

CAD: computer-aided design
iGEM: International Genetically Engineered Machine
PDMS: polydimethylsiloxane
STEAM: Science, Technology, Engineering, Arts, and Mathematics
STEM: Science, Technology, Engineering, and Mathematics
