# Supplementary material for: A Biotic Game Design Project for Integrated Life Science and Engineering Education
Source: PLoS Biol. 2015 Mar 25;13(3):e1002110. doi: 10.1371/journal.pbio.1002110 (PMC4373802; doi:10.1371/journal.pbio.1002110)
Supplement: S2 Text — (PDF) [file pbio.1002110.s002.pdf]

Course materials for:

A biotic game design project for integrated life science and engineering education

Here we have included all course instructions needed to setup and teach a course on biotic games. The material should enable others to teach a similar course, or just focus on the game design portion (or even a subset of that). These materials are presented 'as taught' in an ever evolving course (we now have taught it 3 times); we encourage future updates and modifications. It is recommended that instructors perform each laboratory once before students and tune the level of presentation to fit the background of the students and the resources available. We found having students work in pairs facilitated discussion and completion of material.

The supplemental material is organized into the following sections:

I. Logistics (incl. example syllabus with course schedule)
II. Measurement
III. Electronics
IV. Optics
V. Microfluidics
VI. Biotic Game Project

<u>If you wish to:</u>

Teach the entire course:

- Start with the syllabus and read through all the course materials

Teach only the biotic game project portion of the course:

- Start with the documents in section VI, The Biotic Game Project section.

Teach a highly abbreviated version of the biotic game project portion of the course:

- Start with the document in section VI, "Alternative biotic game project implementation"

Additional Information:

Measurement, electronics, optics, and microfluidics sections have modular labs designed for all students to start and finish within one lab period. Each unit of the these sections is composed of the following elements:

- HW (Home work): includes reading and simple comprehension problems to be completed before lab
- LS (Lab instructions): includes learning objectives, step by step instructions for labs, prompted discussion/report questions, and materials lists for each lab

During the biotic game section students create a project which strengthens and builds on these fundamentals**.** We have included several documents to assist readers with this portion of the course. 1) The course instructions for the project. During the project phase students worked largely independently and, from our experience, often worked on different aspects of their projects, therefore we have not included day-by-day instructions for this section, rather: a lecture on the project, an overview of the game objectives, and several modules which address skills needed for executing different aspects of the game. 2) Example code from a student game, which may save time in learning how to integrate the various components, particularly reading the webcam into MATLAB. Please note that this code may be specific to the various hardware, software, and toolboxes used. 3) Our outline and parts list for a simplified, lower cost, reduced time and complexity version of the course.

The course always ran for 10 weeks. The first two years we meet with the students 2 times a week for 4 hour- lab sessions, which were usually started off with a 30-60 min discussion on necessary concepts (often preceded by some homework reading and exercises). The third year we switched to 1.5 hour lecture once a week, followed by ~3 hour lab periods each week for 10 weeks. Which format is better, i.e., how to best integrated theory and hands-on, is presumably a matter of person style – some research even indicates that having students first toy around in the lab (and partially fail), then bring in the theory, followed again by a lab section might even be more advantageous.

We present these materials for open access and use. We encourage users to, borrow, supplement, and modify as needed – we therefore provide them both as pdf and word documents for convenience.

Supplemental Materials for:

A biotic game design project for integrated life science and engineering education

Type	Title	Size
**Overview**	Guide – READ FIRST (S1,2 Text)	<0.1 Mb, 0.2 Mb,
This overview document explains the contents of the supplemental material. It is to be used as a guide.		
**I. Logistics**	Syllabus (S3,4 Text)	<0.1 Mb, <0.1 Mb
This document provides the schedule, high level objectives, and logistical information for the course.		
**II. Measurement**	Module 01 Measurement and Error Analysis HW LS (S5,6 Text)	<0.1 Mb, <0.1 Mb
This module introduces students to fundamentals of measurement and error analysis by measuring the height of drawn pencil lines using two different methods. Students begin learning basic electronics skills.		
**III. Electronics**	Module 02 Electronics HW LS (S7,8 Text)	<0.2 Mb, <0.2 Mb
This module provides homework and lab instructions for the first electronics unit involving voltage, current, and resistance.		
**III. Electronics**	Module 03 Electronics HW LS (S9,10 Text)	<0.2 Mb, <0.2 Mb
This module provides homework and lab instructions for the second electronics unit involving filters.		
**III. Electronics**	Module 04 Electronics HW LS (S11,12 Text)	<0.2 Mb, <0.2 Mb
This module provides homework and lab instructions for the third electronics unit involving operational amplifiers.		
**III. Electronics**	Module 05 Electronics HW LS (S13,14 Text)	<0.2 Mb, <0.2 Mb
This module provides homework and lab instructions for the fourth electronics unit involving Arduino microcontrollers. Students make a simple game.		
**III. Electronics**	Module 06 Electronics HW LS (S15,16 Text)	<0.2 Mb, <0.2 Mb
This module provides homework and lab instructions for the fifth electronics unit which involves pulse width modulation, motors, and transistors.		
**IV. Optics**	Module 07 to 11 Optics HW (S17,18 Text)	3, 26 Mb
This document provides all homework problems for the optics section of the course.		
**IV. Optics**	Module 07 to 11 Optics LS (S19,20 Text)	2, 7 Mb
This document provides all laboratory instructions for the optics section of the course.		
**V. Microfluidics**	Module 12 Microfluidics (S21,22 Text)	
This module provides homework and lab instructions for the microfluidics section of the course. Students learn basic fabrication, terminology, and physics for small devices.		
**VI. Biotic Game Project**	Module 13 to 20 Final project (S23,24 Text)	8, 9 Mb
This document provides requirements, notes, and starter modules on various components of the final biotic game project.		
**VI. Biotic Game Project**	Alternative biotic game project implementation(S25,26 Text)	1.5, 0.5 Mb
This document goes over the time, resources, and instructions needed to run a short version of the biotic game project.		
**VI. Biotic Game Project**	Project Video (S1 Video)	4.3 Mb
This video shows examples of the student biotic game projects.		
**VI. Biotic Game Project**	Game Run Code(S1 Code)	<0.1 Mb
This document provides MATLAB code for an example game. Note that it is provided as is and may depend on specific hardware/software/toolboxes.		
