# Supplementary material for: A Biotic Game Design Project for Integrated Life Science and Engineering Education
Source: PLoS Biol. 2015 Mar 25;13(3):e1002110. doi: 10.1371/journal.pbio.1002110 (PMC4373802; doi:10.1371/journal.pbio.1002110)
Supplement: S3 Text — This document provides the schedule, high-level objectives, and logistical information for the course. (DOCX) [file pbio.1002110.s003.docx]

# Optics and Devices Lab Course

**General Information**

Textbooks:

Practical Electronics for Inventors, 2^nd^ ed. by Scherz

Fundamentals of Light Microscopy, Spencer

Measurements and their Uncertainties, Hughes and Hase

**Course overview**

This course provides a hands-on introduction to designing, building, and evaluating devices for controlling experiments in the field of bioengineering. This course focuses primarily on the tools and concepts related to optics and electronics but also touches on other valuable techniques such as rapid prototyping, image analysis, and microfluidics. The first part of the course consists of guided modules such as building microscopes and electronic circuits, while the second half of the course is more open and project-based. Students will design and develop their own computer controlled microscope and biotic gaming system.

**Learning objectives of the course (General)**

- Learn to design and build devices to control and measure biology
- Become a scientist: learn how to make precision measurements, design experiments and analyze data.
- Become an engineer: learn creativity, develop key skills, get things to work, and get an introduction to the design process.
- Learn how to document your work in the laboratory.
- Gain experience with instrumentation and tools. Become comfortable in a laboratory setting, make things work with what is at hand, and know what tools are out there.
- Gain experience completing an open-ended team project.
- Become an independent learner / be able to find and utilize resources on the web
- Have fun!

**Learning objectives of the course (Specific)**

- Be able to enter any BIOE (or other) lab and build arbitrary experimental setups/devices/structures across scales ranging from 0.1mm-1m.
- Interface with (micro-)biological systems by controlling light / electric signals / fluids /mechanical motion
- Integrate actuators and sensors into your setups (motors, LEDs, camera, …)
- Interface equipment with the computer
- Write programs (Matlab and Arduino native environment) that permit actuation of devices and recording of data (i.e. automate experiments)
- Be able to properly analyze your data and perform error analysis
- Learn a suite of tools for achieving the above goals (analog electronics, oscilloscopes, CAD, basic prototyping techniques)
- Get an introduction to the above topics, and identify key areas for future study to increase depth

**Course structure**

- 20 modules, 4 hours each + exams week (see table below).
- Monday lectures provide practical instruction for completing labs
- The first 12 modules focus on measurement, electronics, optics, and micro-fluidics.
- The last 8 modules are dedicated to your project and include additional topics such as object tracking, rapid prototyping, microcontrollers, etc.
- The ultimate goal of the project is to develop an integrated setup for tracking and controlling swimming cells inside a chamber via computer and human interaction (a biotic game). Working devices are intended for educational outreach in schools and museums, which is good for STEM education, future NSF fellowship applications, and your own enjoyment.
- Students work in teams of two. Each student will keep their own notebook, and do their own assignments, but teams only have to hand in one final project report.

| **Week** | **Lecture topic** | **Lab Topic (Modules)** | **Key Concepts** |
| --- | --- | --- | --- |
| 1 | Intro, error analysis, basic electronics | Intro | Intro/Safety / Measurement/Error analysis, multimeter |
|  |  | Electronics 1 | Ohms Law, LEDs, multimeter |
| 2 | Electronics continued | Electronics 2 | RC Filters, Function Generator |
|  |  | Electronics 3 | Op Amps, amplifiers, buffers, band-pass filter |
| 3 | No lecture | Electronics 4 | Arduino microcontroller, programing basics, I/O |
|  |  | Electronics 5 | Driving motors with Arduino, mosfetts, PWM |
| 4 | Introduction to optics | Optics 1 | Optics basics, build an infinity space microscope |
|  |  | Optics 2 | Focal lengths of lenses, Kepler & Galilean telescopes |
| 5 | Optics continued | Optics 3 | Numerical Aperture, resolution, Point Spread Function, conjugate planes, Kohler illumination |
|  |  | Optics 4 | Darkfield & oblique illumination |
| 6 | Optics and electronics concepts review | Optics 5 | Using mirrors, imaging lab with biological specimens, recording Brownian motion |
|  |  | Microfluidics | Build flow chamber; observe microorganisms (Euglena) |
| 7 | Event driven programming and image analysis | Project 1 | Project introduction; light microscope; start project webpage |
|  |  | Project 2 | Object tracking and recognition with Matlab |
| 8 | Prototyping / Machining | Project 3 | Build out full control with Arduino |
|  |  | Project 4 | Rapid prototyping, Machining; CAD and 3D printer |
| 9 | Discussion bioethics, game design, outreach | Project 5 | Discuss final project write-up and presentation; build out full system |
|  |  | Project 6 | Free project time |
| 10 | No lecture | Project 7 | Free project time |
|  |  | Project Presentation | Final project presentation |

**Module structure**

The four-hour modules typically start with a brief introduction to the lab exercises for the day. After the lab intro, students are dismissed to the lab.

For each lab module there is a small problem set to test your knowledge of the relevant material. The problem sets are turned in at the start of the lecture, and will be returned to the students to use in the labs when relevant. Notebooks are typically handed to the instructors at the end of the four-hour period for grading and final discussions. Lab time may exceed four hours on a voluntary basis, with instructor approval, especially during the project phase.
