# Supplementary material for: A Biotic Game Design Project for Integrated Life Science and Engineering Education
Source: PLoS Biol. 2015 Mar 25;13(3):e1002110. doi: 10.1371/journal.pbio.1002110 (PMC4373802; doi:10.1371/journal.pbio.1002110)
Supplement: S4 Text — (PDF) [file pbio.1002110.s004.pdf]

Optics and Devices Lab Course

General Information

Textbooks:
Practical Electronics for Inventors, 2nd ed. by Scherz
Fundamentals of Light Microscopy, Spencer
Measurements and their Uncertainties, Hughes and Hase

Course overview

This course provides a hands-on introduction to designing, building, and evaluating devices for controlling experiments in the field of bioengineering. This course focuses primarily on the tools and concepts related to optics and electronics but also touches on other valuable techniques such as rapid prototyping, image analysis, and microfluidics. The first part of the course consists of guided modules such as building microscopes and electronic circuits, while the second half of the course is more open and project-based. Students will design and develop their own computer controlled microscope and biotic gaming system.

Learning objectives of the course (General)

- Learn to design and build devices to control and measure biology
- Become a scientist: learn how to make precision measurements, design experiments and analyze data.
- Become an engineer: learn creativity, develop key skills, get things to work, and get an introduction to the design process.
- Learn how to document your work in the laboratory.
- Gain experience with instrumentation and tools. Become comfortable in a laboratory setting, make things work with what is at hand, and know what tools are out there.
- Gain experience completing an open-ended team project.
- Become an independent learner / be able to find and utilize resources on the web
- Have fun!

Learning objectives of the course (Specific)

- Be able to enter any BIOE (or other) lab and build arbitrary experimental setups/devices/structures across scales ranging from 0.1mm-1m.
- Interface with (micro-)biological systems by controlling light / electric signals / fluids /mechanical motion
- Integrate actuators and sensors into your setups (motors, LEDs, camera, …)
- Interface equipment with the computer
- Write programs (Matlab and Arduino native environment) that permit actuation of devices and recording of data (i.e. automate experiments)
- Be able to properly analyze your data and perform error analysis
- Learn a suite of tools for achieving the above goals (analog electronics, oscilloscopes, CAD, basic prototyping techniques)
- Get an introduction to the above topics, and identify key areas for future study to increase depth

Course structure

- 20 modules, 4 hours each + exams week (see table below).
- Monday lectures provide practical instruction for completing labs

- The first 12 modules focus on measurement, electronics, optics, and micro-fluidics.
- The last 8 modules are dedicated to your project and include additional topics such as object tracking, rapid prototyping, microcontrollers, etc.
- The ultimate goal of the project is to develop an integrated setup for tracking and controlling swimming cells inside a chamber via computer and human interaction (a biotic game). Working devices are intended for educational outreach in schools and museums, which is good for STEM education, future NSF fellowship applications, and your own enjoyment.
- Students work in teams of two. Each student will keep their own notebook, and do their own assignments, but teams only have to hand in one final project report.

Week	Lecture topic	Lab Topic (Modules)	Key Concepts
1	Intro, error analysis, basic electronics	Intro	Intro/Safety / Measurement/Error analysis, multimeter
		Electronics 1	Ohms Law, LEDs, multimeter
2	Electronics continued	Electronics 2	RC Filters, Function Generator
		Electronics 3	Op Amps, amplifiers, buffers, band-pass filter
3	No lecture	Electronics 4	Arduino microcontroller, programing basics, I/O
		Electronics 5	Driving motors with Arduino, mosfetts, PWM
4	Introduction to optics	Optics 1	Optics basics, build an infinity space microscope
		Optics 2	Focal lengths of lenses, Kepler & Galilean telescopes
5	Optics continued	Optics 3	Numerical Aperture, resolution, Point Spread Function, conjugate planes, Kohler illumination
		Optics 4	Darkfield & oblique illumination
6	Optics and electronics concepts review	Optics 5	Using mirrors, imaging lab with biological specimens, recording Brownian motion
		Microfluidics	Build flow chamber; observe microorganisms (Euglena)
7	Event driven programming and image analysis	Project 1	Project introduction; light microscope; start project webpage
		Project 2	Object tracking and recognition with Matlab
8	Prototyping / Machining	Project 3	Build out full control with Arduino
		Project 4	Rapid prototyping, Machining; CAD and 3D printer
9	Discussion bioethics, game design, outreach	Project 5	Discuss final project write-up and presentation; build out full system
		Project 6	Free project time
10	No lecture	Project 7	Free project time
		Project Presentation	Final project presentation

Module structure

The four-hour modules typically start with a brief introduction to the lab exercises for the day. After the lab intro, students are dismissed to the lab.

For each lab module there is a small problem set to test your knowledge of the relevant material. The problem sets are turned in at the start of the lecture, and will be returned to the students to

use in the labs when relevant. Notebooks are typically handed to the instructors at the end of the four-hour period for grading and final discussions. Lab time may exceed four hours on a voluntary basis, with instructor approval, especially during the project phase.
