# Supplementary material for: A Biotic Game Design Project for Integrated Life Science and Engineering Education
Source: PLoS Biol. 2015 Mar 25;13(3):e1002110. doi: 10.1371/journal.pbio.1002110 (PMC4373802; doi:10.1371/journal.pbio.1002110)
Supplement: S6 Text — (PDF) [file pbio.1002110.s006.pdf]

Measurement and Error Analysis

Learning goals:

- Recapitulating basic measurement and error analysis skills
- Recapitulating basic electricity and working with a multi-meter

Homework / reading prior to lab

- Refresh following terms:
- Mean, STD, SEM, Median
- Types of errors (systematic, random, human, others); precision vs. accuracy
- Gaussion distribution / confidence intervals; sigma vs. %)
- Non-gaussian errors
- Central limit theorem
- Taylor expansion
- Propagation of errors
- Weighted mean
- t-test

- Ohm's law;
- Resistance of wire (geometry + material)
- Total resistance for serial and parallel arrangement of resistors

Test questions for understanding:

- Give definitions / equations for all terms above
- Compute Mean, STD, and SEM for 1, 3, 3, 2, 1 by hand
- Assume you measured the variables x, y, and z incl. their uncertainties Δx, Δy, Δz. What is the uncertainty of the dependent variable f(x,y,z)? Give the general answer, and derive for the following functions: f(x,y,z)=x^2/y^3/z^(3/2); f(x,y)=1/x+1/y; f(x,y,z)=[x^2-y^2)]/[y-z]

1. Taylor, John R. An Introduction to Error Analysis: The Study of Uncertainties if Physical Measurements. University Science Books, 1982.

2. Hughes and Hase; Measurement and their uncertainties – A practical guide to modern error analysis; Oxford University Press

3. http://itl.nist.gov/div898/handbook/

Laboratory Instructions

Date

Objective

- Measure the height (perpendicular to the paper!) of a pencil line in two independent ways and compare your results
- This is a very simple experiment enabling to focus on the philosophy of experimentation, error analysis, and notebook keeping
- Make measurements of high precision and accuracy, and compare both methods. Pay attention to meaningful trade-offs: What is the effort vs. what is gained?
- For consistency all students will use the 0.9 mm diameter 2H lead provided.

Background

When drawing with a pencil, the “lead” shortens as graphite is deposited on the paper. Assuming volume conservation (and what else?), it is possible to estimate the line height. The “lead” of a pencil is made primarily of graphite, which is an electrical conductor. This means that a pencil line drawn on paper will also conduct electricity. Using a couple of assumptions (which), the measured resistance of the drawn lines permits estimation of the line height.

Fun fact:

Pencil “leads” also makes great and in-expensive bio-compatible electrodes (although they break easily).

Parts List/Materials

- Ruler (~20 cm)
- Caliper
- Paper
- Pencils with 2H, 0.9 mm diameter lead
- Calculator / computer for computational analysis (contributed by student)
- Digital multimeter (for measuring resistance)

Experiments/Tasks

PLAN EXPERIMENT (15 min)

All experiments should begin with some thought and planning related to the task at hand. What is the desired quantity to measure? What are potential strategies that can be used to achieve this measurement? In our lab we want to estimate the height of the line by measuring the shortening of lead for a given line length, as well as utilizing the electrical resistance of the line.

A few tips:

We found that lines drawn on paper look surprisingly different under high magnification (50x) than they do to the naked eye. Magnified lines show a tremendously porous structure, with many gaps in the line. Often, a single stroke of the lead will not conduct electricity due to these gaps. Multiple strokes can fill in these gaps to the point that the lines conduct. We found that 10 strokes make consistently conducting lines.

We recommend that all teams use lines that are made from 10 overlaid strokes for consistency purposes when measuring using the resistance technique. Consider how this alters your assumptions in measuring the line height, and what other sources of variation this introduces.

We also found that electrical connections with the lines can be difficult to make due to the low contact area. You may want to use the provided paperclips, alligator clips, or both to stabilize the connections between the meter and the drawn lines.

- Create a procedure for measuring the shortening of the lead in the pencil as lines are drawn to estimate the height of the line. What assumptions are you making?

- Create a procedure for measuring the height of the line via measurements of electrical resistance. What assumptions are you making? A good way to make these measurements is to measure the resistance of drawn lines to varying lengths. A plot of resistance versus length should be linear (use a best fit line), and the slope will be inversely proportional to the line thickness. To improve consistency it is best to draw one long line, and measure the resistance between a fixed probe on one end, and another that can be positioned at different locations along the line.

CRUDE TEST OF EXPERIMENTAL SETUP (15 min)

- Draw some lines on paper

- Quickly test continuity. Do they consistently conduct? Can you make measurements of the resistance? Can you estimate what length of lines you should use to get high quality measurements with your multimeter? Get some practice using the multimeter.

- Draw long lines. Can you measure the lead shortening? Can you estimate how long the lines should be to make a good measurement based on the precision of the provided equipment?

- Adjust your protocol if needed based on these crude tests.

ESTIMATE LINE HEIGHT BASED ON LEAD SHORTENING (30 min)

- Draw lines and measure shortening of lead.
- Make at least five independent measurements

- Estimate the thickness of the line based on the 5 measurements. What is the mean and stdev of your data?

- Error analysis

ESTIMATE LINE HEIGHT BASED ON ELECTRICAL RESISTANCE (60 min)

- Draw lines and measure the resistance verses line length
- Make at least 5 independent measurements

- Are the trends linear? Is the y intercept 0? If not, why do you think that is?

- Measure resistance of intact lead to calculate resistivity. How does this compare to the published value in Scherz?

- Estimate the thickness of the line based on the slope of the trends from the 5 measurements. What is the mean and stdev of your data?

- Error analysis

SUMMARY AND DISCUSSION (30 min)

- Summarize results
- What are the random and systematic errors?

- Do the estimated thicknesses match between the two experimental techniques?

- Discuss agreements / disagreements / potential reasons, for example: What are the biggest sources of errors? Where could additional effort lead to improvement? Is such improvement possible with the tools at hand? Which of your assumptions are probably not true / would need further testing? What measurement tools should be changed? etc.

- Suggest other experiments to measure the line height (for example optics based)

- Suggest improvements for this module / instructions set for next year’s class

- Please list the amount of time you spent on each section of this lab

BONUS (if time allows)

- Perform other measurements that seem relevant, such as testing pencils of different width, do serial and parallel circuits of pencil lines behave as expected etc.
