# Supplementary material for: A Biotic Game Design Project for Integrated Life Science and Engineering Education
Source: PLoS Biol. 2015 Mar 25;13(3):e1002110. doi: 10.1371/journal.pbio.1002110 (PMC4373802; doi:10.1371/journal.pbio.1002110)
Supplement: S8 Text — (PDF) [file pbio.1002110.s008.pdf]

BIOE 123 Module 2

Electronics 1: Voltage, Resistance, Current, and Power

Lecture (30 min)

Date

Learning Goals

- Become familiar with Ohm's law, Kirchhoff voltage and current laws
- Learn how to find the equivalent resistance of a circuit
- Learn useful configurations of resistors for generating specific voltages
- Calculate electrical power
- Learn to properly use diodes and LEDs
- Get hands-on experience using tools like breadboards, power supplies, and a multimeter

SUMMARY OF TOPICS (actual lecture notes attached as separate document)

- Electronics Basics (hydraulic analogies)
  - Voltage, Resistance, and Current
- The correct form of Ohm's law
  - $\Delta V = I*R$… very important and subtle difference
  - Measuring V, R, and I. Potential errors and tradeoffs
- Ground
  - What it means, and why it is important
  - Height analogy with stairs
- Resistor Properties
  - What they look like, measured in Ω, how to measure
  - Resistors in series and parallel
- Kirchhoff current law (KCL)
  - Currents at a node sum to 0
- Using Ohm's law and KCL to decode circuits
  - Use the "two probe" technique, simulate use of a multimeter
  - Sequential generation of multiple equations, then combine and solve
- Diodes
  - Unidirectional conduction, forward voltage, current limiting resistor

Lab Intro (~15 min)

Thursday January 9, 2014

SUMMARY OF TOPICS

- Electronics prototyping
  - The breadboard, how the pins are arranged, the power rails
  - Breadboard layout
- The multimeter, measuring ΔV, I, and R
- Electrical power
  - $P = I*\Delta V$, for resistors = $(\Delta V^2)/R$ or $(I^2)*R$.

Electronics 1: Voltage, Resistance, Current, and Power

Problem Set

Due: Date.

Text: Practical Electronics for Inventors, Scherz

Estimated reading time: 1 hr for Core reading, additional 1 hr for optional reading

Learning Goals

- Become familiar with Ohm's and Kirchhoff's laws. How does a ΔV induce a current across a resistance.
- Understand the concept of "ground" in a circuit
- Learn how to find the equivalent resistance of a circuit
- Calculate electrical power
- Learn how to measure resistance, voltage, and current with the multimeter

CORE READING
2.2: Electric Current
2.3: Voltage
2.5: Resistance, Resistivity, Conductivity
2.10: Grounds
2.11: Electric Circuits
2.12: Ohm's Law and Resistors

OPTIONAL READING
2.17: Kirchhoff's Laws
2.14: Measuring Voltage, Current, and Resistance
2.7: Heat and Power
2.13: Voltage and Current Sources
2.15: Combining Batteries
2.16: Open and Short Circuits

PROBLEMS

Problem 1: For circuit A from the diagram, calculate the following quantities:

- The voltage at **a**, **b**, **c**, and **d**. Calculate all values relative to ground.
- The total current drawn by the circuit.
- The power dissipated by the circuit.
- List the resistors in order of the voltage drop they experience from smallest to largest.

Problem 2: For circuit B from the diagram, calculate the following quantities:

- The currents I_T, I_1, I_2, and I_3.
- The power dissipated by the circuit.
- List the resistors in order of the current they pass from smallest to largest.

Problem 3: For circuit C from the diagram, calculate the following quantities:

- The relationship between V_O and R_1 and R_2.
- Calculate the ratio of R_2 / R_1 that yields values for V_O of 1V, 2.5V and 4V, respectively.

Problem 4: For circuit D from the diagram, calculate the following quantities:

- Determine the value of R_3 that will limit the current through the LED to 10mA.
- Does it matter if you switch the order of the resistor and LED?

Problem 5

You are given a resistor of unknown value. You have a power supply that can vary from 0-5V at the output. You perform and experiment where you set a particular voltage on the supply, and then measure the current through the resistor. You record the following measurements of current across the resistor at discrete voltages:

Voltage (V)	Current (A)
1	0.079
1	0.083
1	0.079
2	0.160
2	0.173
2	0.168
4	0.319
4	0.320
4	0.342
5	0.431
5	0.402
5	0.415

- Produce an analysis that will estimate the resistor value based on the provided data.
- If we neglect error in the current measurement, what is the likely "source of error"?
- Can you estimate the absolute range of error in the "source of error"?

- Suggest an alternate experiment that can potentially reduce the error in the measurement of the resistance. What assumptions would you have to satisfy for your experiment to work?

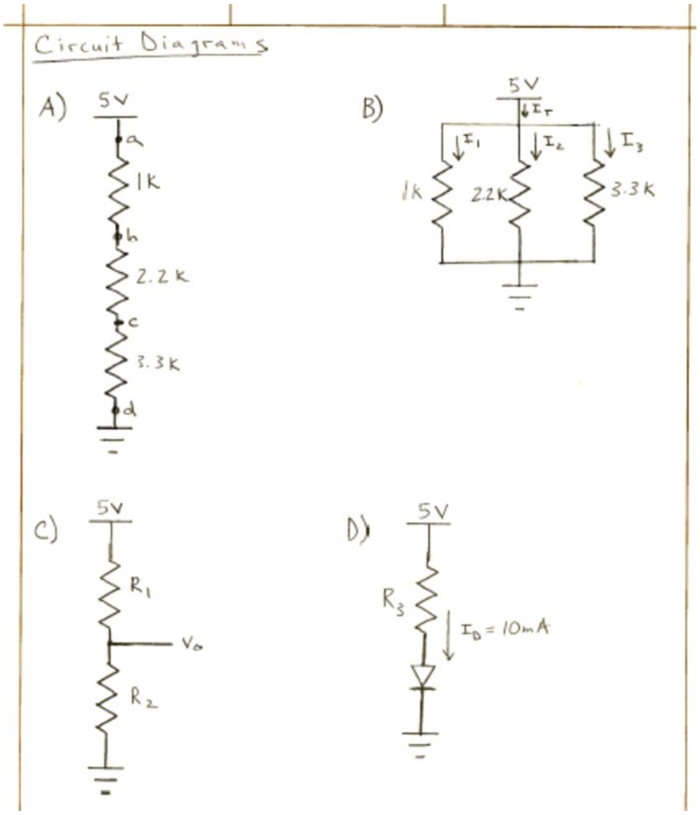

Electronics 1: Voltage, Resistance, Current, and Power

Laboratory Instructions

Date

Objective

- Familiarize yourself with the electronics lab equipment, including power supplies, multimeters, and the electronic components we have for you
- Gain experience prototyping circuits with a breadboard, including LEDs
- Learn to generate desired resistor combinations out of what's available in lab
- Learn to consider electrical power when designing a circuit
- See how voltage in actual circuits can differ from the theoretical value

Background

These next few modules will focus more on the "engineering" side of the course, helping you learn how to leverage a few scientific principles to design a circuit you could use in a project or experiment. You're not off the hook with error calculation, however, and you should do so when appropriate.

Resistors are an extremely important tool, and learning how to form specific resistor values out of others in a pinch will help you out later (particularly in module 3). LEDs are a great way to add polish to a project and can give you a visual indication of how something is performing, especially when you don't have an oscilloscope handy. Today you will learn not only how to avoid blowing them up, but we will also use them to explore electrical power.

Finally, you will also become comfortable building real circuits in a breadboard and making measurements with a digital multimeter. Practice keeping your circuits as neat as possible, as you will be building more involved ones later and it will make debugging a lot easier.

Parts List/Materials

- Breadboard
- Assorted jumper wire box
- Power supply
- 1k, 2.2k, 3.3k and various other resistors from the public box
- 10k Potentiometer
- LEDS
- Digital multimeter

Experiments/Tasks

RESITOR NETWORK IN SERIES (45 minutes)

For the first experiment, you will be making predictions about a series resistor network circuit and checking them using a multimeter. Resistors in series add, so this type of circuit can be used to “build” a larger value resistor out smaller ones. It also divides the voltage in increments related to the resistance values used which can be a useful way to step down voltages to desired values lower than the available power supply provides. Larger resistors will experience larger voltage drops due to ohm’s law.

- Build the circuit in diagram A on your breadboard. Check resistor values with the multimeter (remember they must be disconnected from power and other components). Have a member of the teaching staff inspect your circuit before powering it up. Note this is the circuit from problem 1 of your homework.
  _____TA check

- Record the expected, actual voltage, % error at each location:

Location	Expected V	Actual V	% Error
A			
B			
C			
D			

- How close did you get? What could be contributing to the observed error?
- Measure the total current drawn by this circuit. What is the % error between the measured value and your prediction from the homework? How close did you get? What could be contributing to the observed error?

RESITOR NETWORK IN PARALLEL (45 minutes)

In this experiment, you will be making predictions about a parallel resistor network circuit and checking them using a multimeter. Adding resistors in parallel reduces the overall resistance of the network by providing multiple paths through which current can flow. These types of circuits can be used to “build” an equivalent resistance that is lower than the values you have available. Larger resistors will experience reduced current based on the tendency of current to flow through the “path of least resistance”.

- Build the circuit in diagram B on your breadboard. Have a member of the teaching staff inspect your circuit before powering it up. Note this is the circuit from problem 2 of your homework.
  _____TA check

- Record the expected currents I_T, I_1, I_2, I_3. Measure the actual currents I_T, I_1, I_2, I_3 using two methods. First, use a direct current measurement with the multimeter. Second, calculate the current by measuring the voltage drop across each resistor and its actual resistance with the multimeter.

Location	Expected I	Actual I (method 1)	Actual I (method 2)
I_T			
I_1			
I_2			
I_3			

- How different were the two methods? How different are the measured resistance values from the factory specified values? Does this agree with the advertised tolerances of the resistors?

CREATING A VOLTAGE WITH THE VOLTAGE DIVIDER (45 minutes)

For the next task, you will use resistors in a voltage divider configuration to get as close as possible to a new desired voltage level below the standard 5V of your power supplies. The simple voltage divider consists of two resistors, and the voltage is measured at the junction between them. In this section, you will build the circuit in diagram C on your breadboard. The values of R_1 and R_2 are unspecified so you can choose your own resistors. Pick a pair of resistors such that the predicted V_O is 1V. If you can't find a pair of resistors with the proper values in the box, you can choose the closest ones or you can combine multiple resistors in series/parallel to get closer to the calculated values and yield a better approximation of the desired V_O. **In either case, the total equivalent resistance from 5V to ground must be larger than 1k.**

- Draw the circuit and show a member of the teaching staff. _____TA check

- Repeat the previous step, but produce a V_O of 4V. Draw the circuit.

- Repeat the previous step, but produce a V_O of 2.5V. Draw the circuit.

- For your 2.5V circuit, how close did you get to the desired voltage? Analyze how resistor tolerances might have affected your result using error analysis.

- Bonus: achieve the same result using the 10k potentiometer. (draw the circuit)

LIGHTING AN LED (30 min without bonus)

Finally, let's light some lights. You will see how power directly affects LED brightness in various configurations.

- Build the circuit in diagram D on your breadboard. Pick a value for R_3 that limits the current through your LED to 10mA. You can find the forward voltage of the LED on the provided data sheet. If you don't have a resistor that matches the desired value, use the closest LARGER resistor available. Recalculate the expected current requirement based on this resistor. Check that your LED lights up, and show a member of the teaching staff. _____TA check

- Measure the current drawn by your circuit. Record the % error in your actual current.

- Bonus: Calculate a resistor value to light two of the same LEDs in series, and in parallel with 10mA of current in each. Calculate the power dissipation and make predictions about LED brightness in either situation. Build the actual circuit and determine the following: Are they the same brightness? If not, what is the source of the difference?
