# Supplementary material for: A Biotic Game Design Project for Integrated Life Science and Engineering Education
Source: PLoS Biol. 2015 Mar 25;13(3):e1002110. doi: 10.1371/journal.pbio.1002110 (PMC4373802; doi:10.1371/journal.pbio.1002110)
Supplement: S9 Text — This module provides homework and lab instructions for the second electronics unit involving filters. (DOCX) [file pbio.1002110.s009.docx]

**BIOE 123 Module 3**

**Electronics 2: Time Varying Circuits**

**Lecture (30 min)**

Date

**Learning Goals**

- Learn about the behavior of capacitors and inductors
- Learn how to analyze time-varying circuits to quantify parameters
- See the effects of first-order RC filters
- Learn how to use capacitors and resistors to tune to a particular time constant
- Learn how to use the oscilloscope to measure signals, particularly the auto-measuring capabilities and the cursors
- Use the function generator for the first time

**SUMMARY OF TOPICS** (actual lecture notes attached as separate document)

- Capacitors
  - Symbol, physical understanding, units, capacitance equations
- Impedance and voltage across capacitor
- Time constants
  - Solving the RC Circuit for charging and discharging
- Complex signals
  - AC, sinusoidal waves, Fourier series, square waves
  - RC circuit effect on square wave
- Filters
  - Bode plot
  - Corner frequency
  - Low-pass, high-pass, band-pass, notch
- Inductors
  - Symbol, physical understanding, units, inductor equations
  - Inductors in filters
  - RLC circuit

**Lab Intro (~15 min)**

Date

**SUMMARY OF TOPICS**

- Basic equipment
  - Oscilloscope
  - Function generator
  - Demo of RC charge/discharge and emphasize cursor use
- Reminder about filters
- Bode plot – more detail, and how to make

**Electronics 2: Time Varying Circuits**

**Problem Set 3**

Due: Date at the end of lab.

Text: Practical Electronics for Inventors, Scherz

Estimated reading time: 1 hr for Core reading, additional 30 min for optional reading

**Learning Goals**

- Learn about the behavior of capacitors and inductors
- Learn how to analyze time-varying circuits and quantify basic parameters
- See the effects of first-order RC filters
- Learn how to use capacitors and resistors to tune to a particular time constant

CORE READING

2.20: AC circuits

3.6: Capacitors (up through 3.6.4, plus 3.6.9)

3.7: Inductors (up to 3.7.4)

8.1: Things to Know Before You Start Designing Filters

8.2: Basic Filters

8.3: Passive Low-Pass Filter Design

8.4: A Note on Filter Types

Download the program and build the following two circuits. Pay careful attention to the direction of the electron flow and accumulation upon connecting components.

http://phet.colorado.edu/en/simulation/circuit-construction-kit-ac


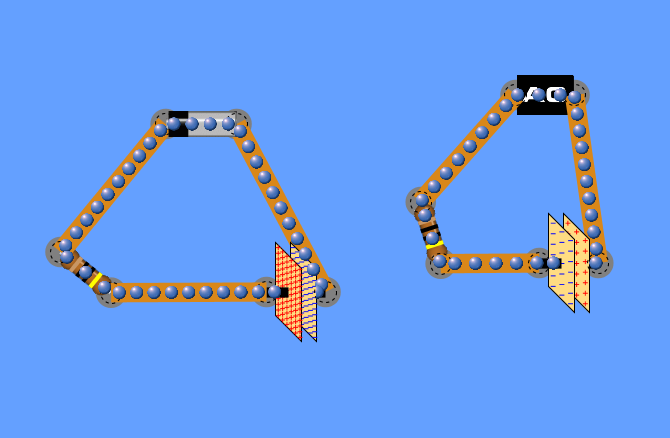


PROBLEMS

Reasonable values for C: 0.001-1 uF

Reasonable values for R: 1k-100k Ohms

1. Design and draw the circuit for a passive RC high-pass filter with corner frequency of 159 Hz. (Be sure to specify Vin and Vout and select reasonable values for R and C) Draw the frequency response plot. (be sure to properly label axis and indicate corner frequency)
2. Design and draw the circuit for a passive RC low-pass filter with corner frequency of 1590 Hz. (Be sure to specify Vin and Vout and select reasonable values for R and C) Draw the frequency response plot. (be sure to properly label axis and indicate corner frequency)
3. A student connects the two circuits from 1) and 2) such that Vout of 1) is connected directly to Vin of 2) in an attempt to create a bandpass filter. Draw this circuit. The frequency response doesn’t seem to match what he predicted based on the two independent circuits. Why might this be the case?
4. How long did the reading take? How long did the homework take? Any additional feedback on these materials? Ex: book readings were clear, or not, HW was too easy or hard?

**Electronics 2: Time Varying Circuits**

**Laboratory Instructions**

Date

Location:

Personal (“collaborators”): Work in pairs. Supervision by instructor and TAs.

**Objective**

- Learn how to use the function generator
- Learn how to use the oscilloscope to measure signals, particularly the auto-measuring capabilities and the cursors
- Learn about the behavior of capacitors
- See the effects of first-order RC filters
- Learn how to use capacitors and resistors to tune to a particular time constant
- Learn how to analyze a simple RC circuit
- (Note: The other typical major component of time varying circuits is the inductor, but we will not be working with that today.)

**Background**

In today’s lab you will get used to using capacitors in circuits, as they are an important tool to use when a signal changes with time (which will be most signals of interest). You will examine how a circuit's time constant affects its output, as well as how a filter's R and C values will change signals of various frequencies. The capacitors we are working with aren’t polarized, but be aware that polarized capacitors exist. The first section of the lab introduces you the equipment and electric components. As your proficiency increases, you should find that you can rapidly build these circuits and take measurements using the tools. As always, please ask a member of the teaching staff if you are stuck or have questions.

*Note: in order to focus on learning to use equipment and filters, a full error analysis section will not be required for today’s lab.

**Parts List/Materials**

- Breadboard (Jameco Electronics)
- Oscilloscope (Tektronix TDS 1012C-EDU)
- Function generator (GW Instek GFG-8020H)
- Jumper wire (Jameco Electronics)
- Various resistors and capacitors (Jameco Electronics )

**Planning phase** (15 min)

- The goal of today’s unit is for you to learn skills and tools, rather than making a scientific measurement. Therefore you can keep note keeping to a minimum; no error analysis needed if not explicitly required
- Read the complete experiment instructions
- Prepare your notebook (again – keep to the essential minimum – in large you can refer to these instructions)
- Check that all tools and materials are ready
- Make a time plan (we already did that for you) and add into your notebook
- Are there any safety concerns?

**Experiments/Tasks (execution phase)** (130 min)

1. Familiarization with the function generator and oscilloscope? (30 min)

*For questions about how to use the function generator, refer to the manual, the “First time operations instructions” section starting on page 10 may be beneficial.

- Set the function generator to deliver a 1 kHz sine wave with peak-to-peak voltage of 1 V. Measure the peak to peak voltage and the frequency using the oscilloscope.
- Measure peak-to-peak (p-p) voltage using the cursors
- Measure p-p voltage using the automatic measure function of the oscilloscope. How would you report error for the p-p voltage? For the frequency?
- Play around with generating various frequencies, amplitudes, wave forms, and offsets with the function generator and observing and measuring various quantities with the oscilloscope. (free play time!)
- Show a member of the teaching staff you know how to use this equipment

_____TA Check

2. For today's first experiment, you will receive a capacitor and build a circuit to determine its capacitance. (40 min)

- Build a high-pass filter using a 10 kΩ resistor and the mystery capacitor. Draw this circuit
- Supply this circuit with a Sine wave from the function generator. Record peak-to-peak voltage vs. frequency. Use these values to make a Bode plot and show the frequency response of this circuit. (Using a computer with spreadsheet/plotting capabilities here is helpful, and these plots can be very quick to make). Use at least 10 frequency values to fully capture the response.
- Report the corner frequency__________________
- Determine the mystery capacitance (Check with a member of the teaching staff when you have a guess) Bonus: What is your uncertainty / error on this capacitance?

_____TA Check

- Based on your reading, think about another way to measure this capacitance.

*Leave this circuit on the breadboard; it will be used in part 4.

3. For the next experiment, you will build a low-pass filter. Design a filter with corner frequency of 1590 Hz, using a 0.001 uF capacitor. (20 min) Draw this circuit.

- Create another Bode plot as in 2) to show the frequency response of this circuit.
- Report and compare the corner frequency with the targeted value. Discuss whether any discrepancy is likely due to resistor error, capacitor error, or error in measurement.

*Leave this circuit on the breadboard; it will be used in part 4.

4. Now you will combine the circuits from 2) and 3) to make a band-pass filter. To do this, connect the output from the high-pass filter to the input for the low-pass filter and measure the output in the same position you did for the low-pass filter. (20 min)

- Create another Bode plot as in 2) and 3) to show the frequency response of this circuit.
- Describe in words the frequency response.
- What is the least attenuated amplitude (largest V p-p output/V p-p input)?
- What is the bandwidth of this circuit?

For your report, include the Bode plots from each filter from 2) and 3) superimposed on the band-pass filter’s Bode plot

*Note: Leave this circuit on your breadboard; you’ll modify it in the next section.

5. In the circuit from 4) now replace the low-pass filter with a low pass filter with the same corner frequency, but using a 1000 kΩ resistor instead. (20 min)

- Make a Bode plot and describe any differences between this frequency response and that from section 4).
- In your plot, include the Bode plots from each filter from 2) and 3) superimposed on the new band-pass filter’s Bode plot
- What is the least attenuated amplitude (largest V p-p output/V p-p input)?
- Assuming the new low-pass filter behaves identically to the old one in isolation, what likely caused the change?

Be sure to include diagrams for each circuit and all Bode plots in your report.

**Summary results / error analysis:** (0 min)

Not necessary for this module.

**Discussion:** (15 min)

For your discussion include one bioengineering application for each type of filter you built today (low-pass, high-pass, and band-pass).

Report how long each section took. Give one thing you especially liked from this lab, and one change that would improve the material.
