# Supplementary material for: A Biotic Game Design Project for Integrated Life Science and Engineering Education
Source: PLoS Biol. 2015 Mar 25;13(3):e1002110. doi: 10.1371/journal.pbio.1002110 (PMC4373802; doi:10.1371/journal.pbio.1002110)
Supplement: S11 Text — This module provides homework and lab instructions for the third electronics unit involving operational amplifiers. (DOCX) [file pbio.1002110.s011.docx]

**BIOE 123 Module 4**

**Electronics 3: Op Amps**

**Lecture (30 min)**

Date

**Learning Goals**

- Learn basic applications of op amps
- See common circuit topologies with op amps
- Design a circuit to translate an input signal to desired output levels
- Learn wiring up chips and reading datasheets

**SUMMARY OF TOPICS** (actual lecture notes attached as separate document)

- Intro to op amps
  - Symbol
  - Idealized behavior
  - Crude comparator function
- Negative Feedback
- Golden rules of Op Amps (in negative feedback)
  - No current through inputs, V_+_ = V_-_
  - Review KCL for tracking the current path through a circuit
- How to analyze Op Amp circuits
  - Do an example (inverting amplifier)
  - Talk about how to set a dc offset for the output if desired (using a pot is easiest)
- Saturation
  - Op Amps are bound in output by their power rails

**Lab Intro (~15 min)**

Date

**SUMMARY OF TOPICS**

- Op amp reminders/answer questions from Pset
- Reading data sheets for chips:
  - Op Amp
  - Photodiode
- Placing and removing ICs on the breadboard
  - Top notch, pin numbering
  - Chip puller/pen/DMM lead for removal
  - Check, power, touch, smell
- Reminder about how to use the pots

**Electronics 3: Op Amps**

**Problem Set 4**

Due: Date at the end of lab.

Text: Practical Electronics for Inventors, Scherz


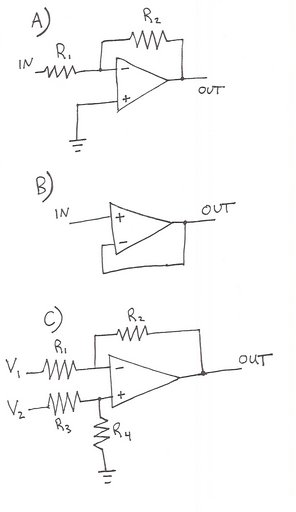
Estimated reading time: 45 min for Core reading, 30 min for Optional reading

**Learning Goals**

- Learn basic applications of op amps
- See common circuit topologies with op amps
- Learn to analyze op amp circuits

CORE READING

7.1: Operational Amplifier Water Analogy

7.2: How Op Amps Work (The “Cop-Out” Explanation)

7.3: Theory

7.4: Negative Feedback

Browse the Wikipedia page on Op Amp Applications.

OPTIONAL READING

7.12: Comparators

7.13: Comparators with Hysteresis

PROBLEMS

1) What is the name of the circuit pictured in ‘A’? (1 pt)

If R_1_ is 2 kΩ and R_2_ is 3 kΩ, what is the gain? (1 pt)

If 1V is put at ‘IN’ what is the voltage at ‘OUT’? (1 pt)

2) Given 2 V at ‘IN’ what is the voltage at ‘OUT’ in B)? (1 pt)

In words, what does the circuit in ‘B’ do? (1 pt)

What is one potential use for this circuit? (1 pt)

3) A student is making a differential amplifier as in ‘C’.

In words describe what this circuit does. (1 pt)

If a student mistakenly makes R2=R4 and R1≠R3 what is the voltage at ‘OUT’ with inputs ‘V1’ and ‘V2’? Solve analytically. (2 pts)

4) How long did the reading take? How long did the homework take? Any additional feedback on these materials? Ex: book readings were clear, or not. HW was too easy or hard? (1 pt)

**Electronics 3: Op Amps**

**Laboratory Instructions**

Date

Location:

Personal (“collaborators”): Work in pairs. Supervision by instructor and TAs.

**Objective**

- Develop intuition about op amp circuits
- Learn to use chips on the breadboard and read datasheets
- Practice using the function generator and oscilloscope
- Learn how to wire up an op amp and calculate gain and offset
- Explore op amp saturation
- Design a circuit to translate an input signal to desired output levels
- Build a cool circuit and figure out its behavior

**Background**

Today you will be working with operational amplifiers (Op Amps) which have many useful applications, as you saw in the reading. Along with filters, they are an invaluable tool for manipulating a wide range of signals. Often, a signal you are sensing gives very small differences in voltage over the range you are testing. An op amp is useful for (as the name implies) amplifying a signal so that whatever is recording the data will not be limited by its own resolution. For example, when an analog signal is digitized, only a certain number of bits are used to represent the number. So if 0V corresponds to 0, and 5V to 1023 (10 bit), you can get much more resolution out of a signal that varies from 100 to 800, than from 100 to 150. Of course, you also amplify any noise in the signal, which is where your filters and buffers come in…but we won't worry about that for today.

Your second task today is to build a simple tachometer, using an op amp as a crude comparator. You will use this circuit to measure the no load speed of a DC motor driven at 5 volts. This circuit is fun to play with, and will give you some experience wiring up an op amp for the purposes of measuring an unknown quantity.

**Parts List/Materials**

- Breadboard (Jameco Electronics)
- Oscilloscope (Tektronix TDS 1012C-EDU)
- Function generator (GW Instek GFG-8020H)
- Jumper wire (Jameco Electronics)
- Various resistors, potentiometers (Jameco Electronics )
- Lab power supply (Mastech DC Power Supply HY3003D-3)
- LM324N op amps (Jameco Electronics)
- Phototransistors (Jameco Electronics)

**Experiments/Tasks**

Inverting Amplifier

- Generate a sine wave that is 1V peak-to-peak, and centered around 2V. Frequency doesn't matter too much, so use something on the order of a few kHz. Check the voltage levels on the oscilloscope and adjust the signal using the amplitude and offset knobs on the function generator.
- Design your op amp circuit. Aim to get an inverted wave that goes from 1V to 7V (so, a gain of 6 with some offset that you should calculate). Use the inverting configuration and show your calculations. _____TA check

Circuit schematic:

- Wire up your chip, using your 5V power supply as power to the chip, and the function generator as the input. Remember to connect the function generator ground to your circuit ground! You can use a 10K pot to produce the offset voltage at the inverting input.

Draw the input and output waveforms, making note of the voltage at key points.

(Use the other channel on the scope to compare the two.)

- The phenomenon you see is referred to as saturation. Since the op amp can't output voltage levels outside the range of its power supply, which in this case is 0-5V, the wave is getting cut off where it should be going up to 7V. To achieve the gain we want, we're going to have to change the supply voltage.

The maximum voltage we can use is 32V. What voltage you should use to power the chip, without making it too high? Allow at least 1V of wiggle room to avoid saturating again. You will use the variable side of the power supply for this step.

Voltage = _______________

- Before you connect your circuit to power, check the voltage level on the variable side of the supply and make sure it is set properly with a multimeter. Turn off the power and connect your circuit, using the appropriate ground knob (ask us if you need help), then turn power back on.

Show a member of the teaching staff the output without saturation. _____TA check

Draw the new output of your circuit.

How close did you get to your calculations?

Op-amp Voltage Comparator


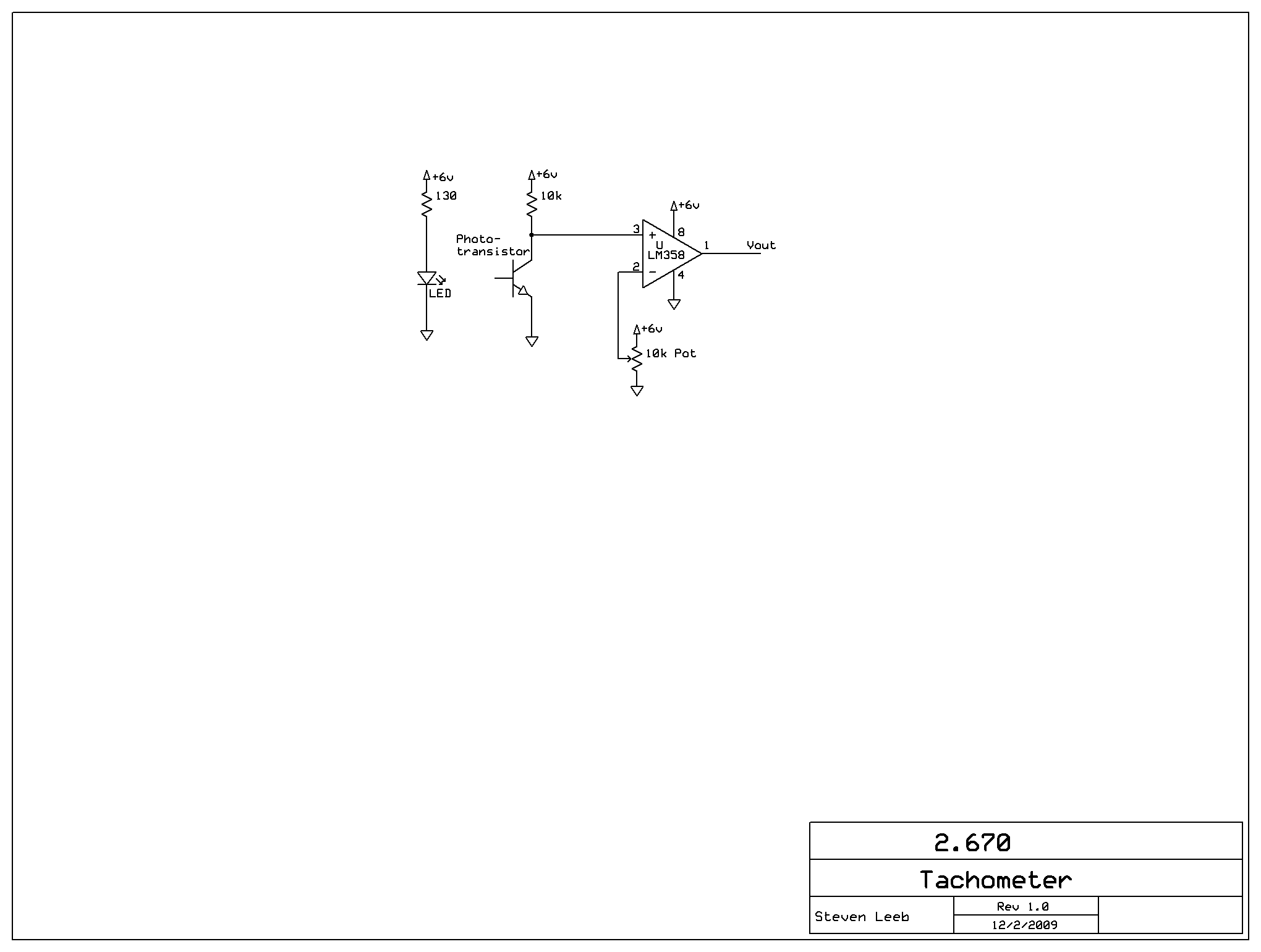


Build the circuit pictured above (the down arrows are another symbol for ground). We have found a 47k resistor in place of the 10k works best. In this particular instance, please DO NOT trim the phototransistor leads sicne we may use them later. Also, you will use the LM324 op amps (not the LM358) and you’ll power all these circuits on 0-5V (not 6 as shown in the figure).

- This circuit is a crude comparator that switches between the high and low state when the light hitting the phototransistor changes the voltage at the non-inverting input enough to cross the value set at the non-inverting input with the potentiometer. You will use the ambient light in the room to light this circuit. Tune the POT such that V_out_ is high (near 5v) when the phototransistor is covered, but drops to near zero when exposed to the ambient light.
- Take the motor and connect it to the variable power supply with a pair of alligator clips. At 5V supply of voltage, what the minimum current required to allow the motor to run at full speed?
- Stop the motor and put a strip of tape on the motor shaft that protrudes out to one side of the shaft by an inch or so. Set the motor spinning and put it over the phototransistor (but not touching). The spinning blade should block the phototranisistor once per revolution. Adjust the size of the blade, positioning, and the POT voltage so you get a fluctuating output from your circuit. _____TA check
- Visualize the circuit output on the oscilloscope. You should see a waveform that jumps between 0-5V at a nearly constant frequency. This frequency corresponds to the speed of the motor.

What is the motor speed in Hz? (Using the cursors and the run/stop button can help).
