# Supplementary material for: A Biotic Game Design Project for Integrated Life Science and Engineering Education
Source: PLoS Biol. 2015 Mar 25;13(3):e1002110. doi: 10.1371/journal.pbio.1002110 (PMC4373802; doi:10.1371/journal.pbio.1002110)
Supplement: S14 Text — (PDF) [file pbio.1002110.s014.pdf]

BIOE 123 Module 5

Electronics 4: The Arduino Microcontroller - Programming Basics, I/O

Lecture (30 min)

Date

Learning Goals

- Become familiar with using Arduino microcontroller
- Learn how to define variables and functions
- Learn how to use library
- Learn how to program using control structures
- Good Programming Practice

SUMMARY OF TOPICS (actual lecture notes attached as separate document)

- Analog to Digital
  - What is digital?
- Microcontroller
  - What is a microcontroller?
  - Peripherals
  - Arduino Uno
- Programming Basics
  - What is a source code?
  - How to define variables
  - How to define functions
  - How to use libraries
  - How to compile your code
  - How to debug your code
- Control Structures
  - For loop
  - While loop
  - If Statement
  - Switch case
- Best Coding Practices
  - Utilizing comments
  - Sticking to the naming convention

Lab Intro (~15 min)

Date

SUMMARY OF TOPICS

- Answer questions from Pset
- Arduino
  - Pin layouts
  - Pull-down resistor
  - Current
  - setup()
  - loop()
- Analog components
  - LED, series-resistor review
  - Pushbutton switch

Electronics 4: The Arduino Microcontroller - Programming Basics, I/O

Problem Set

Due: Date at the end of lab.

Text: -

Estimated reading time: 30 min for Core reading

Learning Goals

- Learn microcontroller based circuit design considerations
- Learn to plan your code by writing "pseudocode"

CORE READING
Array: http://arduino.cc/en/Reference/Array
FOR loop: http://arduino.cc/en/Reference/For
Function Declaration: http://arduino.cc/en/Reference/FunctionDeclaration
IF/ELSE: http://arduino.cc/en/Reference/Else
setup(): http://arduino.cc/en/Reference/Setup
loop(): http://arduino.cc/en/Reference/Loop

Pseudocode: http://users.csc.calpoly.edu/~jdalbey/SWE/pdl_std.html

Reference list, don't read the whole thing:
Language Reference: http://arduino.cc/en/Reference/HomePage
Datasheet: http://arduino.cc/en/Main/arduinoBoardUno

PROBLEMS

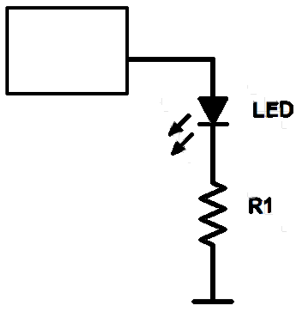

1) The Arduino has multiple pins that can serve as analog or digital inputs or outputs. To start, we will focus on the use of digital input/output (I/O) pins. When an output pin is set to "HIGH", the pin will produce a voltage of 5V relative to its ground, and can source up

to 40 mA of current. However, a safer value to use for circuit design is 20 mA, which is the half of the max current.

Suppose you want to light an LED from one of the Arduino's output pins. Assuming Vf for red, yellow, and green LEDs are 1.7V, 2.1V, and 2.2V, respectively, calculate the appropriate resistance value R1 for:
a. A red LED? (1 pt)
b. A yellow LED? (1 pt)
c. A green LED? (1 pt)

2) You want to write a program that turns on and off a row of LEDs that is already connected to an Arduino. The initial part of the code is already set for you:

```
const int redLEDRPin =  3;      // left red LED pin
const int yellowLEDRPin =  6;   // left yellow LED pin
const int greenLEDPin =  9;     // middle green LED pin
const int yellowLEDLPin =  10;  // right yellow LED pin
const int redLEDLPin =  11;     // right red LED pin
```

a. You can declare and populate an array containing the listed pin information using the following syntax:

```
int LEDarray[] = {redLEDRPin, yellowLEDRPin, greenLEDPin, yellowLEDLPin, redLEDLPin};
```

You can access a value inside the array by calling `LEDarray[integer];`. Because arrays are zero indexed, the first element of the array is at index 0. So the value of `integer` can be any integer between 0 and (the length of the array – 1). For example, `LEDarray[0] == redLEDRPin` and `LEDarray[3] == yellowLEDLPin`.

What value do `LEDarray[0]` and `LEDarray[2]` contain? (1pt)

What would happen if you call `LEDarray[5]`? (1pt)

b. You want to write a FOR loop that turns off all the LEDs in the circuit one at a time from left to right. Assume the circuit is already setup that an LED will turn off if you set your output pin value of the LED to the constant `LOW`, and will turn on if you set your output pin value to the constant `HIGH`.

You can use the function `digitalWrite()` to set the state of an output pin. Syntax for the function is `digitalWrite(pin, value);` where `pin` is the pin number and `value` can be either `HIGH` or `LOW`. For example, if you want to turn off the left red LED in the circuit, you can write a line of code:

```
digitalWrite(redLEDRPin, HIGH); // this will turn on the right red LED
```

Using the array LEDarray, write a FOR loop that will turn off all the LEDs in this circuit in succession (i.e. one at a time from left to right). It shouldn't be no longer than 3-4 lines of code. (1 pt)

c. After writing the FOR loop in part b, you encapsulated the loop into a new user-defined global function, `allLEDoff()` so the loop can be called anytime you call

`allLEDoff();`. Next, you would like to declare a new function named `controlled()`, which takes an integer variable named `numLED` and returns nothing. This function will first call `allLEDoff()` function and then turn on one LED that is listed `numLED`-th in the `LEDarray`. Write the function with a full syntax. (2pt)

3) You built your Arduino system so it would read digital inputs (`HIGH/LOW`) from a switch. The switch is connected to an input pin, and will provide `HIGH` voltage when it is pushed and `LOW` voltage when it is released. The value for the pin number is already declared as integer constants `buttonRPin`.

a. To detect how many times the switch is pushed within a full cycle of a FOR loop, you will need to declare a variable (`lastButtonRstate`) that will hold the past reading of the input pin and compare that to the current reading of the input pin. If there is a change and the current value is `HIGH`, you can increment your counter variable (`countR`).

Write a pseudocode for a function that will:

```
Read the input from the switch input pin
Compare the current state of the pin with the last state of the pin
Increment the counter variable
   (if there is a state change in the input pin
   and also if the current state of the input pin is HIGH)
Update the variable of the last state of the pin
```

The function should not take any parameters and returns nothing. (2pt)

b. Can you declare variable `lastButtonRstate` inside your function? How about `countR`? Why or why not? (1pt)

c. Write a pseudocode for a function that will:

```
Compare values of two variables, countR and countL
Increment variable LEDstate if countR is larger
Decrement variable LEDstate if countL is larger
Do nothing if they are same
```

The function should not take any parameters and returns nothing. (2pt)

Electronics 4: The Arduino Microcontroller - Programming Basics, I/O

Laboratory Instructions

Date

Location:

Personal ("collaborators"): Work in pairs. Supervision by instructor and TAs.

Objective

- Develop intuition about Arduino programming
- Learn to the hardware of Arduino Uno microcontroller
- Practice programming in Arduino IDE
- Design a circuit with various LEDs and pushbutton switches
- Make and play a game!

Background

Through the next few activities, you will start to understand how many electronic instrument around us work. You will learn how by using microcontrollers, an electronic instrument can sense the elements of its environment (using sensors), process the data (using a processor), and make necessary changes (using actuators).

You will first get warmed up by doing some straightforward microcontroller coding using the Arduino Uno, LEDs and switches. Then you will add the rest of the game logic to the system. By the end of this lab, you will be able to build a two-player game, which each player taps a pushbutton switch as fast as they can to push the LED light to the opponent's side.

Parts List/Materials

- Breadboard (Jameco Electronics)
- Arduino Uno (Arduino)
- Red, yellow, and green LEDs (Jameco Electronics)
- Pushbutton switch (Jameco Electronics)
- Various resistors (Jameco Electronics)

Useful Syntax/Functions (from Arduino reference)

- `pinMode(pin, mode);`
  Configures the specified pin to behave either as an input or an output.
  pin(int): the number of the pin whose mode you wish to set
  mode: `INPUT`, `OUTPUT`, or `INPUT_PULLUP`

- `digitalRead(pin);`
  Read the value of a digital pin.
  pin(int): the number of the pin you want to read
- `digitalWrite(pin, value);`
  Write a `HIGH` or `LOW` value to a digital pin.
  pin(int): the number of the pin
  value: `HIGH` or `LOW`
- `delay(ms);`
  Pauses the program for the amount of time (in msec) specified as parameter.
  ms(unsigned long): the number of msec to pause
- `Serial.begin(speed);`
  Sets the data rate in bits per second for serial data transmission.
  speed(long): in bits per second
- `Serial.print();`
  Prints data to the serial port as human-readable ASCII text. This commend can take many forms. Numbers are printed using an ASCII character for each digit. Characters and strings are sent as is.
- `abs(x);`
  Computes the absolute value of a number.
  x: the number

Experiments/Tasks

ARDUINO SETUP

[Figure 1]

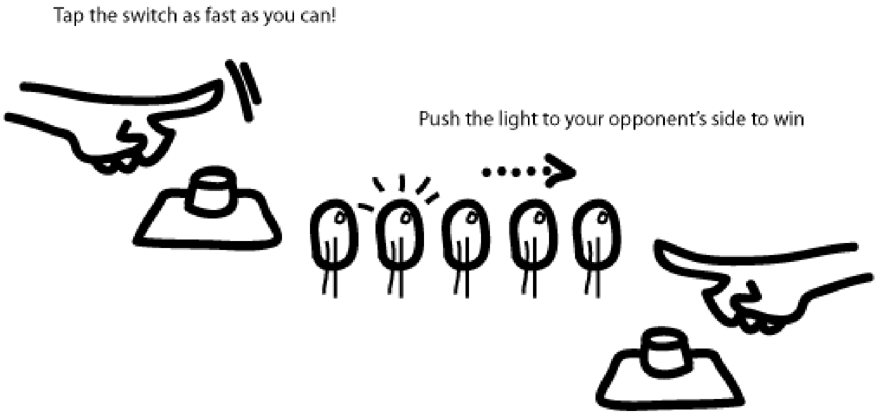

[Diagram 1]

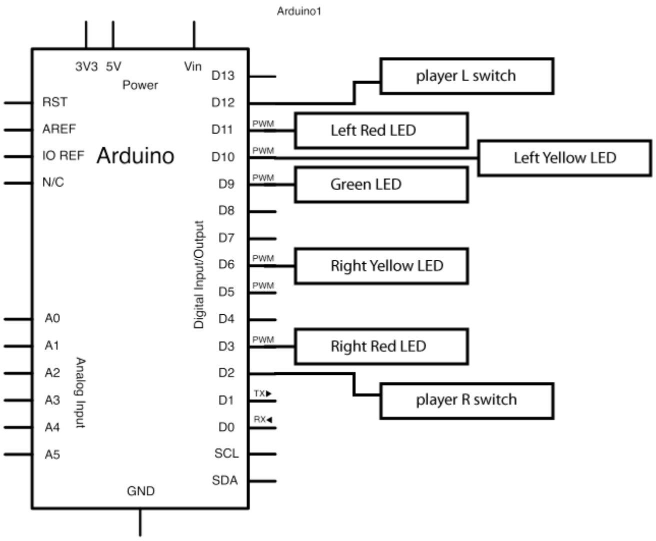

The Arduino Uno can be programmed using an IDE (integrated development environment), which is a software environment that comes when you buy the board. Let's start by connecting the Arduino with your computer.

1. Download the Arduino 1.0.3: http://www.arduino.cc/en/Main/Software
2. Run the setup
3. Connect the Arduino board via USB cable and run Arduino IDE
4. Go to menu > Tools > Board to check if "Arduino Uno" is checked. If not, check "Arduino Uno"
5. Go to menu > Tools > Serial Port > cu.usbxxxxxxxxx
6. Hit the reset button on the board. Check for indicator LED blinking
7. Go to menu > File > Examples > 01.Basics > Blink
8. Upload the sketch to the board by clicking the second icon (→) on the menu. Check if the LED 13 is blinking. Show your working Arduino setup to one of the teaching staffs. ______TA check

WRITING CODES FOR LEDS

For our game, we will need to control five LEDs. Let's start building the circuit in the **Diagram 1** by connecting five LEDs to the board.

1. Based on the circuit design you did in your homework, find <u>one</u> appropriate matching resistor each for a red, a yellow, and a green LED.
2. Connect the five LEDs to appropriate output pins according to the diagram. Also, think about the best layout for the LEDs on the breadboard, relating to the final design of the game (**Figure 1**).

3. In your Arduino IDE, open a new sketch by going to File > New. Using comment syntax (// or /* */), type in the title, author, date, and a brief description of the code.
4. Set your constants for LED output pins.
   For example: const int redLEDRPin = 2;
5. Let's write the setup function. This is the function that is going to be called first in the initialization step. We will set pin modes for all the output and input pins here. Also, if we need to read a physical value and set it to be an initial value for a variable, this is a good place to do it.

   Syntax:
   ```
   void setup() {
       // initialization
   }
   ```

6. In the body of the setup function, set correct pin mode for the output pins those are connected to LED circuits.
7. Next, we will write the loop function, which will be executed consecutively in loop as long as the Arduino system is turned on. This function will contain the main body/logic of the whole program.

   Syntax:
   ```
   void loop() {
       // statements
   }
   ```

8. To test your LED circuits, let's write a short program to turn on all the LEDs. Before we write all our code into the loop directly, remember that it is a good practice to write your program in modules with one module containing one block of tasks. One easy way to modularize is encapsulating a block of statements into a function. Outside of your setup and loop functions, let's declare a new function named `allLEDon`. This function will not take any parameter and return nothing. Expanding what you did in the homework, write codes that turn on all LEDs.
9. In your loop function, write a line to call the `allLEDon` function we just wrote.
10. On the top left of the sketch window, you will see a button with a check mark in it. Click it to verify your code. If you see "Done compiling" at the bottom of the window with Binary sketch size printed in the black background, your code doesn't have syntax bugs that the compiler can catch and your program has been successfully built!
11. Let's upload your program to the Arduino board. Click on the second button at the top left corner of the window. The button has an arrow icon on it. When the upload is done, the board's indicator LED will blink and your LEDs will light up. Show your working circuit to one of the teaching staffs. _____TA check

note: If the upload was successful but you don't see your LEDs turning on, then suspect that there is a problem with your circuit. Also, double check the code if it has the correct pin numbers.

12. Write another function `allLEDoff`, for turning all LEDs off. It should not take any parameters, and returns nothing.
13. Write another function `GameOver`, which will be used for indicating our game is over by blinking all LEDs.

    The function should
    `repeat 4 times:`
    call `allLEDon`
    give 300 msec of delay
    call `allLEDoff`
    give 300 msec of delay
    It should not take any parameters, and returns nothing.

    Hint: use the delay function and a FOR loop
14. Remove the `allLEDon` function call in the loop function and replace it with the `GameOver` function. Test the program by uploading it the board.

WRITING CODES FOR SWITCHES

Now, let's connect the pushbutton switches to the board.

1. Our switch will be sourced from the +5V pin of the Arduino board. Remember that the Arduino board's input pin has max sinking current of 40mA. If the switch is directly connect between the +5V and the ground and then shorted, it will destroy the Arduino. There are many techniques for avoiding this, however, we will use a resistor with adequately large value. If you want to limit the current through the switch circuit to be smaller than 0.5mA, which resistor should you use? Design a circuit and check it with one of the teaching staffs. _____TA check

2. Place your switches with the matching resistors on the breadboard. Again, think about the best layout for the elements with the final design of the game in mind.

3. In your `setup` function, set correct pin modes for the two switches.
4. Write a new function outside of the loop function, `ReadPinsAndCount`, which detects changes in the switch input pins to count how many times the each switch was clicked and save the counts to variables `countR` and `countL`. Hint: You have written the pseudocode of this function for one switch in the homework.
5. In order to test the switch circuits, we will run the `ReadPinsAndCount` function in the main loop and open the Serial connection to monitor actual values the variables. To open the Serial connection, write the following code into your setup function:
   `Serial.begin(9600);`
   This code will set the Arduino's serial data transmission rate to 9600 bit per sec.
6. Declare a new function `PrintValues,` which takes no parameters and returns nothing. Copy the following code into the body of the function to allow monitoring the live values for `countR` and `countL`.

```
Serial.print(countR);      // print live value of countR
Serial.print("\t");  // print tab

Serial.print(countL);      // print live value of countL
Serial.print("\n");  // print new line
```

7. Remove or comment out the `GameOver` function in the main loop. Call your `ReadPinsAndCount` function followed by `PrintValues` function. Compile and upload the program to the board.
8. While the program is running, click the Serial Monitor button at the top right corner of the Arduino IDE window. You will see the traced live values of `countR` and `countL` in the terminal. Click on your switches to check the switch circuits and the program you just created.

FINISHING UP GAME LOGIC CODES

Now we checked all of the circuit parts are working properly. We will need to write more modules and structure the existing modules in order to make this system into a playable game.

1. The logic of the game: At the beginning of the game, only the green LED in the middle will be on. While running a fixed number of loops, the number of the switch clicked will be counted and the light will be pushed to one step toward the side with less counts. The game will end when the light is pushed beyond the last LEDs.
2. We will use a regular FOR loop for the fastest data sampling states of the switch input pins. Because a short amount of time will pass for each step in a program is executed, we can adjust the loop completion time interval by changing the number of iterations of the FOR loop. Use `const int loopTime = 300` for this game project. Write a FOR loop that encapsulates `ReadPinsAndCount` and `PrintValues` in the main loop.

3. Each time after this FOR loop ends, we need to compare the values of `countR` and `countL` to determine which player won during that game interval (one iteration of the main loop). Write a new function `CompareCounts` outside of the main loop, based on the pseudocode you create in the homework. Call this function in the main loop after the FOR loop you wrote in part b. A new variable `LEDstate` with initial value of 2 (= green LED) will need to be declared to hold the information on which LED in the system will be on the following game interval.
4. LEDs are needed to be reset and controlled at the beginning of the each game interval to show the game progress correctly. Write codes for first turning all LEDs off and then turn on the correct LED according to the value of `LEDstate`.
5. Also the values of `countR` and `countL` are needed to be reset at the beginning of the each game interval. Write code for this step.
6. When does this game end? Write a code to determine when the game ends and call function `GameOver` when the condition is met.
7. Play a round of the game with your partner. Brag about the result to the teaching staff.
   _____TA check

Error analysis

Omitted for this module.

Discussion

When does a device become a game? Can you think of a game design applying techniques you learned so far in the course? Briefly describe your idea with sketches and diagrams.
