# Supplementary material for: A Biotic Game Design Project for Integrated Life Science and Engineering Education
Source: PLoS Biol. 2015 Mar 25;13(3):e1002110. doi: 10.1371/journal.pbio.1002110 (PMC4373802; doi:10.1371/journal.pbio.1002110)
Supplement: S15 Text — This module provides homework and lab instructions for the fifth electronics unit which involves pulse width modulation, motors, and transistors. (DOCX) [file pbio.1002110.s015.docx]

**BIOE 123 Module 6**

**Electronics 5: Arduino, PWM, Mosfetts and Motors**

**Lecture (30 min)**

Date

**Learning Goals**

- Learn about pulse width modulation (PWM) as a control technique
- Learn how to use a Mosfets to control high current drawing loads
- Learn how to drive basic DC motors and control their speed
- Learn how to program the arduino in Matlab
- Lean how to program a simple controller

**SUMMARY OF TOPICS** (actual lecture notes attached as separate document)

- What is pulse width modulation (PWM)?
  - Theory
  - Applications
  - Why it is useful
- Generating a PWM signal
  - Need some circuit/device to generate the signal and vary the duty cycle
  - Analog options (555, Schmidt trigger…)
  - Or, use a microcontroller (Arduino has this built in)
- Driving a load with PWM
  - Most PWM generated signals can’t directly power the load, use a Mosfet as a middle man
  - Mosfet ~ voltage controlled transistor, can source/sink lots of current
  - Pull up/pull down resistors and their importance
  - Demo of LED multi-color dimmer
- Driving inductive loads
  - Loads like motors/solenoids have lots of inductance: V = L(dI/dt)
  - Experience huge voltage spikes when switching rapidly
  - Use a shunting (kickback) diode to allow the voltage spike to dissipate
- Programing the Arduino in matlab
  - Loading the program in the IDE
  - Command list
  - Basic Event-Driven structure (while loop, events, services)

**Lab Intro (~15 min)**

Date

**SUMMARY OF TOPICS**

- Review of PWM signal generation and circuitry
- Proper circuit layout to avoid noise from high currents (avoiding ground loops)
- Review of Matlab environment
  - Commands
  - Event driven structure
  - Proportional controller (steady state error)
  - Bits

**Electronics 5: Arduino, PWM, MOSFETs and Motors**

**Problem Set 6**

Due: Date at the end of lab.

Text: Practical Electronics for Inventors, Scherz

Estimated reading time: 0.25 hr for Core reading

**Learning Goals**

- Learn about pulse width modulation (PWM) as a control technique
- Learn how to use a Mosfets to control high current drawing loads
- Lean how to program a simple controller

CORE READING

<http://en.wikipedia.org/wiki/Thermistor>

4.3.4 Metal oxide semiconductor field-effect transistors (Scherz)

13.1 DC continuous motors (Scherz)

13.2 Speed control of DC motors (Scherz)

13.3 Directional control of DC motors (Scherz)

PROBLEMS

Problem 1: You want to control the speed of a DC motor with PWM using a function generator and a MOSFET circuit. Draw the basic circuit that will allow you to control the speed of the motor. Assume you have a N-type power MOSFET, a dc motor, any passive components you might need (i.e. resistors, capacitors, diodes etc.) and a working function generator with variable duty cycle. Describe in words how you can change the motor speed continuously. Note, you will only drive the motor in one direction.

Problem 2: The motor you are using in problem 1 is a 12V dc motor with an armature resistance of 6Ω. What is the current drawn by the motor at stall? Look up the IRF520 N-channel MOSFET data sheet on coursework in the “datasheets” folder. Can safely use this MOSFET to control the motor?

Problem 3: Now, you want to make a circuit that will allow you to measure temperature. You have a thermistor with a negative temperature coefficient and a resistance of 10k at room temperature. Design a simple voltage divider that will produce an output voltage that varies with the thermistor temperature, and produces 2.5V at room temperature. If you want the output voltage to DECREASE when the thermistor temperature INCREASES, on which side of the divider should you place the thermistor?

Problem 4: For fun, you decide to create a simple feedback controller by combining your motor speed circuit from problem 1 with the temperature sensor in problem 3. You place a propeller on the output shaft of the motor, and have it blow air on the thermistor from problem 3. Thus, as the motor speeds up, the temperature of the thermistor drops and the voltage from your temperature sensing circuit will rise. If on the other hand the motor slows down, the thermistor will heat back up and the output voltage will drop. Think of a way to control the motor speed such that the thermistor is held at a constant target temperature and can resist disturbances like your lab partner heating the thermistor with his/her breath.

- Draw a schematic of the controller.
- Write a pseudo code program to implement this controller using the Arduino microcontroller you used in the last lab.

**Electronics 5: Arduino, PWM, MOSFETs and Motors**

**Laboratory Instructions**

Date

Location:

Personal (“collaborators”): Work in pairs. Supervision by instructor and TAs.

**Objective**

- Learn about pulse width modulation (PWM) as a control technique
- Learn how to use a MOSFETs to control high current drawing loads
- Learn how to drive basic DC motors and control their speed
- Learn how to program the arduino in Matlab
- Lean how to program a simple controller

**Background**

Today you will be building a simple controller with the arduino microcontroller and circuitry of your own design. Your task is to make a circuit with a motor driven cooling fan to keep the temperature of a thermistor nearly constant in the presence of external perturbations. Control circuitry is elemental to many real world systems, and it is useful to have some knowledge of how it works, and even how to make simple control systems. This lab simulates a real world experience where you would need to design and build some custom electronics for an experimental setup or testing equipment rapidly for prototyping purposes.

**Parts List/Materials**

- Breadboard (Jameco Electronics)
- Oscilloscope (Tektronix TDS 1012C-EDU)
- Function generator (GW Instek GFG-8020H)
- Jumper wire (Jameco Electronics)
- Various resistors, potentiometers (Jameco Electronics )
- Lab power supply (Mastech DC Power Supply HY3003D-3)
- LM324N op amps (Jameco Electronics)
- IRF520 N-channel mosfets (Jameco Electronics)
- N4935 Switching Diodes (Jameco Electronics)
- Motors (Jameco Electronics)
- 10K Thermistors (Jameco Electronics)
- Arduino Microcontroller

MATLAB Commands for Arduino

- **a = arduino('port')** where 'port' is the COM port to which the Arduino is connected to, e.g. 'COM5' or 'COM8' on Windows. This creates an object, a, that you can now apply the arduino commands to
- **a.pinMode (pin, ‘input’)** or **a.pinMode (pin, ‘output’)** to designate the pin mode
- **a.digitalRead(pin)**, reads the value of an analog pin. This is a 10 bit read so it varies between 0 and 1023.
- **a.analogRead(pin)**, reads the state of a digital pin, will be either 0 or 1
- **a.digitalWrite(pin,0)** or **a.digitalWrite(pin,1)** to set a digital pin high or low
- **a.analogWrite(pin, value)**, to set the value of an analog output pin. Value is an 8 bit number ranging from 0 to 255 that maps to a voltage of 0-5V. If the pin is one of the PWM designated pins, the output will be a PWM signal ranging from 0-5V with a duty cycle calculated by: 100*value/255, i.e. a 50% duty cycle is roughly 127
- Finally, use a.delete to delete the arduino object, (and free up the serialport) when the session is over. Its nice to have this at the end of your m-file

Other Useful Matlab Commands (type help “command” to see how to use them)

- Upon startup (i.e. at the beginning of your m-file) you should include the following commands to delete any lingering variables:
  - close all
  - clear
  - imtool close all
  - clc
- if, elseif, else
- for (needs an “end”)
- while (needs an “end”)
- plot
- save
- clc

**Experiments/Tasks**

motor

IRF520

1N4935

V^+^

GND

PWM (0 to V^+^)

G

D

S

10k

Figure 1: Motor Drive

Motor Speed Controller

- Construct the motor speed circuit shown in **Figure 1** using your breadboard and jumper wires. Use the alligator clips to connect the motor leads to the breadboard. Ensure that the kickback diode is in place and the cathode points to the high voltage power rail. Do all circuit construction without the power supply connected.
- Generate a PWM signal with the function generator. Use the oscilloscope to monitor the output of the generator, being sure to connect the grounds of the two probes to each other. Generate a square wave at 1kHz with a 50% duty cycle ranging from 0 to 5V. Use the cursors and/or quick measurements to verify the values in your input signal. Check to see that you can vary the duty cycle with the “duty” knob on the function generator.
- Connect the Function Generator output to the gate of the power mosfet in your circuit. Have a TA or instructor inspect your circuit before you power it up. You can use the 5V power supply to drive the motor circuit.
- Have a TA/Instructor verify that your circuit drives the motor, and that you can control the speed by changing the PWM duty cycle. _____TA check
- Clean up your circuit, trim resistors, and re-test to ensure that it works. Attach a small “fan” to the motor shaft by putting a strip of tape of the side similar to the tachometer setup you used in the past.

Thermistor temperature sensor

- Build a temperature sensor by using the 10k thermistor as one half of a voltage divider. This circuit was one of your homework problems, so build it as you designed it in your homework so that it has the proper voltage output at room temp, and changes in the proper direction when the thermistor is heated or cooled.
- Monitor the output voltage with the oscilloscope to make sure your sensor works. *Make sure the power rails for the temperature sensing circuit and motor circuit are separate and connect only at the power supply.

Connect the Arduino to Matlab

- Begin by connecting your arduino to Matlab. If you have already connected your arduino to your computer, all you need is the matlab support package. You can find this at: http://www.mathworks.com/matlabcentral/fileexchange/32374

After downloading these files, you need to upload the srv.pde file to the arduino board.

Upload the adiosrv.pde file to arduino

- This file acts as a “server” program to continuously communicate between matlab and the arduino. This communication takes place over the serial port. The downside is it slows the arduino down considerably. The upside is that you gain access to the versatility of math operations within matlab.
- From the Arduino IDE, go to File > Open, locate the file adiosrv.pde, (in the ArduinoIO/pde/adiosrv folder) and open it. If a dialog appears asking for the permission to create a sketck folder and move the file, press OK (this will create a adiosrv folder and move the adiosrv.pde file inside it).
- Connect the Arduino, make sure that the right board and serial port are selected in the IDE, (Tools/Board and Tool/Serial Port) then select File -> Upload to I/O Board and wait for the "Done Uploading" message. At this point the srv.pde file is uploaded and you can close the IDE, which is not needed anymore for the purpose of this package. Actually closing the IDE is suggested, so you can be sure that the serial connection to the arduino board is not taken by the IDE when matlab needs to use it.
- Open matlab, and run the “install_arduino” m file to make matlab identify the appropriate path to connect to the arduino. Now you should be all set to program the arduino from within matlab.

Write the motor speed control program

- Begin by creating an m-file to run your code. You can find help on this in the matlab help menu if needed. You should put all your code in here rather than writing it in the command window. However, note that you can cut and paste multiple lines from an m-file into the command window if you want to test them out. Also, pressing f5 will run the m-file automatically.
- Within the m-file, write an event driven program that monitors the voltage from your temperature sensor, the voltage from a target reference (use a potentiometer to make this voltage) and multiplies the error by a fixed gain to calculate a new PWM duty cycle value to drive the motor. Begin by setting up all the pin types and directions, declaring any global variables, and then add a while loop inside which you constantly measure the temperature sensor voltage and update the PWM duty cycle sent to the motor.
- Make the while loop conditional dependent on the state of a tactile switch on one of the digital inputs. This gives you an easy way to stop the infinite loop at any time by pressing the button.
- Play with your temperature sensor by squeezing it with your fingers to see how much the voltage fluctuates. Estimate a reasonable gain value to stay within a reasonable error when the motor is running at steady state.
- Use the oscilloscope to test your code on the arduino. The PWM duty cycle should vary as you heat the thermistor with your hand or the laser pointer. Have a TA/instructor check your PWM output and make sure it seems to work. _____TA check

Connect the arduino to your circuit and test

- Connect the PWM output from your arduino to the gate of the mosfet on your motor controller.
- MAKE SURE TO CONNECT ONE OF THE GROUND PINS ON THE ARDUINO TO THE GROUND ON YOUR BREADBOARD
- Check to ensure that your motor circuit and sensing circuits do not share a ground loop, but rather connect to the power supply with two separate cables each.
- Run your code, adjust the reference voltage such that the motor is off at room temperature, but begins to spin as you heat the thermistor. Adjust the gain so that the motor response is not too extreme.
- See if you can get the motor to run at a near steady state where the heat added to the thermistor is dissipated by the air the motor fans on it. Show the final result to one of the teaching staffs. _____TA check

**Summary results / error analysis:**

Not necessary for this module.

**Discussion:**

For your discussion think of a context in bioengineering where you could use the skills you practiced today. What properties would you want to improve, and how might you do that?
