# Supplementary material for: A Biotic Game Design Project for Integrated Life Science and Engineering Education
Source: PLoS Biol. 2015 Mar 25;13(3):e1002110. doi: 10.1371/journal.pbio.1002110 (PMC4373802; doi:10.1371/journal.pbio.1002110)
Supplement: S18 Text — (PDF) [file pbio.1002110.s018.pdf]

## Notes on Background Material:

The notes below compile information from many useful resources on optics. For an in depth discussion, please see the following:

Murphy, Douglas B. and Michael W. Davidson. "Fundamentals of Light Microscopy and Electronic Imaging," 2nd Edition, Wiley-Blackwell (2012) ISBN: 978-0-471-69214-0
http://www.wiley.com/WileyCDA/WileyTitle/productCd-047169214X.html

Hecht, Eugene. "Optics," 4th Edition, Addison Wesley, (2001). ISBN-10: 0805385665
http://www.amazon.com/Optics-4th-Edition-Eugene-Hecht/dp/0805385665

CVI Melles Griot Optics Guide. Melles Griot. 2002.

Pawley, James. "Handbook of Biological Confocal Microscopy." Springer (2010).
http://link.springer.com/book/10.1007%2F978-0-387-45524-2

Davidson, Michael W. and Mortimer Abramowitz. "Optical Microscopy Primer." http://www.olympusmicro.com/primer/microscopy.pdf

Truskey, George A., Fan Yuan, and David F. Katz. "Transport Phenomena in Biological Systems." Upper Saddle River NJ: Pearson/Prentice Hall (2004)

Nikon MicroscopyU. The Source for Microscopy Education: http://www.microscopyu.com/

Olympus Microscopy Resource Center: http://www.olympusmicro.com/

Molecular Expressions Optical Microscopy Primer: http://micro.magnet.fsu.edu/primer/index.html

**Copyright statement:** All images and figures included here have been generated by the BioE 123 teaching team. Creative Commons images are indicated by 'CC.'

**Acknowledgement:** AKD gratefully acknowledges Professor Daniel Fletcher and Neil Switz, whose optics course at UC Berkeley inspired some of our lab modules.

**Background Material & Problem Set for Optics 1: Optics basics, focal lengths of lenses, finite imaging, exploring the thin lens equation, building an infinity-corrected optical system**

**We expect you to know the following after completing this reading and problem set:**
-explain how properties of light change when it travels through media of different refractive index
-apply Snell's law to find wavelength and direction of light at interfaces between different media
-explain image formation and how lenses aid in forming images
-explain why spherical aberrations result
-perform ray tracing through optical systems
-find magnification and focal length of a lens using the Thin Lens Equation
-identify finite and infinite imaging systems and the benefits of infinite imaging

**What is light?**
Fast review of physics here: Light has characteristics of particles and waves, but we are more concerned with the wave-like properties of light when we think about optics. Light doesn't stop when it reaches ends of media (liquids, gasses, solids) but reflects, diffracts, and refracts.

*Reflection: "bouncing off an obstacle"*
The angle at which light waves approach a flat reflecting surface is equal to the angle at which the light waves leave the surface (see picture on right, Law of Reflection). The reflection coefficient describes how much of light is reflected from an interface and depends on indices of refraction (see below) of the two media (above the reflective surface and the reflective surface itself). The formula is $R = [(n_1 - n_2) / (n_1+n_2)]^2$. Light transmitted by the material is whatever is left ($T = 1-R$).

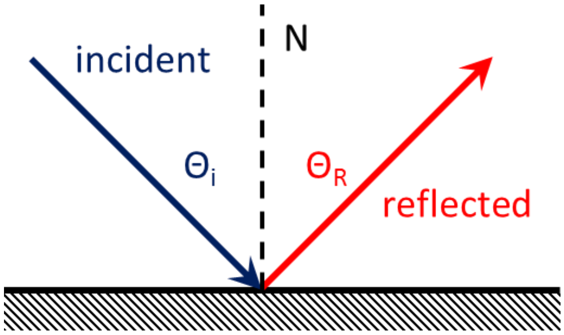


*Refraction: "light bends when crossing boundaries between different media"*
Light passing from one medium to another "bends" because of changes in the refractive index of the media. A common example is a pencil in a glass of water will appear "broken" depending on the angle at which you observe it because light travels differently through the water as it does through the air. The direction of the bending of light depends on how much the light's speed changes when going through the different

media. Snell's law (which we will discuss below) is a formula which describes the angle which light will take when passing through different media depending on the index of refraction of the media. The index of refraction correlates to the density of the material, see list below.

| **Material** | **Index of Refraction (n)** | **Wavelength at which it was measured (λ, nm)** |
|---|---|---|
| Vacuum | 1 | By definition |
| Water | 1.333 | 589.29 |
| Human eye lens | 1.386 – 1.406 | |
| Crown glass | 1.50 – 1.54 | |
| Immersion oil | 1.515 | |
| Silicone oil | 1.52045 | |
| Diamond | 2.419 | 589.29 |
| Silicon | 3.96 | 590 |

*Diffraction: "light waves change direction when passing through an opening or around an obstacle"*

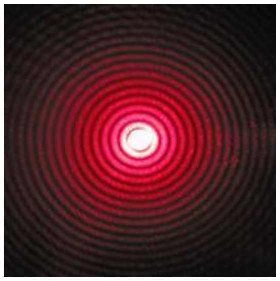

If light cannot penetrate an object, there will be a shadow behind the object. But the edges of the shadow are fuzzy because light diffracts and changes directions around the object and that's most noticeable at the object edges. When the obstacle (or opening) is small, we can note diffraction patterns from how the light waves constructively and destructively interfere in the wake of the object. See figure at right showing the diffraction pattern of a red laser beam after it passes through a small hole (CC image from: http://en.wikipedia.org/wiki/Diffraction#mediaviewer/File:Laser_Interference.JPG). We will actually measure diffraction patterns of microbeads in one of the labs in this class as these particles can be approximated as "point sources" and produce Airy disks diffraction patterns.

Also remember general properties of waves such as frequency, amplitude, and wavelength. All of these properties come up when we talk about light which illuminates our systems or carries information about our images.
A good primer:
http://www.studyphysics.ca/newnotes/20/unit03_mechanicalwaves/chp141516_waves/lesson44.htm

| **Parameter** | **Units** | **Symbol** | **Formula** |
|---|---|---|---|
| Period duration | Seconds | $\tau$ | $\tau = 1/f$ |
| Frequency | Hertz | $f$ | $f = 1/\tau$ |
| Wavelength | Meters | $\lambda$ | $\lambda = v/f$ |
| Velocity of light | m/s | $v$ | $v = c/n$ where c is the speed of light in a vacuum (~3E8 m/s) and n is the index of refraction of the medium ($n \geq 1$ for all practical cases) |

Based on this relationship of frequency and wavelength, different wavelengths of visible light have different frequencies (and colors).

| Color | Wavelength (nm) | | Frequency (THz) | |
|---|---|---|---|---|
| Red | 700 | 635 | 430 | 480 |
| Orange | 635 | 590 | 480 | 510 |
| Yellow | 590 | 560 | 510 | 540 |
| Green | 560 | 490 | 540 | 610 |
| Blue | 490 | 450 | 610 | 670 |
| Violet | 450 | 400 | 670 | 750 |

**Light wavelength and direction changes when going through different media**
An important point is that when light travels from one medium like air to another (like a lens, made of glass) **the frequency of the light does not change.** Light traveling in the air causes electrons to oscillate in the glass at the same frequency when it hits the glass. Yet light “slows down” in denser materials as the density of material will cause index of refraction to increase, see “velocity of light” formula above ($v = c/n$).

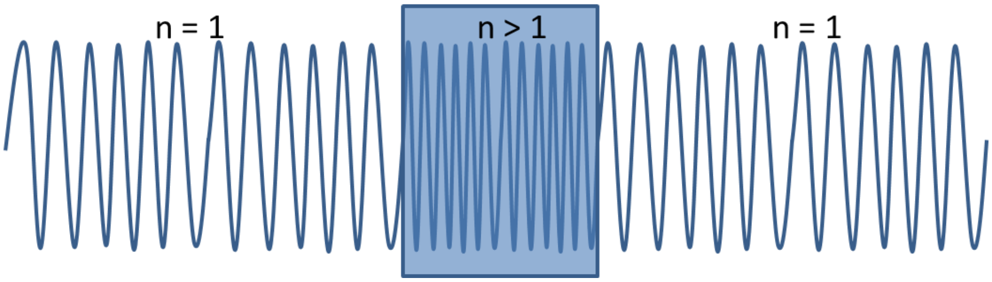


So if frequency is conserved and velocity changes, light must change wavelength when it hits different media (according to $\lambda = v/f$).

Let’s take an example of light going from vacuum to glass. n for vacuum = 1 (by definition), velocity of light in the vacuum is: $v_{vacuum} = c/n_{air} = c/1 = c$
and its wavelength is: $\lambda_{vacuum} = v_{vacuum}/f = c/f$
so $f = c/\lambda_{vacuum}$

$v_{glass} = c/n_{glass}$
$\lambda_{glass} = v_{glass} /f = (c/n_{glass})/f$
and $f = c/ \lambda_{vacuum}$
$\lambda_{glass} = (c/n_{glass})/ (c/ \lambda_{vacuum}) = \lambda_{vacuum}/ n_{glass}$
and so, n for glass = 1.5
$\lambda_{glass} = \lambda_{vacuum}/ 1.5$
thus the wavelength in glass is 2/3 the wavelength of the light in vacuum. This matters because the wavelength of light also limits the smallest objects we are able to resolve (or find in focus) with a light microscope. We will go over this relationship later, but shorter wavelengths lead to higher resolution.

Thus, according to the formula above we can increase resolution by using either shorter wavelengths of light that are coming into the glass ($\lambda_{vacuum}$) or increase the refractive index of the glass material ($n_{glass}$) to make $\lambda_{glass}$ smaller (and thus achieve higher resolution). This is the reason some microscope objectives use oil (n = 1.515 for immersion oil) to increase the n and thus decrease the wavelength of light that penetrates through the oil material, thereby increasing resolution.

**Snell's law**

This formula describes the relationship between angles of incidence (deviation from "head on" angle, 90°) and refraction of light waves as they pass through the boundary of two different media.

$\sin(\Theta_1)/ \sin(\Theta_2) = n_2/n_1$

We will work through an example with air and glass. Let $n_{air} = 1$ (approximate vacuum) and $n_{glass} = 1.5$, matching the example above. The frequencies of the waves must be the same so the wave crests must match up at the interface (see Δx in the picture on the right).

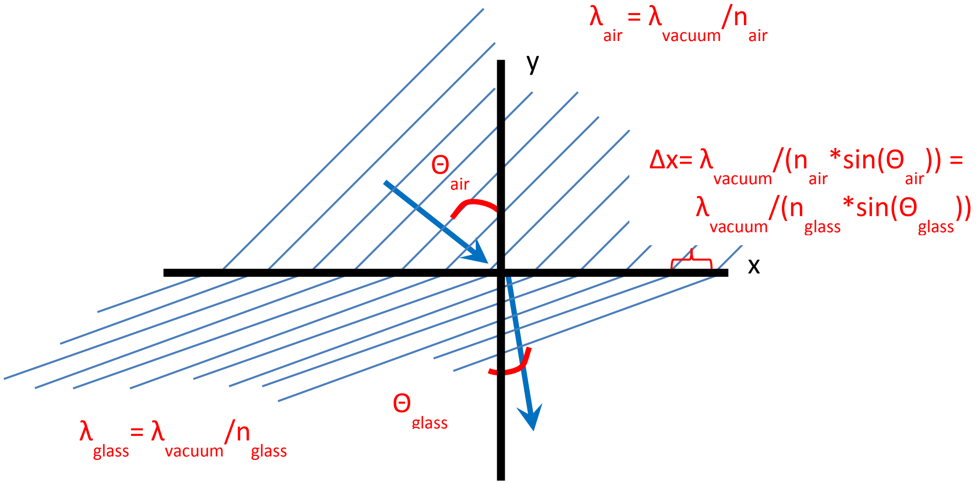


The wavelength for the glass side is shorter (waves are closer together) because $n_{glass} > n_{air}$. At the interface, since the wavelengths differ between light penetrating the two media, the wave bends and wavefronts have to be at different angles to conserve the

frequency of the wave. This is what leads to Snell's law dictating the relationship between refractive index and angles of incidence of the two materials.

$n_{air}*\sin(\Theta_{air}) = n_{glass}*\sin(\Theta_{glass})$

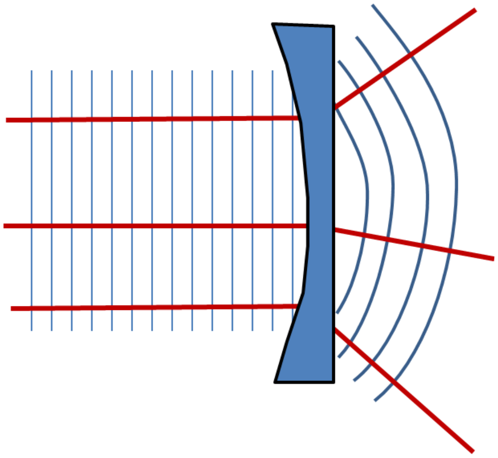

In this example, we also use vectors to represent the direction of light wavefronts (see red vectors, wavefronts are in blue in diagram at right). These are "light rays." The bending of light at interfaces between materials results in shifting in the direction of wavefronts and thus it is easy to use vectors to show where the peaks of the wavefronts will be headed. Rays are perpendicular to wavefronts.

**What is an image?**

Before we get into how lenses bend light, let's review that **an image is a light distribution re-created to mimic what is going on at the sample.** Images can be scaled up or down by magnification.

We cannot just put a piece of film or light detector right in front of the sample to get an image. Okay, maybe we can if the sample is self-illuminated (glowing), but we will have to be super-close. Try holding a piece of paper to your computer screen and get an image of the screen on your paper. You have to be pretty close! That's because light from the sample will not be focused in any way to re-create its distribution from the sample at the image plane. When light propagates without any shaping, it diffracts, refracts, and gets reflected instead of forming a crisp image of our sample.

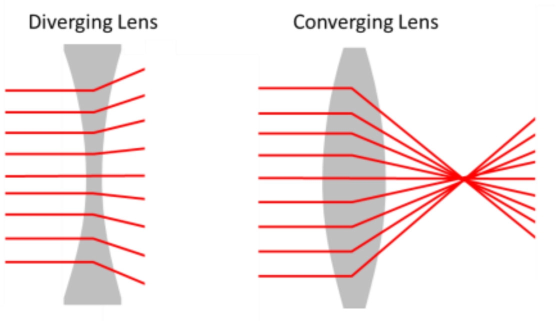


**What a lens does to light waves**

Now that we have reviewed what can happen between different media, surfaces, and light waves, we can think about what lenses do to light waves. We want lenses to make an image clear and allow for scaling (magnification) of our images. Lenses can be glass or other transparent material which either converge or diverge parallel incident rays (see diagram of converging and diverging lens at right).

There are different lens shapes which converge light in various ways (CC image at right from http://en.wikipedia.org/wiki/Lens_%28optics%29#mediaviewer/File:Lenses_en.svg). For this class, we will primarily be using plano-convex lenses.

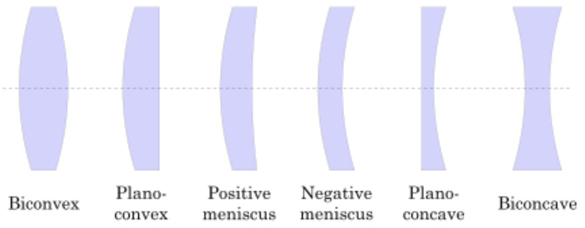


Lenses refract light according to Snell's law due to the changes between light traveling in the air versus the glass material of the lens. Due to the higher angles at the rim of lenses versus the middle, lenses bend the light rays on the outermost corners the most to converge at the focal point, an intrinsic property of the lens determined by its radius of curvature. You can see when examining the lenses you'll be using in lab. Plano convex lenses with lower focal length (25.4 mm) are more rounded than the ones with longer focal lengths (125 mm, 200 mm, 300 mm, and so on).

A positive converging lens focuses light from "infinity" to its focal point in the following way (CC image at right from http://en.wikipedia.org/wiki/Lens_%28optics%29#mediaviewer/File:Lens1.svg). We will discuss how to derive ray tracings to create figures such as this in the next section. A diverging lens will instead bend light at the focal point to expand on the other side.

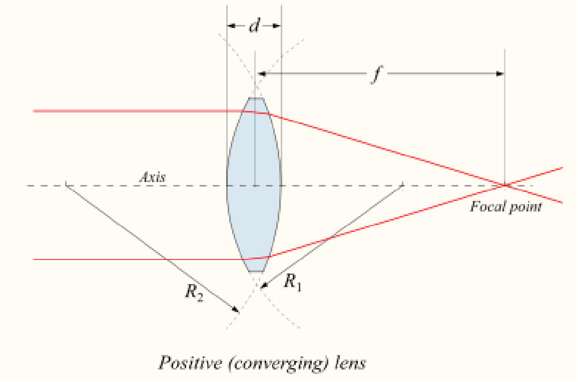


**Spherical Aberrations**

Note that many lens shapes are spherical because these are the easiest surfaces to make. In order to make a sphere, two surfaces are ground together in rotation and both naturally become spherical. Spherical surfaces are "not thick enough" at the edges compared to the ideal curves pictured above and so light "bends back" prematurely before the focal point when focused from the edges of the lens. This is known as a spherical aberration. Spherical aberrations become more pronounced as the radius of curvature of the lens increases (i.e., you will notice more spherical aberrations in plano-convex lenses which have a shorter focal length as they have a higher curvature).

Achromat lenses are corrected for spherical aberrations and chromatic aberrations (which originate from the fact that different wavelengths of light travel through materials at different speeds). Achromats provide an even paraxial image plane at the focal point as they reduce spherical aberrations. So why are we using plano-convex lenses in lab?

They are much less expensive for rapid prototyping of optical systems and they teach you the importance of watching out for spherical aberrations, especially with high-curvature plano-convex lenses!

**Ray Tracing Rules**

This is the part you've been waiting for! In this section we learn how to follow light rays as they travel through optical systems and thus predict where images will be formed! All of the rules here are derived from Snell's law on light bending through media of different indices of refraction. The middle of the optical axis is called the "principal axis."

I will use different colors for the light rays (red, blue), but keep in mind that this does not necessarily correspond to the wavelength of the light, I am just trying to make the diagrams easier to understand. Different wavelengths of light will focus differently through lenses stemming from the equations we mentioned at the beginning of the document ($\lambda = v/f$ , $v = c/n$).

**Rule 1)** Light rays passing through the center of the lens are not affected by the lens (if everything is aligned). The light may be attenuated (by reflection of the lens material), but in ray tracing we only consider the direction, not intensity, of light rays.

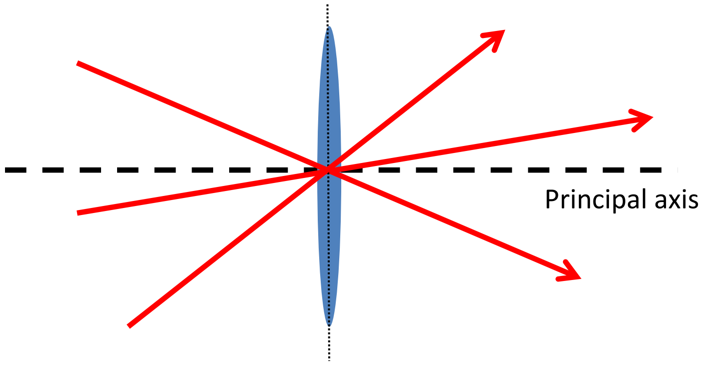


**Rule 2)** Rays which run parallel to the principal axis before hitting the lens converge onto the focal point on the other side of the lens.

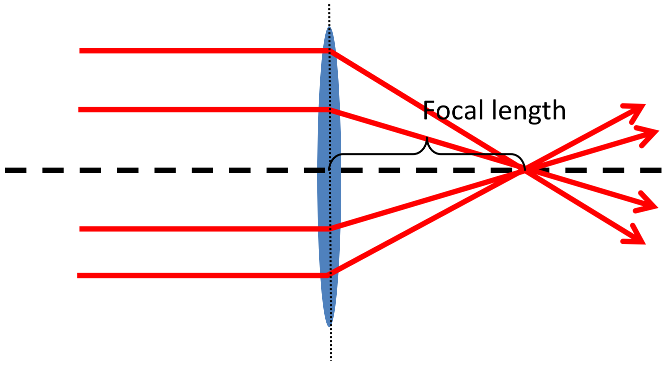

**Rule 3)** Rules 1 and 2 are completely reversible. Both sides of the lens follow rules 1 and 2. This means that rays which cross at the focal length, end up parallel on the other side of the lens.

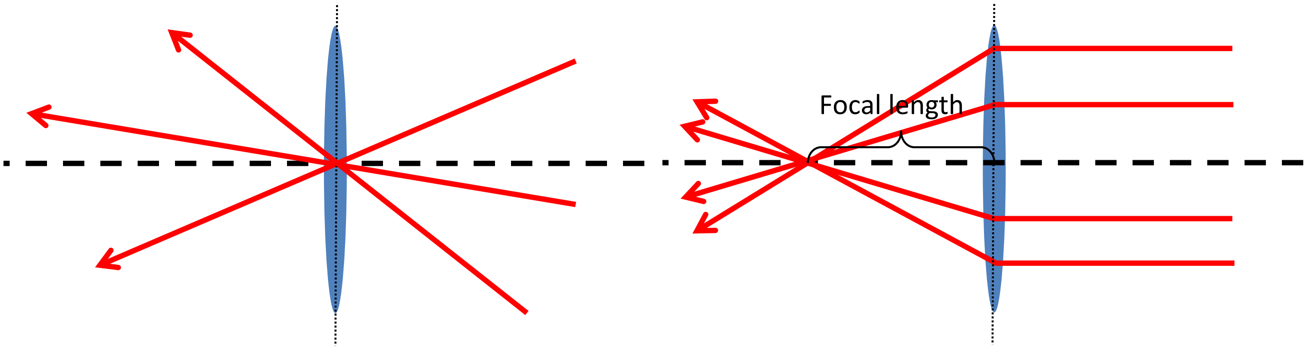


When we ray trace, we approximate light sources (either from illumination source like an LED, halogen lamp, or light coming from a sample) as points. These “point sources of light” always start diverging after being emitted. See example below for how 3 points on a sample are traced through an objective lens and focused into the back focal plane of the objective.

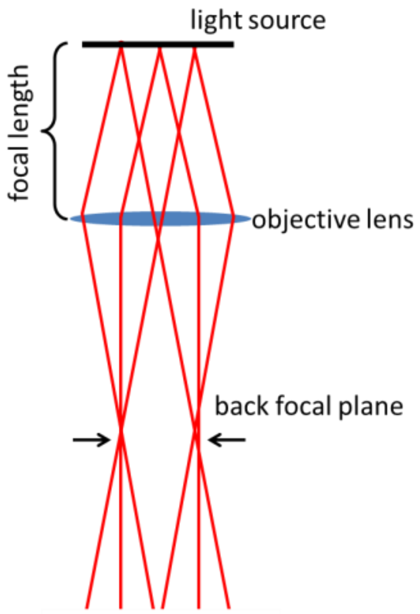


More on ray tracing rules here:
[http://scripts.mit.edu/~20.309/wiki/index.php?title=Geometrical_optics_and_ray_tracing](http://scripts.mit.edu/~20.309/wiki/index.php?title=Geometrical_optics_and_ray_tracing)

**Thin Lens Equation:**

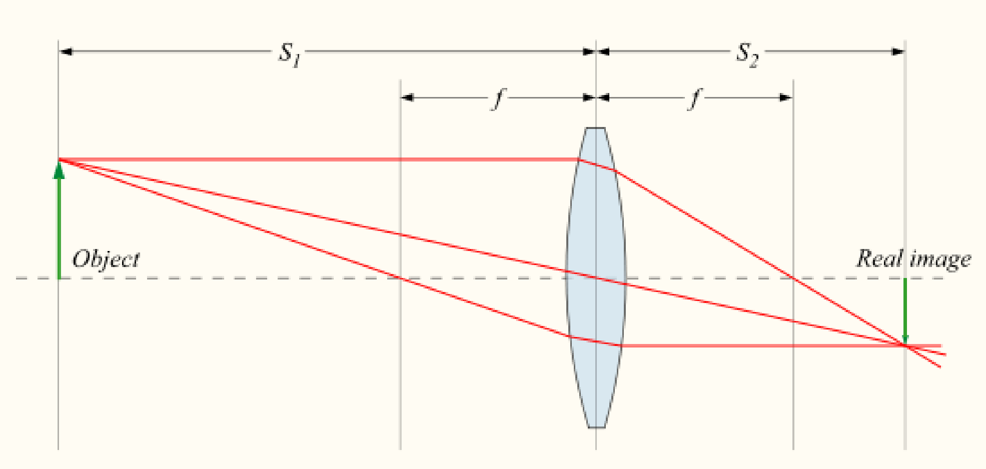


A lens will form an image of an object placed on one side of it if the distance to the image and object obey the following:

$$\frac{1}{f} = \frac{1}{s_1} + \frac{1}{s_2}$$

f in this case is the focal length of the lens (not frequency of light, as before). $S_1$ is the distance to the object and $S_2$ is the distance to the image (CC image at right from http://en.wikipedia.org/wiki/Lens_%28optics%29#mediaviewer/File:Lens3.svg). A real image of the object is upside down compared to the object. The formula for magnification reflects this with a minus sign:

Magnification = $-S_2/S_1 = -S_{image}/S_{object}$

We will be using lenses to form images of objects which are very far away. So far away we can assume that distance is infinity. An interesting thing happens when we make the assumption that $S_1$ ($S_{object}$) is infinity:

$$\frac{1}{f} = \frac{1}{\infty} + \frac{1}{s_2} = \frac{1}{s_2}$$

And thus, $f = s_2 = s_{image}$

This means that if we want to image something "infinitely" far away, we need to put the image at the focal length of the lens. To capture the image, we could put a photodetector there, like film or a camera chip.

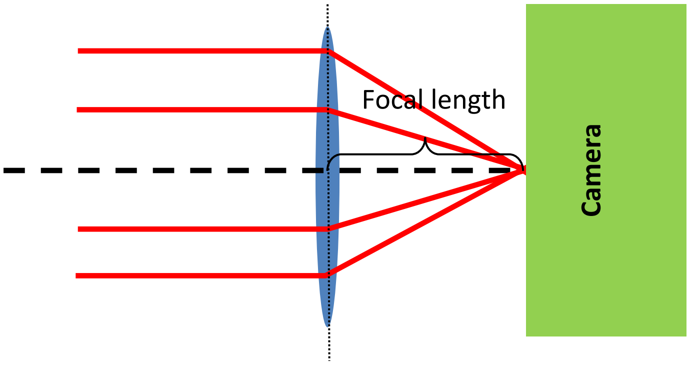


A lens in this configuration with the camera focused to infinity is called a tube lens**.**

You might be wondering why I didn't draw your eye at the position of the camera as this is the first "camera" you typically think of. It is because your eye can already focus to "infinity." The lens of your eye is an adjustable tube lens (via ciliary muscles), your iris is a diaphragm which lets more or less light in (depending on illumination conditions), and the back of your eye (the retina) is the equivalent of photography film or the CCD. The accommodation reflect of the eye is responsible for activating the ciliary muscles to focus near and distant objects by changing the shape of the lens. The retina is where images are projected from the world around you and transmitted to electrical signals in the brain for processing via the photoreceptor cells of the retina and associated neurons. More on this here: http://hyperphysics.phy-astr.gsu.edu/hbase/vision/accom.html

**The Finite Imaging System**

If we think about the thin lens equation we learned about above, how can we get magnification out of a system where we know that objects are translated to real images by lenses? If we put two lenses in a row could we 1.) magnify our image and 2.) make it 'upright'? It turns out that by calculating where real images appear in our system, we can! See the example below.

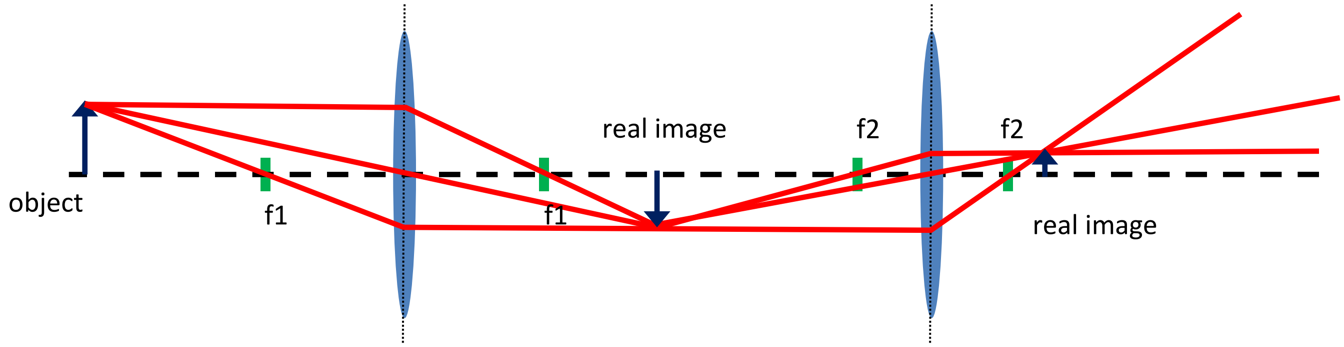


In the picture above, we traced points of light coming from our object to form the first real image and then the second real image. We can see that the object first got de-magnified and inverted by the first lens. Then the image was further de-magnified and inverted again to be upright by the second lens. The focal length of the lenses and position of the object relative to the focal length determined how much the image was magnified (or in our case, de-magnified). This finite imaging system is interesting because it provides us with a real image at the end which is "upright" relative to our sample object. Yet it is inconvenient.

Think about this: the distances between the object and real images relative to the lenses cannot be changed. We are limited to how we can setup our system. We have to

broadcast the second real image a specific distance from the object (no more and no less). If we change one distance in the system (let's say the position of the 2nd lens), both the magnification and position of the second real image change (example below).

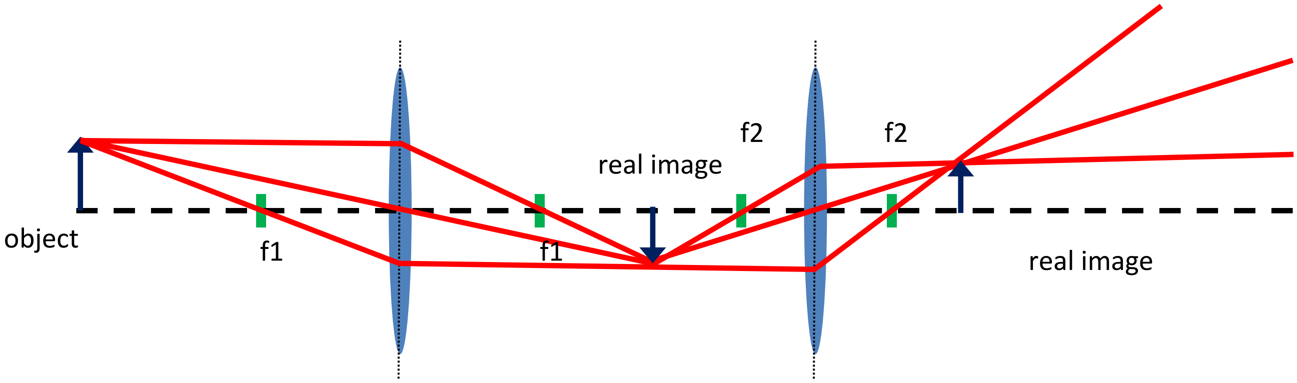


We can clearly see that according to the ray tracing rules, if we displace the 2nd lens, we will get a different magnification and position of the second real image, which is not practical for most optics setups. We want to have "space" in our system to put other components (like filters and mirrors). This is where 'infinity space' comes in very handy!

**The Infinite Imaging System (basic microscope!)**

What happens now if we can put the object at the focal point of the first lens in the 'finite imaging' example above? Take a moment to think about how the light rays would look on the other side of the first lens and only then look at the figure below. Remember to use the thin lens formula.

$$\frac{1}{f} = \frac{1}{\infty} + \frac{1}{s_2} = \frac{1}{s_2}$$

And thus, $f = s_2 = s_{image}$

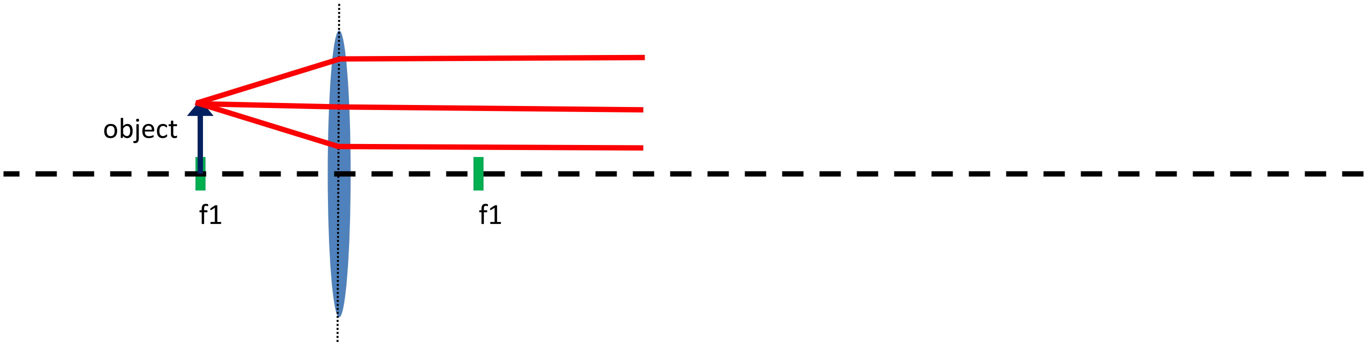


We have projected the image of our object to infinity. This means we are not able to focus any part of the image in the space to the right of the first lens. But by using the second lens in a similar way, we can now re-focus this image projected to infinity onto the focal length of the second lens (see below). The ratio of the focal lengths will then

determine the magnification of the real image seen to the right (from thin lens equation discussion).

We note that magnification is given by M = -f2/f1
Since f2 is smaller than f1, we see a de-magnification of our object in the real image.

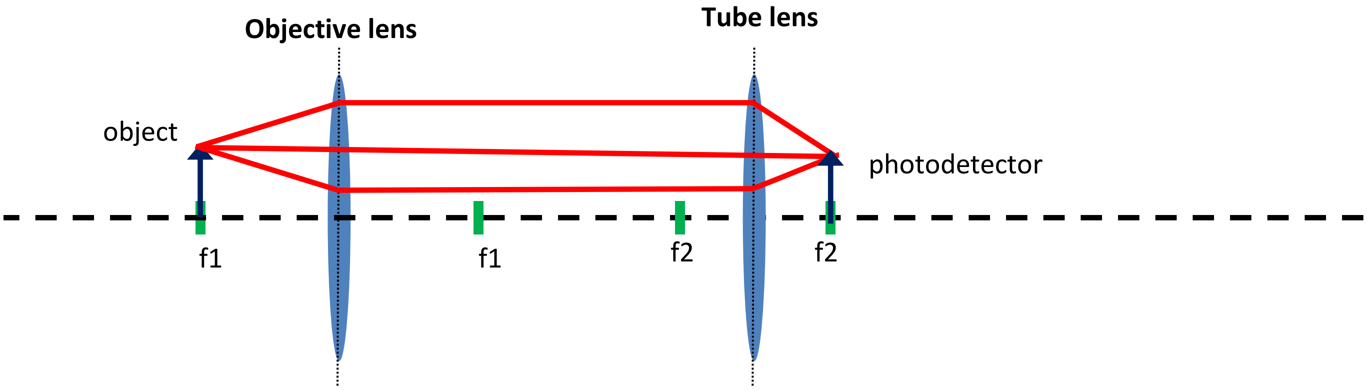


What is great about this system is that the middle is now 'infinity space.' All objects placed at the focal point of the first lens (f1), will be projected in parallel rays until the other lens (with focal length f2) converges them onto its focal point, which determines the magnification. We have thus made magnification and distance from the object to real image independent! Of course, it is now crucial to put objects exactly at the focal point of the first lens and to look for real images exactly at the focal point of the second lens.

In infinity systems, the lens closest to the object is called the "objective" and the lens closest to the camera (or photodetector) is called the "tube lens." (see labels in the diagram above)
$M = -f_{\text{tube lens}}/f_{\text{objective lens}}$

**Problem Set Questions:**

1.) Using a figure similar to the explanation of Snell's law, draw a figure for how light waves reflected off a mirror surface will be equal and opposite angles from the incoming light rays.

2.) Given two materials of unknown refractive index ($n_1$ and $n_2$), how will you determine which refractive index is higher? Assume that you can use a fixed laser light source and that you also have a spectrophotometer to measure the wavelength and frequency of light in both media ($n_1$ and $n_2$). Limit your answer to 200 words.

3.) Light goes from a thick (high refractive index) to thin (low refractive index) material. Which direction does it bend? You can draw a diagram or form your argument around angles of incidence. You can use some arbitrary refractive index values and starting directions for the light rays to help you.

4.) You observe magnification of an image by 10 times. You know that your system is an infinite imaging system and your tube lens has a focal length of 300 mm. What is the focal length of the objective?

5.) Using ray tracing, show the location of the image formed when an object is placed exactly a distance 2*f (two times the focal length) from the center of a thin lens.

6.) Again using ray tracing, show the location of the image formed when an object is placed >2*f (greater than two times the focal length) from the center of a thin lens.

7.) Based on the above two problems, where do you predict an object which is placed somewhere between 2*f and f focus on the other side of the lens? Draw the ray tracing diagram to find out. HINT: you may not have to even draw anything new if you think about the symmetry of a lens!

8.) Is it possible to focus an object using a lens when the object is closer to the lens than the focal point? Draw a ray tracing diagram.

**Background Material & Problem Set for Optics 2: Photodetectors, cameras, and light sources**

**We expect you to know the following after completing this reading and problem set:**
-explain how images are captured by photodetectors
-explain how a Charged Coupled Detector (CCD) captures photons into electrical signals
-discuss limitations of cameras and how they can impact image resolution
-logically match a camera/detector to a specific application
-discuss types of light sources for imaging and what it means for a light source to be collimated

**What is a digital image?**

We learned about image formation in the previous lab and background material. Now we delve into how we can store images into digital media. A digital image is made by taking measurements of how many light particles are hitting the surface of a material (photon flux in a given area). The **photoelectric effect** is what allows for this measurement (CC image at right from: http://en.wikipedia.org/wiki/Pair_production#mediaviewer/File:Photoelectric_effect.svg). Due to the photoelectric effect, some materials emit electrons when they absorb energy from light. In essence, all digital images are matrices of numbers where the rows and columns represent each part of the image (X and Y coordinates) and the numbers within each cell of the matrix represent the number of photons captured at that point (light intensity).

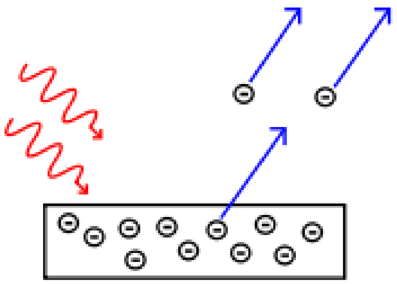

Your eye is making the same measurement and translating it to your brain through photoreceptor cells of the retina (rods, cones) which get activated and pass signals to neurons in the optic nerve. A great resource about the human eye: http://hubel.med.harvard.edu/book/b8.htm

**How do digital detectors convert photons to digital numbers which can then be displayed on your screen?**

Photons excite electrons and cause them to be emitted in the detector material. The release of electrons then results in a change the electric current through the material. This can be read off as a voltage and translates to a digital number (see below for how

light intensity correlates to a range of numbers) based on how many photons were present while the detector was recording the signal.

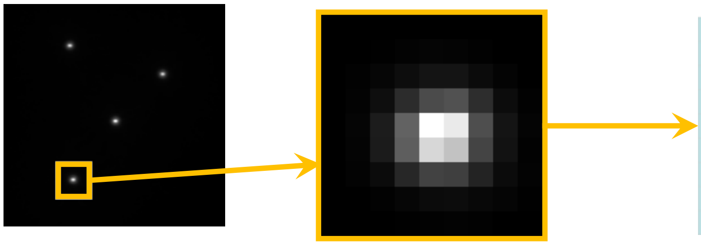

| | | | | | | | | |
|---|---|---|---|---|---|---|---|---|
| 0 | 0 | 0 | 0 | 0 | 0 | 0 | 0 | 0 |
| 0 | 0 | 1 | 3 | 5 | 4 | 2 | 0 | 0 |
| 0 | 2 | 6 | 13 | 20 | 20 | 11 | 4 | 0 |
| 0 | 3 | 14 | 44 | 75 | 81 | 45 | 12 | 2 |
| 0 | 5 | 28 | 98 | 255 | 234 | 78 | 20 | 4 |
| 0 | 4 | 27 | 94 | 215 | 194 | 68 | 18 | 2 |
| 0 | 3 | 11 | 39 | 66 | 63 | 35 | 11 | 3 |
| 0 | 0 | 2 | 6 | 11 | 12 | 8 | 5 | 1 |
| 0 | 0 | 0 | 1 | 2 | 3 | 2 | 0 | 0 |

Digital detectors can be “single point” or “multiple point” (cameras). Filters/coatings on single and multiple point detectors determine which specific set of wavelengths they record. In all detectors, quantum efficiency (QE) is important as a measure of how sensitive the system is (what fraction of photons present are detected). One can quickly move around a single point detector to get information from many points in a sample, but since it takes time to get the signal at each point and move onto the next one there is a lag between capturing signals.

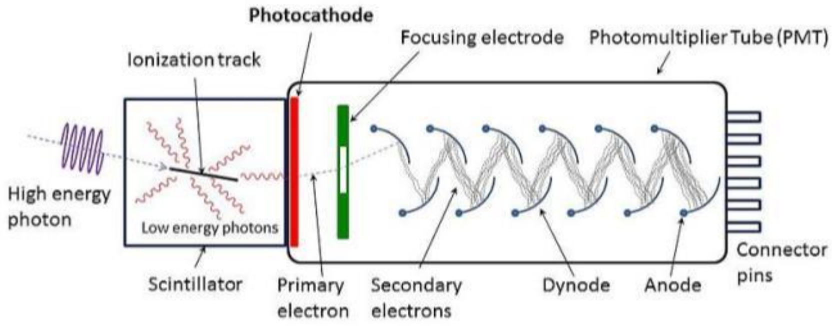


Single point detectors do not capture the lateral (XY) distribution of signal, only the overall intensity at a single point. The huge advantage is speed. Many systems use photomultiplier tubes (PMTs) to increase the amount of signal from a source if the number of photons is below the threshold of the detector (see CC image at right from: http://en.wikipedia.org/wiki/Photomultiplier#mediaviewer/File:PhotoMultiplierTubeAndScintillator.jpg). Single point detectors with PMTs are common in spectrophotometers – machines used to apply a specific wavelength of light through a sample to determine how much the sample absorbs/emits light. These platforms are useful for quickly determining concentrations of reactants, particles, and cells.

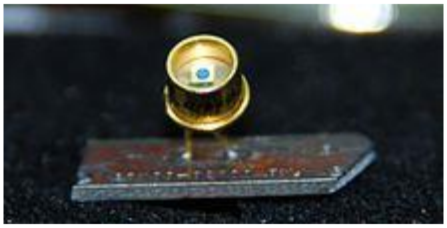

On the left is a picture of an avalanche photo diode (APD), an element which converts light to electricity through the photoelectric effect and contains a built-in amplification system to increase the signal from the source (first stage gain) (Cc image from: http://en.wikipedia.org/wiki/Avalanche_photodiode#mediaviewer/File:Avalanche_photodiode.JPG). Photons are absorbed by the silicon oxide layer and signal is amplified

through avalanche multiplication (as high reverse voltage contains 'depletion regions' which causes electrons to amplify the signal from a photon source through impact ionization).

Multiple point detectors can be thought of as arrays of single point detectors which capture the signal from each point across the sample at the same time. Each detector on the camera chip corresponds to a **pixel, or a small sampled part of the representation of the original image**. The more pixels a chip has, the greater the sampling of the image (and thus greater detail and resolution). The individual detector elements come in 2 primary varieties, CCD and CMOS, which we will discuss briefly. **The Logitech webcam you will be using in the lab is a CCD.**

**Comparison of photodetector types:**

| | **Complementary Metal Oxide Semiconductor (CMOS)** | **Charged Coupled Devices (CCD)** | **Nonimaging single point (with PMTs)** |
|---|---|---|---|
| **Method of Operation** | Each pixel has an amplifier and transfers voltage | Single read-out amplifier increases the signal of each pixel and pixels transfer charge | See above PMT description. |
| **Pro** | Fast | Slow | Fast, low noise |
| **Con** | Noisy (high noise versus signal, low Signal-to-Noise ratio) | Precise | moderate quantum efficiency, single point (unless detector moves) |

We won't discuss the specifics of how photoelectrons are relayed on each type of chip. Check out this reference if you are interested: http://thelivingimage.hamamatsu.com/resources/ccd-vs-em-ccd-vs-cmos/

**What are some useful specs to know when picking out a camera?**
The answer is it depends on what you are imaging and how much you care about speed versus precision

Pixel size and number: The size of the pixels has to be smaller than the features of your image to resolve them. If your pixels are too big and your signal from a feature on the image gets averaged over the pixel (since each pixel only records 1 intensity value), then you won't know that the feature is there! The more pixels a chip has, the more you can sample your image.

Quantum Efficiency: fraction of photons hitting the sensor which will be converted to electrons

Full well depth/capacity (FWC): total number of photons that can be recorded per pixel. This sets the “max” of your pixel in how much light intensity it can handle. Anything above will flat line the sensor.

Read noise: baseline for the noise the pixels read when no signal is present.

Dynamic range: the number of intensity levels one can distinguish is a function of the full well depth and readout noise (dynamic range = FWC/readout noise). The dynamic range of the human eye is about 100.

Comparison of dynamic range and full well capacity for some cameras: [http://www.andor.com/learning-academy/dynamic-range-and-full-well-capacity-a-definition-of-ccd-dynamic-range](http://www.andor.com/learning-academy/dynamic-range-and-full-well-capacity-a-definition-of-ccd-dynamic-range)

Readout time: how long it takes for the whole chip array to be converted to a digital image. Keep in mind it also takes time for the computer to save your image. This becomes an issue with large, high resolution (large number of pixels) images.

**Limitations of Photodetectors:**

Because the image is digitalized by the photodetector into a matrix of numbers, the precision of the measurement of photon flux by a photodetector depends on how many bits are recorded overall for the image.

| # of bits | Range of gray levels ($2^{(\# \text{ of bits})}$) | | |
|---|---|---|---|
| 1 | 2 | 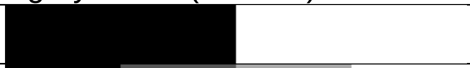 | Binary Image |
| 2 | 4 | 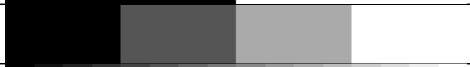 | |
| 4 | 16 | 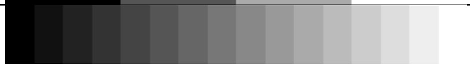 | Grayscale images |
| 8 | 256 | 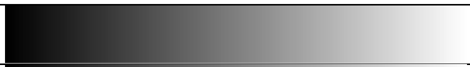 | 1-byte |
| 12 | 4096 | 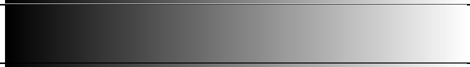 | 2-bytes |
| 16 | 65536 | 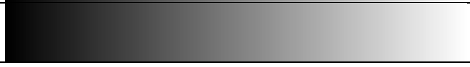 | |

Here is an example how bit-depth will affect the resolution of your images. Original public domain image from: [http://www.public-domain-image.com/computer-arts-public-domain-images-pictures/grayscale-photo.jpg.html](http://www.public-domain-image.com/computer-arts-public-domain-images-pictures/grayscale-photo.jpg.html)

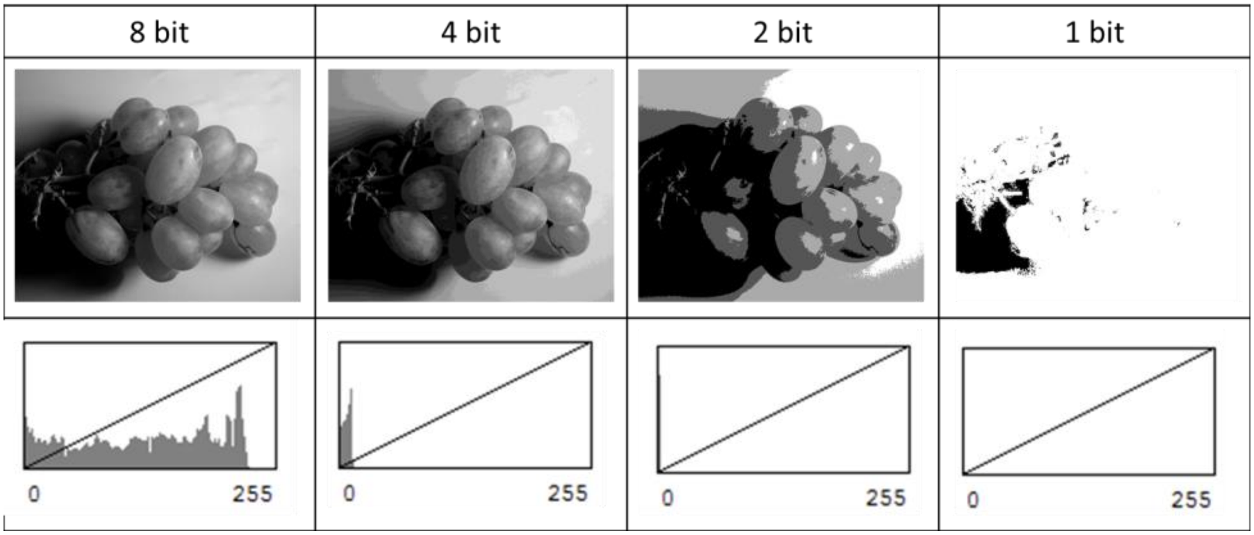


You can see a few differences in the background of the 8 and 4 bit images, but the dynamic range of the information enclosed is a lot smaller for the 2-bit than the 8-bit looking at the analysis of image intensity versus digital numbers. We'll talk about image processing in a later lab, but **remember that to truly understand the resolution of your system, you must look at the intensity values of your pixels! Quantitative imaging is key to getting the most information out of your images!**

It is important to keep this in mind when you are trouble-shooting your optical systems as you may be hitting a resolution limit of your camera/ multiple point detector and not your optics.

**Sources of noise and the signal-to-noise ratio:**
When each pixel of a CCD or CMOS chip reads out signal, noise which is introduced from a variety of sources. Noise is a random background recorded by your CCD.

<u>Read noise</u> comes from the inherent reading out of the CCD and scales with the square root of readout speed (faster cameras have more read out noise).

<u>Dark current</u> is the thermal accumulation of electrons on the chip which also results in noise. Cooling helps reduce dark current so it is negligible in most applications.

<u>Photon Shot Noise</u> is due to the fact that photons are particles collected by the chip and stored in integer numbers. 1 photon doesn't equal exactly 1 count on your image, it depends on the camera gain. Zero photons input into the camera doesn't result in a zero measurement, there is an offset.

Thus, the **Signal-to-Noise Ratio (SNR)** is a good way of comparing the levels of signal to the background noise in terms of power.
Signal = number of photons
Noise = sqrt((read noise)$^2$ + number of photons)
SNR = number of photons/sqrt((read noise)$^2$ + number of photons)

You can see that at low photon numbers, read noise dominates. The threshold for read noise domination is (read noise)$^2$ = number of photons

At high photon numbers, we can neglect read noise:
SNR = number of photons/sqrt((0)$^2$ + number of photons) = number of photons/sqrt(number of photons)
Thus, at high photon numbers, SNR = sqrt(number of photons)

**Some math on image resolution and pixel size (this will be also on your problem set!)**

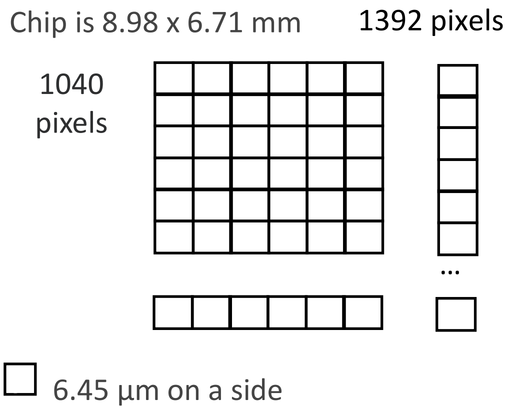


So we have the Sony Interline Chip ICX285 here below, which is 1040 by 1392 pixels, with each pixel a square 6.45 μm on each side. The whole chip measures 8.98 by 6.71 mm. Let's say we project an image magnified by an optical system to 100X onto this chip. What is the resolution of each pixel?

We take the dimensions of each pixel (6.45 μm) and divide by the magnification to yield: 64.5 nm
**So the resolution of each pixel is 65 nm.**

The size of the chip gives us the dimensions of the area of the image we could capture with the whole chip.
1392 pixels * 65 nm/pixel = 90.48 μm
1040 pixels * 65 nm/pixel = 67.6 μm
**The chip can image a field of view of 90.48 μm by 67.6 μm when paired with a 100X magnification system.**

Given a specific feature size (a HeLa cell which is approximately circle ~30 μm in diameter), how many times will it be sampled by our detector + 100X magnification system? (how many pixels will represent the image?)
Area of the cell: pi*r$^2$ = pi*(15 μm)$^2$ = 706.86 μm$^2$

And each pixel has a per-area resolution of 64.5 nm * 64.5 nm = 4160.25 $nm^2$ = 0.00416025 $\mu m^2$
706.86 $\mu m^2$ area of the cell /0.00416025 $\mu m^2$ area imaged by each pixel = **1.7 E5 pixels needed per HeLa cell**
The whole chip is 1392 pixels * 1040 pixels = 1.45 E6 pixels on the whole chip
The HeLa cell image will take up 1.7 E5 pixels / 1.45 E6 pixels on the whole chip = 0.1171
**HeLa cell will occupy 11.7% of our chip, which seems reasonable for comfortable imaging.**

**Digital sampling: what is the optimum number of pixels per image to obtain 'good' resolution?**
Digital sampling is important to deciding on the optimum number of pixels per image to obtain adequate resolution. Resolution is affected by both our optics (which we will discuss in lab 4) and the number of pixels which sample our image. Sampling at a rate which is too low means we will miss information while sampling at a frequency too high will be wasteful and restrict the speed of acquisition. The **Nyquist-Shannon** sampling theorem says that we must have at least 2 pixels per resolvable element. 2.5 – 3 pixels is preferred.

**Light sources:**
The illumination of a sample is important, but so far in lab we have been ignoring this to focus on image formation. We will eventually shape the illumination pathway, but for now let's make a list of things which are important to control for good imaging of our sample:

The intensity (brightness) of the illumination source, its spectrum (wavelengths emitted, color), location (which part of the sample is illuminated?), uniformity (is illumination even?), and angular distribution (from what angles is the sample illuminated?).

No optical system can increase the brightness of a lamp because the area of the light source and angle at which light is emitted is a fundamental constraint.

Natural sources of light include sunlight and fire (candles), used in early microscopy experiments. The sun provides full spectrum but is difficult to harness and fire's spectrum depends on fuel source. Incandescent lamps change the spectrum from the temperature of the filament (cooler filaments produce red-shifted light). Arc lamps are much more controlled sources for light as their spectra can be tightly controlled from the material in the lamp but also through filters.

We will discuss the influence of location, uniformity, and angular distribution of light in a later lab as we learn how to control the illumination part of the microscope. For now, here's an overview of types of modern light sources for imaging**:**

| **Extended Sources** | | | **Scanning systems** |
|---|---|---|---|
| **Arc Lamps** | **LEDs** | **Plasma** | **Lasers** |
| Mercury (Hg): brighter excitation at specific wavelengths | These are solid state sources. Many wavelengths available in blue and red regions. Very broad emission peaks for green and yellow. Have a long lifetime. Not quite bright enough for most microscopy applications. | An arc lamp without electrodes, uses microwave to create a plasma in a quartz bulb. Broadband visible emission and very long lifetime. | Highly collimated, small light source. Many wavelengths available. |
| Xenon (Xe): more stable and longer lifetime than Xe, flat emission in visible spectra | | | Types include: Gas (mainly argon ion), solid state, and diode. |
| Metal halide: best power for commonly used wavelengths (see below). | | | |

When we ray-trace extended light sources, we can think of them as collections of point sources and focus on tracing just some of the points of the source.

**Collimated light sources**

Light whose rays are parallel spread minimally as the light propagates. A perfectly collimated beam has no divergence. Collimated light is said to be focused at infinity, so as the distance from the point source of light increases, the spherical wavefronts assume profiles closer to plane waves (perfectly collimated).

Laser light is produced by an optical cavity and so it is coherent and collimated. Laser diodes (in your pointer, for example) are less collimated because the cavity is shorter and frequently contain a collimating lens.

**Problem Set Questions:**

1.) I am using the Andor iXon3 888 EMCCD camera where each pixel is 13 μm by 13 μm in size. The chip is 1024 by 1024 pixels. If the magnification of my optical system is 50X, what is the field of view that I can image with the chip?

2.) Why are longer exposure times better for decreasing noise in an image?

3.) You have a photodetector (camera CCD chip) with pixels **each** 10 μm x 10 μm in size. You setup an infinite system to focus a sample onto the camera chip with a magnification of 25X. What is the smallest feature on your sample which you can resolve due to limits of the camera?

4.) If the number of photons (photon flux) from your source is constant and high (can ignore read noise), how long do you need to acquire to double your SNR? Assume one acquisition takes some time t and yields N photons.

5.) What is the dynamic range of a system that has full well capacity of 1.6E4 electrons and a readout noise of 5 electrons?

6.) Suggest types of camera chips (CCD, CMOS) or a PMT photodetector for the following scenarios. Limit your suggestion/explanation of mechanism to 50 words for each scenario:

| **Scenario** | **Suggestion & explanation** |
|---|---|
| To count the number of red blood cells in a sample | |
| Histology slide of human intestine | |
| A developing zebrafish embryo | |
| Determine the amount of fluorescent protein on the surface of a petri dish | |

7.) If a collimated light source is said to be focused at infinity, how can we collimate an LED source?

**Background Material & Problem Set for Optics 3: Critical versus Kohler illumination and conjugate planes**

**We expect you to know the following after completing this reading and problem set:**
-explain the difference between critical and Kohler illumination
-point out the benefits of Kohler illumination
-execute the steps to Kohler illumination on your optical setup and a commercial system
-find conjugate planes in an optical system

**Illumination Path Characteristics**

We have learned the basics behind illumination sources in the last reading and setup up our image collection optics with infinity space and camera last lab. Now it is time to learn about how to modify our illumination path to produce even and controlled illumination of our sample for optimum imaging.

In the last reading we listed characteristics were important in the illumination source to get high contrast images but only discussed the first two: intensity (brightness) and spectrum (wavelengths emitted, color). We will now think about the next few factors: location, uniformity, and angular distribution.

Location (where is the light source and which part of the sample is illuminated?)

From your experience in lab, you have probably seen that the location of the light source determines how brightly it shines on the sample. If the light source is far, the light the sample gets is quite dim and this is problematic for transparent or small samples.

But putting a light source right AT the sample is near impossible (plus it would fry your sample due to the heat created by the light source!). What we can do is project an image of the light source onto the sample using a **collector lens.**

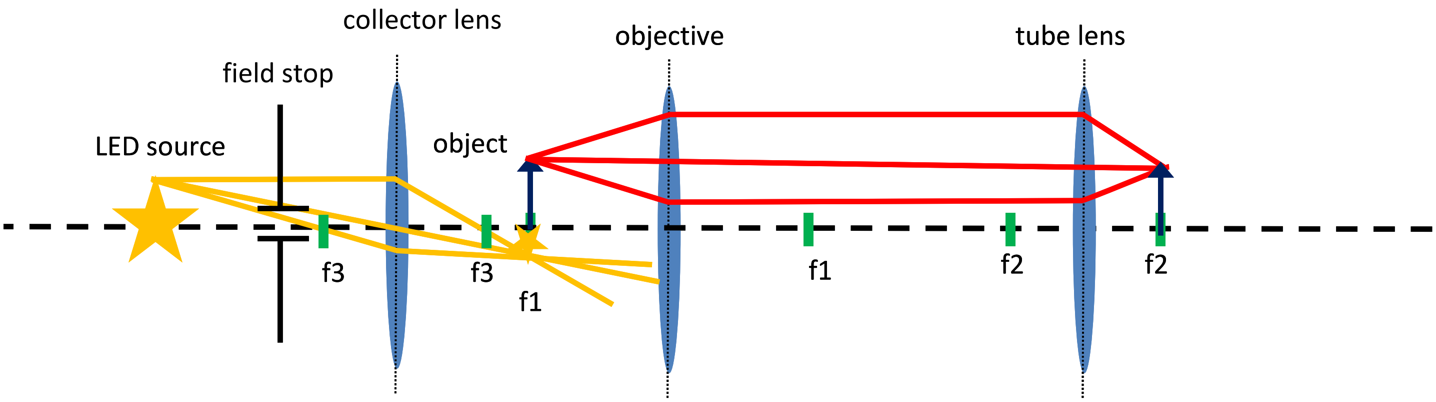

Of course, the rays traced here in yellow extend throughout whole system. I just wanted to leave the ray tracing up to you in the problem set (see problem 1 at end of the reading).

This type of illumination is called **critical illumination** because the object/sample gets superimposed with a real image of the light source. The light source thus is also present on the camera/detector! Our image will thus have a bright light source in focus on it in addition to the sample.

Whenever two planes are imaged onto each other like the sample and light source in the example above, we call this a **conjugate**. **Conjugate planes** are very important in microscopy. You are already familiar with one set of conjugate planes from earlier: the sample plane and imaging screen/camera. When you look into a microscope with your eye, your retina and the sample plane become conjugate planes.

While we are here, let's introduce some more terminology around the collector. If you put an iris behind the light source, it is called a **field stop**. The field stop and light source must be as close to each other as possible because the field stop needs to make the lamp filament appear smaller and sit in the conjugate plane with the sample and camera in this critical illumination setup. The field stop limits the visible area of the lamp because usually your lamp is larger than the sample you're examining. There is a limit on how small you can make the filament. It's sometimes easier to block some light using the field stop instead of using a light source with smaller filament.

<u>Uniformity (is illumination even?)</u>

We already discussed how the huge disadvantage of critical illumination is that now the sample and lamp are in the same conjugate plane. Another disadvantage is that any nonuniformities in the light source are now also apparent in the image since the light source is directly imaged on the detector.

But what can we do to make the light source more uniform? Maybe in lab you noticed that when you put the light source further away, your illumination intensity decreases but the uniformity increases. Moving the LED further and further away from the sample produces more parallel light rays and thus more uniform imaging. **What if the LED was infinitely far from the sample? How can we put the LED at infinity using lenses?** This should give you a hint of what is coming next.

If we place the LED a focal length away from a lens, we can make it look infinitely far away on the other side and present that to our sample (see figure below).

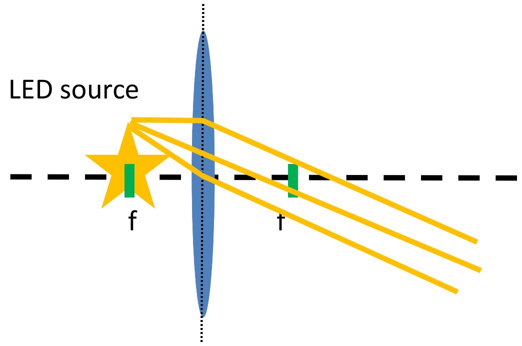


So to evenly illuminate our sample, we can de-focus our light source and collimate it by projecting it to infinity (if we place the light source or its conjugate plane at the focal length of a lens). We can then propagate the light source through our system to ensure that there are no conjugate planes shared with the lamp filament. We will do this fully when discussing Kohler illumination later.

*****Terminology note:** In background reading for lab 1, we introduced the back focal plane (BFP) of a lens, which is located one focal length away from the lens farthest from the lamp. Keep in mind that there is also a front focal plane (FFP) which is one focal length away from the lens closest to the lamp. *******

Angular distribution (from what angles is the sample illuminated?)

The angle of light which is coming from our illumination source is important as it can change which features of the sample are illuminated. You may not see ridges of a sample if you shine light on them "head on" but shining light from the side will help you gain contrast on the features.

Remember that a change in the angle of the light source will result in a displacement on the other side of a lens, as illustrated below. This goes in both directions and so a displacement in the height of the light source in the infinite projection will change the angle of the light at the focal point on the other side of the objective. Thus, when we use apertures in the infinity space to restrict the diameter of the light source, we actually reduce the angle of light Θ at the focal point of the objective.

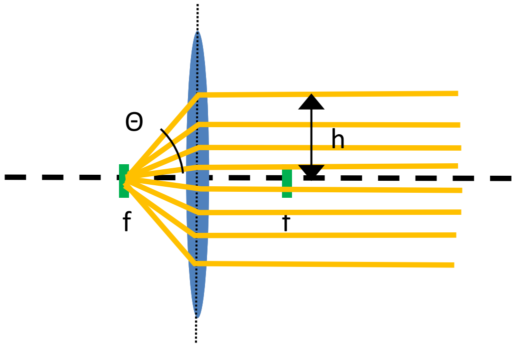


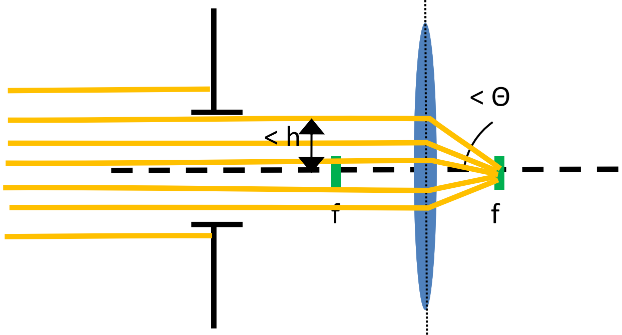

The lens used to control the angular distribution of illumination at the sample is called the **condenser lens**. The **aperture stop** in the front focal plane of the condenser determines the angle or spread of light rays hitting the sample, thus affecting contrast and spatial resolution possible with an optical system.

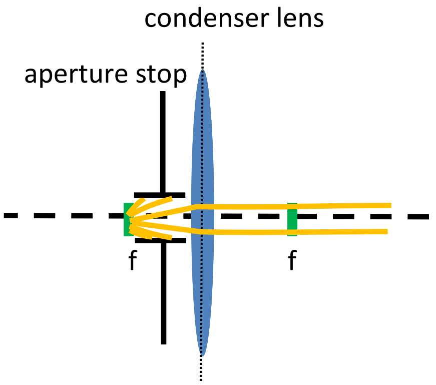


So from this discussion, we want the following in our optical setup:
-defocus the light source when illuminating our sample to take advantage of its intensity without using critical illumination
-use a field stop to control intensity of light and image this onto the camera
-use a condenser system to adjust the angle of light which reaches our sample

A method of illumination which satisfies these goals was developed by August Koehler (also spelled Köhler or Kohler) in 1893. See the diagrams below for how the elements are arranged. The illumination source and sample light rays are traced using different colors to show propagation through the system.

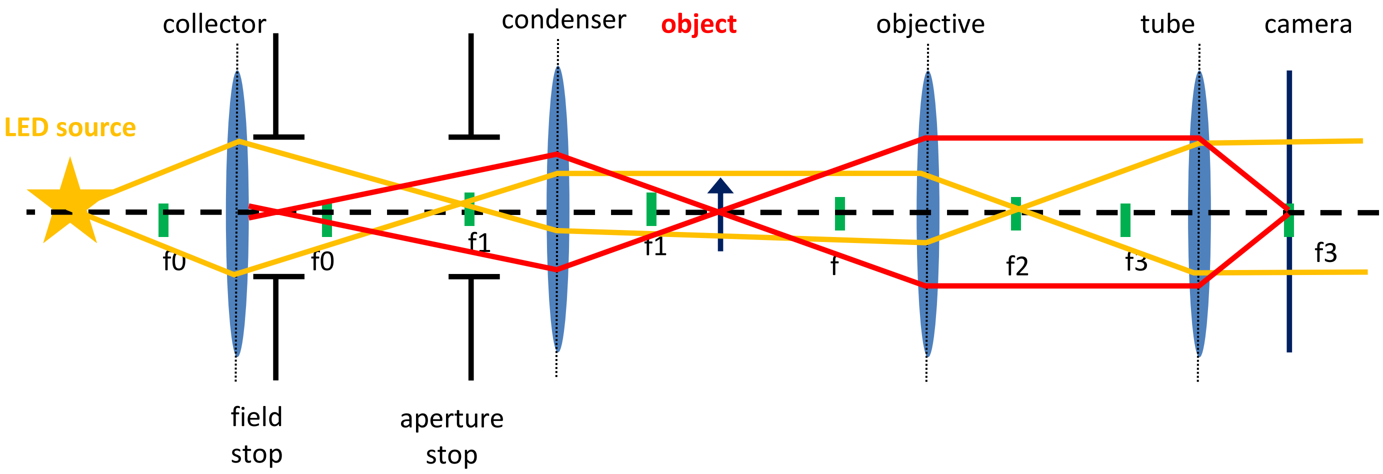


Kohler illumination achieves:
1) bright and even illumination in the sample (and conjugate plane with the detector)
2) positions a conjugate plane with the light source within the microscope so that illumination can be checked and modified without getting imaged on the detector

There is a specific order to which Kohler is set up on a microscope or other optical system containing the components above. Please read through the directions and complete the following tutorial online to become more familiar with the steps: http://www.microscopyu.com/tutorials/java/kohler/

1) Put an illumination source on the edge of your rail and a sample in the middle. Turn on the illumination source.
2) Make sure your tube lens focuses a camera to infinity. Add the tube lens and camera on the opposite edge of your rail. Place the objective between the object and tube lens. Add the collector and condenser lenses as well as the field and aperture stops.
3) Adjust the height of all the optical elements to get the lenses at the same height as the tube lens + camera. It is most difficult to adjust the position of the light source, approximate for now.
4) Make sure all aperture stops are fully open. Makes sure the field stop is as close as possible to the back of the collector.
5) Place your tube lens + camera behind the objective. Focus your objective and sample to get a crisp image on the camera.
6) Put the condenser about a focal length away from the sample.
7) Moving the collector, focus the illumination source at the aperture stop. It is easier to focus the source if it has a particular feature, such as a lamp filament. You can also draw something on the surface, such as a dot/splotch/happy face.
8) Close down the field stop (carefully, do not force it or go too fast as the diaphragm of the iris will break!!!).
9) Moving the condenser, bring the field stop into focus at the sample (and camera, a conjugate plane). You should be able to see the edges of the iris clearly.
10) Make sure the illumination source is still in focus at the aperture stop. You may need to adjust the collector again.
11) Open the field stop enough to illuminate the sample without extra light. Do this by looking at your camera and open the field stop enough such that the edges of the field stop are barely visible on your imaging plane. Close down the aperture stop to optimize the contrast. The aperture stop usually has to be adjusted such that it allows transmission through ~70% of the back focal plane of the objective.

Setting up Kohler is iterative and takes some time to get used to. Once you have the terminology and the steps down, it will take just a few minutes to bring a microscope into Kohler illumination. Make sure you do this every time you approach a light microscope. Commercial microscopes will have ways of adjusting the field and aperture stop as well as the position of the condenser to bring the microscope into Kohler illumination. Commercial microscopes also sometimes have a Bertrand lens to look at the back focal plane of the objective to ensure that you see your light source in focus there. You can also see the back focal plane of the objective by taking out the eyepiece.

Work through the following website to learn how to bring a commercial microscope to Kohler illumination: http://zeiss-campus.magnet.fsu.edu/articles/basics/kohler.html

**Problem Set Questions:**

1.) Please complete the ray trace of the light source throughout the whole optical setup:

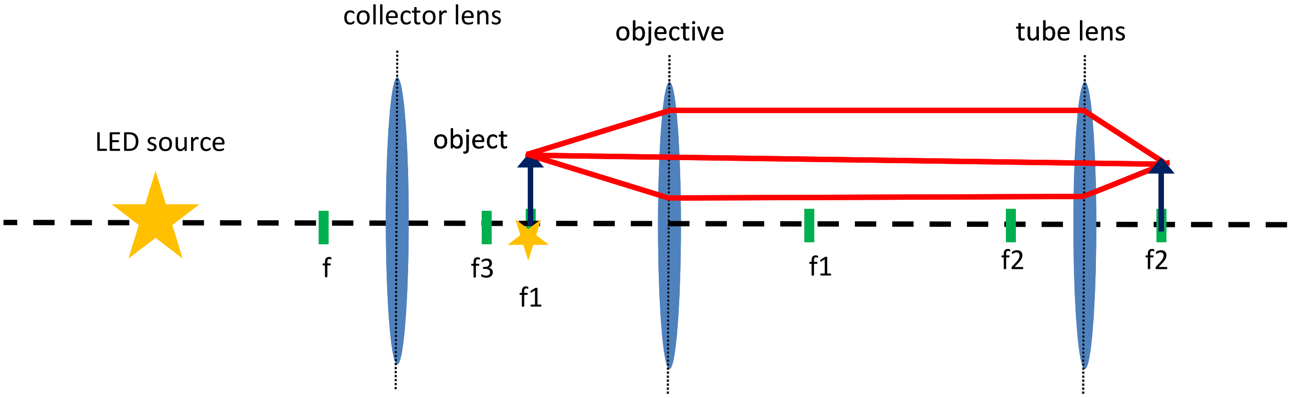


2.) In critical illumination, which planes are conjugate to each other?

3.) Fill out the following table to indicate conjugate planes in a Kohler setup: (hint: use the Kohler diagram to help you identify the conjugate planes via ray tracing)

| Light source | Sample |
|---|---|
| | |
| | |

4.) Give an example of one good and one poor location for a filter to be placed in a Kohler illumination infinity microscope system. Explain your reasoning. Limit your answer to 50 words.

5.) If you change the objective from 10X to 50X on your commercial microscope, will you need to go through the Kohler process again?

6.) What is the main purpose of the field stop and aperture stop? Why should you not use these apertures but rather voltage on the light source to control illumination intensity of your image?

**Background Material & Problem Set for Optics 4: Quantitative microscopy**

**We expect you to know the following after completing this reading and problem set:**

-explain the theory of resolution from perspective of Point Spread Function (PSF) & Airy disks
-link resolution and Numerical Aperture (NA)
-discuss the tradeoffs between resolution & contrast
-discuss the relationship between NA, resolution, & wavelength of light used for imaging
-explain the distribution of high & low resolution light in the back focal plane of the objective (in Kohler)
-explain the basic principles of dark field microscopy
-identify which types of samples dark field would be useful for

## Spatial resolution

We've talked about resolution briefly as it is limited by both the optical components and the imaging sensor (see background reading for lab 2). When we record the last "resolvable" element on our AF 1951 target, we identify the resolution of our whole system. But what is the ultimate limit to resolution optically? How do we determine spatial 2D and 3D resolution?

Spatial resolution is ultimately limited by diffraction. Diffraction from a small, circular aperture results in a point spread function, see figure at right. Thus, the **point spread function (PSF)** is a response of an imaging system to a point source of light (see PSF snapshots in the upper portion of the image below). An Airy disk is the first dark ring of the PSF and the best focused point of light that a lens can make.

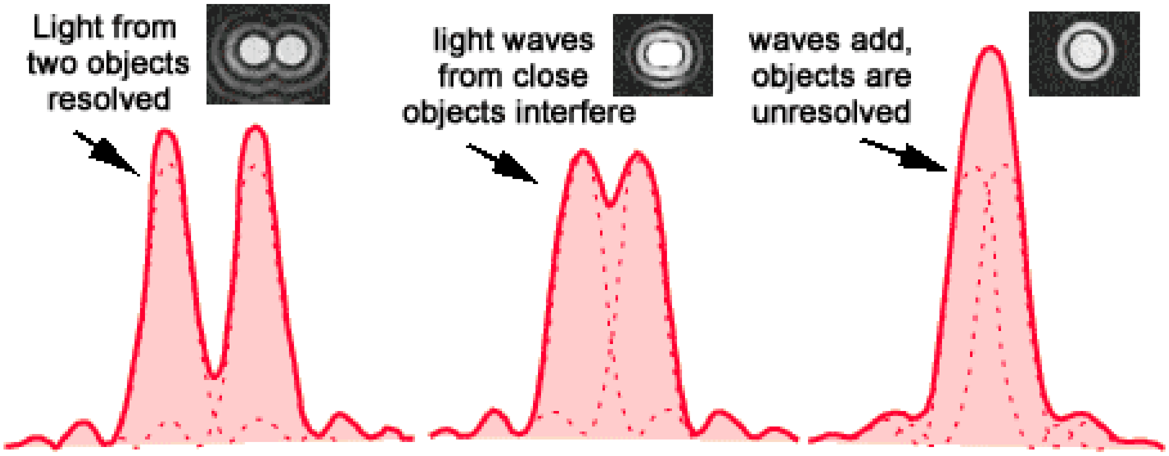


An arbitrary definition of when two features can be resolved is called the **Rayleigh criterion**. The Rayleigh criterion says that two separate point sources can be resolved when the center of the Airy disk from one source overlaps with the first dark ring of the

second (see figure above, available for reuse from http://www.xenophilia.com/zb/zb0012/RayleighCriterion.gif

Another definition for the Rayleigh criterion is the diameter of the Airy disk. The **Full Width at Half Maximum (FWHM)** is a straightforward measurment to make when analyzing the PSF to derive the Numerical Aperture (NA) of your system. Simply find the part of the PSF where you have 50% of the maximum intensity and take the width across, then use the formula FWHM = 0.353 * λ/NA. The formula is based on the typical area under the curve in Airy disk functions. We will cover what NA means in a second.

Here is an example of point spread functions and the Rayleigh criterion for spatial resolution. The top panel contains two unresolved points and the last panel contains two fully resolved points as per the Rayleigh Criterion. Figure is CC from: http://upload.wikimedia.org/wikipedia/commons/a/ae/Airy_disk_spacing_near_Rayleigh_criterion.png

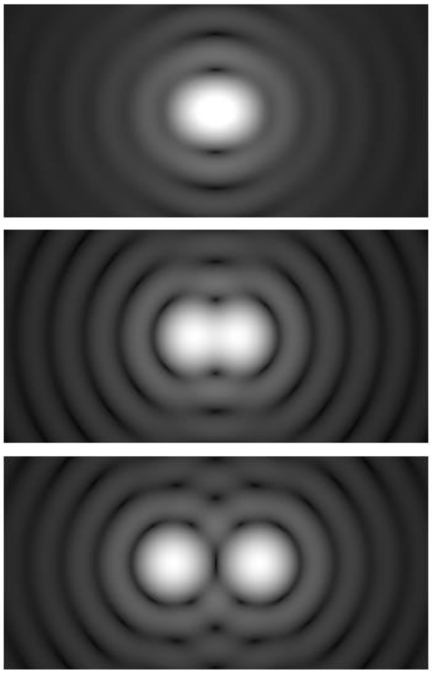

The smallest resolvable distance according to Rayleigh is:

**d = 0.61*λ/NA**

where λ is the wavelength of light used
NA is the Numerical Aperture (see below for discussion)
This 'smallest resolvable distance' d is also sometimes called the "resolving power of the microscope"
The **Abbe limit** is a specification for the resolution of a diffraction-limited microscope is close to the formula above: d = λ/2*n*sin(Θ) = 1/(2*NA)

We will primarily use the Rayleigh criterion to find the resolving power or resolution of our optical setups.

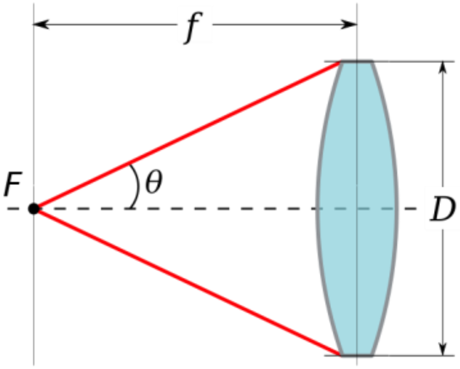


__Numerical Aperture__ is a way to define the cone angle of light which passes through optical system.

**NA = n*sin(Θ)**

where n is the refractive index of the medium between the lens and sample (for air n = 1, oil immersion objectives n = 1.515). Θ is the half angle of the cone of

specimen light accepted by the objective lens – coming from point F in the diagram at the right (CC image from: http://en.wikipedia.org/wiki/Numerical_aperture#mediaviewer/File:Numerical_aperture_for_a_lens.svg).

For brightfield microscopes (the kind we've been using where the condenser NA is less than our objective NA), the resolution of the system is calculated as:
d = 1.22*λ/(condenser NA + objective NA)

NA is usually given for a microscope objective or condenser by the manufacturer and you can use this value for your calculations. But you can also calculate the f-number (denoted as N) using the focal length and the diameter of your lens to find the NA.
N = f/D where f is the focal length and D is the diameter of the lens (keep in mind that D is the entrance pupil diameter of the lens, so if you use a diaphragm to restrict light into your lens, this becomes the effective D).

Then we can follow this derivation to find the NA: NA = n*sin(Θ) = n*sin(arctan (D/2f) ≈ n*(D/2f)
*The trigonometry here looks sketchy, but we can assume that because the angle Θ is small, that the hypotenuse and side of the triangle above is actually equal (both f)*
If we assume we are working in air, n = 1, then NA = D/2f

**Aperture & Resolution:** We have just discussed how numerical aperture plays into lateral resolution through the PSF. Increasing the numerical aperture of the objective makes the resolution of the system higher (meaning that smaller elements can be resolved). You will see this first-hand in the lab as you change the diameter of the aperture at the objective back focal plane.

The Numerical Aperture cannot exceed the lowest n between the sample and the objective lens (because the formula for NA is n*sin(Θ) and thus no matter the Θ, the sin(Θ) will never exceed 1). NA >1 requires fluid immersion (since air has a refractive index of 1).

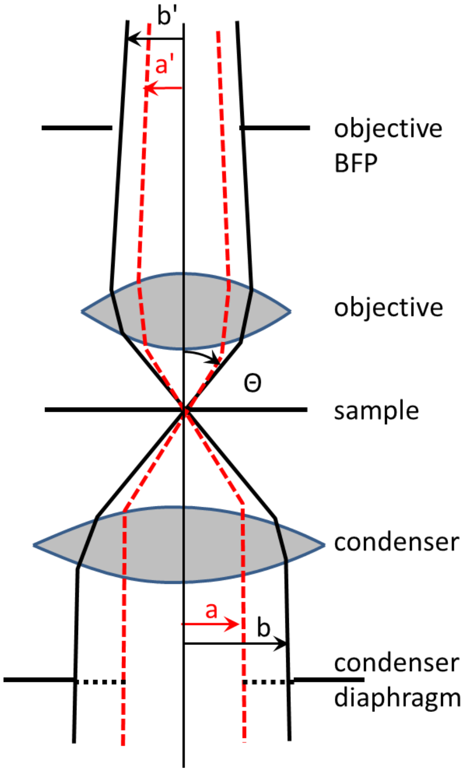


This diagram is my sketch, please find original version with more detail in Murphy's Fundamentals of Light Microscopy and Electronic Imaging.

The condenser aperture directly affects spatial resolution in the microscope. A large aperture angle yields maximum resolution, so the front aperture of the condenser should be fully illuminated for optimum spatial resolution. From the sketched diagram above, closing the aperture stop of the condenser to position a' from position b' limits the angle of illumination, thus reducing the effective numerical aperture. The back focal pain of the objective is no longer filled when the aperture stop is in position a'.

The aperture stop (or condenser diaphragm) limits the number of higher order diffracted rays included in the objective and thus reduces resolution as it is these high order rays that make the point spread function narrow. We can see that if we want to reduce light intensity without affecting our spatial resolution, we should change the voltage supply of the lamp or use neutral density filters rather than using the aperture stop.

**Contrast:**

From the previous paragraph, you might be wondering why we would ever close the aperture stop if it would negatively affect our resolution. But remember in lab 3, we closed the aperture stop to increase the contrast in our images when we put the illumination path into Kohler. It turns out that the high order rays coming into the back focal plane of the objective that pass through the sample provide the most resolution but also "wash out" the image and decrease contrast. Thus, in bright field microscopy it is important to optimize how much the aperture stop is open to ensure that rays of the highest order are blocked to get good contrast yet not block too many so that NA (and thus resolution) of the system is adequate. We will return to thinking about high and low order rays in the back focal plane of the objective when we discuss darkfield illumination.

Contrast is formally defined for two objects of equal intensity as the difference between the maximum and minimum intensity occurring in the space between them. The highest achievable contrast is 1 because the maximum of the Airy disk is normalized to 1.

**Resolution in the Z direction: depth of field & depth of focus**

Diffraction and the wave nature of light act not only laterally, but also along the optical axis. If we are looking at an image and designate points on it as on the XY plane, the Z plane would be perpendicular and lie parallel to the optical axis. The depth of field Z in the sample/object plane refers to the thickness of the optical section along the z-axis within which objects are in focus. The depth of focus refers to the thickness of the image plane itself.

Depth of field $Z = n*\lambda/NA^2$

Thus, larger aperture angles (higher NA) will result in shallower depth of field

Now that we are more familiar with resolution and how it is determined for an optical system, we should discuss more about the roots of these resolution and contrast limits. We will discuss the theory of image formation to understand the distribution of light rays from a sample in the back focal plane of an objective.

Here are the specifications of some common objectives:

| **Magnification** | **NA** | **Resolution (nm)** | **Depth of Field (nm)** | **Light gathering (arb. units)** | **Working distance (mm)** |
|---|---|---|---|---|---|
| 10 | 0.3 | 1017 | 16830 | 0.09 | 15.2 |
| 20 | 0.75 | 407 | 2690 | 0.56 | 1.0 |
| 40 | 0.95 | 321 | 1680 | 0.90 | |
| 40 | 1.3 | 235 | 896 | 1.69 | 0.20 |
| 60 | 1.2 | 254 | 926 | 1.44 | |
| 60 | 1.4 | 218 | 773 | 1.96 | 0.21 |
| 100 | 1.4 | 218 | 773 | 1.96 | 0.13 |

Objectives also have a specification called "working distance" which is the distance from the front lens element of the objective to the closest surface of the coverslip when the specimen is in sharp focus (see diagram at right). Long working distance allows greater access to samples which are thick in the z dimension.

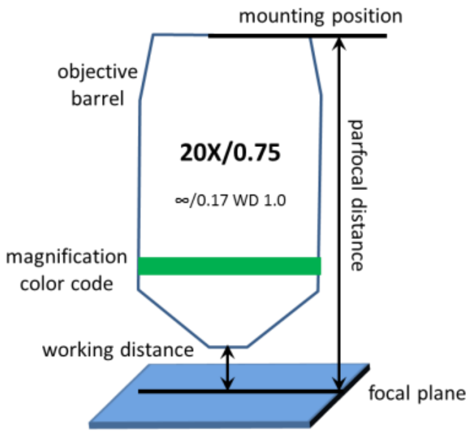


In general, high NA lenses have short working distances. However, extra-long working distance objectives do exist (but are very expensive).

**Aperture, resolution, & contrast**

In an earlier lab, we said that the aperture stop can be closed to increase contrast but this decreases resolution. We can now see that if we fully open the aperture stop, we will increase the NA of the condenser (which is good for improving our resolution) but this is at the expense of having good contrast in our system since then the illumination may overexpose our sample. This tradeoff is important to keep in mind.

**Brief review of image formation:**
Remember that when collimated light passes through an aperture, it deviates and diffracts. Some of the light will not diffract and pass un-deviated through the aperture (see diagrams below, image on right CC image below from http://upload.wikimedia.org/wikipedia/commons/b/bb/Water_ripples_Diffraction.png).

These will be called the $0^{th}$ order light rays. The $0^{th}$ order rays will be out of phase with the diffracted light to varying degrees. When the light diffracted by the specimen is brought into focus on the same image plane, destructive interference occurs and thus the intensity of light in those areas decreases. These areas appear darker and it's that darkness compared to bright areas that we perceive as contrast which leads to us recognizing an image of the sample.

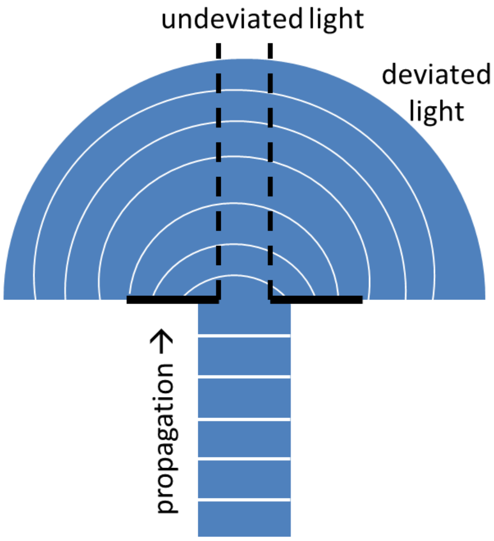


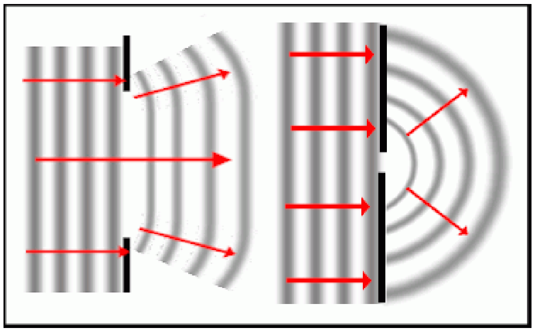

**Ernst Abbe** demonstrated using diffraction gratings that interference between the $0^{th}$ and higher order diffracted rays in the image plane generates image contrast and determines the limit of spatial resolution of the objective.

Collimated beams (planar wavefronts) illuminating periodic rulings show light diffracted into specific patterns in the back focal plane of the objective. Higher order light rays were towards the edges of the back focal plane while un-deviated light rays appeared in the middle ($0^{th}$ order). The diagram below illustrates Abbe theory of image formation where angles Φ between light scattered by the sample correspond to distances S between where light of different orders focuses.

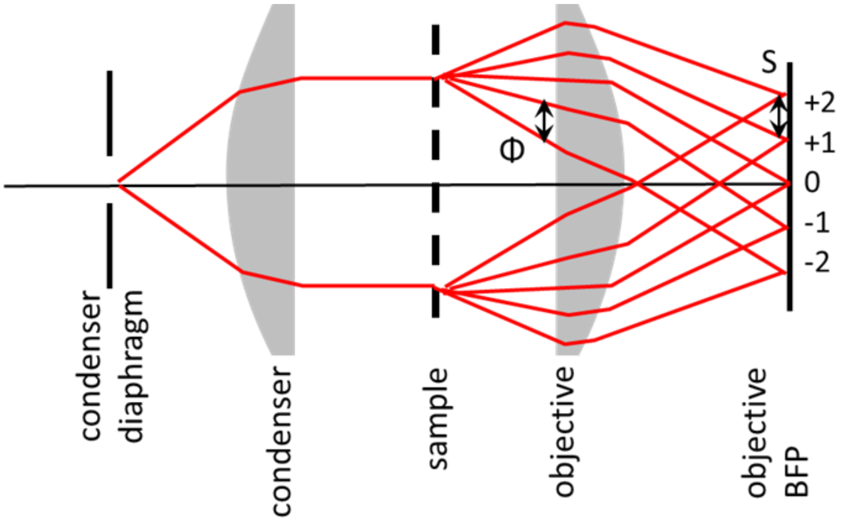


Abbe showed that when diffraction gratings (samples containing spaced lines) are placed in the specimen slot, several aperture stop (condenser iris) images form in the back focal plane (BFP) of the objective (see below). **These iris images correspond to $1^{st}$, $2^{nd}$, and higher order diffracted rays on both sides of the un-deviated $0^{th}$ order beam.** For more detail, see Murphy's Fundamentals of Light Microscopy and Electronic Imaging.

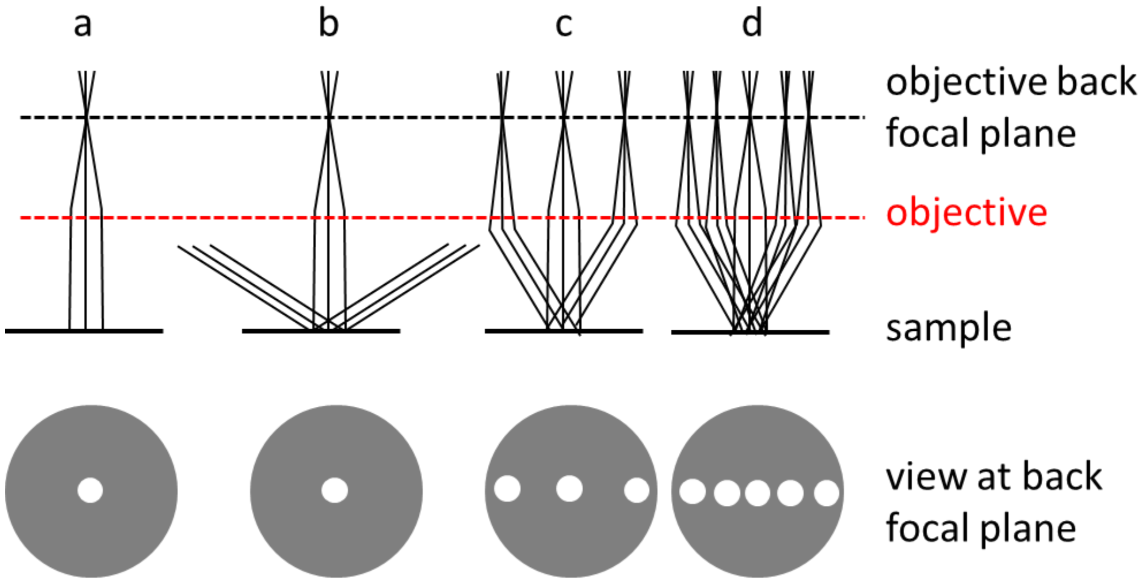


| A | B | C | D |
|---|---|---|---|
| No image formed because diffraction of the sample doesn't occur | No image formed because diffracted rays are not collected | Minimum of 2 adjacent diffraction orders ($0^{th}$ and $1^{st}$) form an image | Multiple diffracted orders are collected, high degree of definition in the image |

The larger the spacings between the lines in the diffraction grating, the smaller the degree of separation between the iris images in the BFP. **Blocking out specific order spots in the BFP results in global changes to the image across the whole field of view.** Blocking high order light rays in the BFP decreases the resolution features of the image. This is why adjusting the aperture stop such that the BFP is 70% open in Kohler illumination results in a decrease in resolution of the system yet optimizes contrast.

Different gratings produce different patterns in the objective BFP and the processing of where the high frequency (and high order) light rays reside in these images has to do with Fourier transformations. We won't go into it in detail here, but see Davidson and Abramowitz's review "Optical Microscopy" for a primer.

Read more about Abbe and image formation here:
http://micro.magnet.fsu.edu/optics/timeline/people/abbe.html.

**So far: transmitted light microscopy**
We have so far exclusively used transmitted light microscopy where objects are illuminated by a uniform light source and both diffracted light rays (which interacted with the specimen) and nondiffracted rays (ones which pass un-deviated through the sample) are collected by the objective to form the image. For unstained, transparent samples, the amount of nondiffracted light is large which means that images re bright and low in contrast.

So how can we increase the contrast for samples which are transparent and have fine features? This is where modifying the back focal plane of the objective comes in handy to ONLY collect light diffracted by the sample.

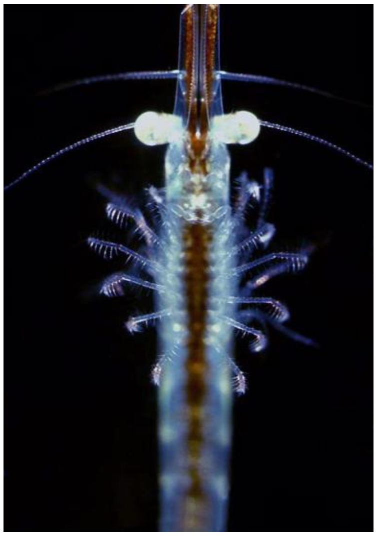

**Dark field microscopy** is useful for viewing objects which are transparent by only capturing the diffracted light rays. Nondiffracted light rays are removed altogether by using a mask in the back focal plane of the objective. Small amounts of diffracted light are clearly seen against a black/dark background. Dark field microscopy is used to inspect isolated cells, organelles, and polymers. On the right is a dark field image of a mysis zooplankton (CC image from http://en.wikipedia.org/wiki/Dark_field_microscopy#mediaviewer/File:Mysis2kils.jpg). Dark field images of diatoms, a common phytoplankton, are especially impressive and we encourage you to search for these examples online.

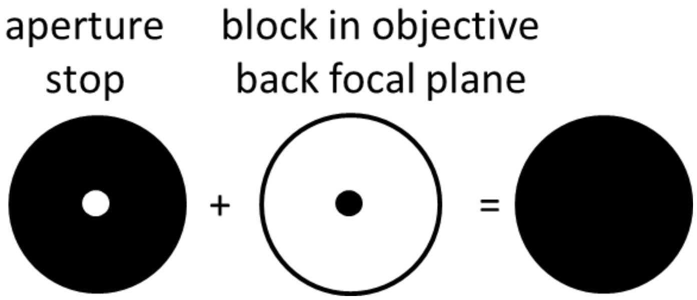


Dark field imaging can be achieved by stopping down the condenser and then blocking a matching area of the condenser in the back focal plane of the objective using a mask (see diagram below). The result is that light of a low numerical aperture shines on our sample and the nondiffracted light is completely blocked from our detector. Only light directly diffracted by the sample gets collected.

Dark field microscopy is ideal for small particles, unstained microorganisms, etc. Dark field is difficult to execute for large objects due to limited illumination area (since the condenser has to be stopped down) and because signal is low since only light directly diffracting from the sample is detected.

**Problem Set Questions:**

1.) How can you decrease spherical aberrations in your system? What is the drawback of this approach?

2) Calculate the missing element of the following objectives:

| **Magnification** | **NA** | **Θ** |
|---|---|---|
| 100X | | 71.8° |
| 4X | 0.20 | |
| 20X | 1 | |
| 10X oil immersion | | 60° |

3.) What sets the smallest details that you can resolve in an image? Magnification or numerical aperture?

4.) Resolution from the optics and imaging perspectives:
a.] What is the resolution of an objective with NA = 1.4 imaging samples using 500 nm laser light?
b.] According to the minimum requirements of the Nyquist-Shannon sampling theorem, _______ nm of your image must cover_______ pixels
Hint: think back to lab 2 background reading
c.] Your camera chip's pixels are squares which measure 6.45 μm on each side. What is the proper magnification needed to achieve the Nyquist-Shannon sampling theorem criteria?

5.) Why would a 20X lens undersample your image if you are using a 1.6 NA objective with 500 nm laser light? Would we be limited by our optics or camera resolution?

**Background Material & Problem Set for Optics 5:** Diffusion & Brownian motion

**We expect you to know the following after completing this reading and problem set:**
-follow derivations of the Boltzmann distribution (from random walk)
-understand and be able to use the basic concepts of Fick's laws of diffusion
-describe how the Einstein relation incorporates the diffusion constant and drag coefficient
-describe how to measure the diffusivity of a particle in a solution from its coordinates of displacement vs time
-use the Einstein relation to derive the size of a particle from diffusivity (and vice versa)

**Big Picture:**
In this background, you will notice that we will go through a lot of derivations and I want to give you the point early on of why we are doing this. In Optics Lab 5, you will use your optical system to track the movement of beads due to Brownian motion (thermally-induced random movement). From the displacement of the beads over time, you will be able to calculate the diffusion coefficient of your beads in solution using the Stokes-Einstein relation. We want you to understand the origin of the equations we use, so we will start with explaining random walks, how they relate to diffusion, and finish with a derivation of the diffusion coefficient of a solution based on a particle size and contribution of fluid to viscous drag on the particle.

**Transport Process** [adapted from Truskey's Transport Phenomena in Biological Systems]:
For the cells, tissues, and organs within your body to function properly, a constant supply-exchange of nutrients and wastes must be moving rapidly to/from them. Lung, liver, and kidney tissue is specially organized (cells with large surface area, microvilli) to enable rapid exchange of molecules between the blood and tissues. The density of capillaries in many tissues and the size/structure of cells can be explained by the rates of transport of molecules to these sites. There is a whole field dedicated to studying transport phenomena in tissues and involves integrating momentum, mass, energy balances, kinetics of reactions, and thermodynamics.

Two physical phenomena are involved in transport processes: diffusion and convection. Diffusion involves random motion of molecules due to thermal energy transferred by molecular collisions. Convective transport results from bulk motion of fluids. For this class, we will focus on diffusion and how to measure it using the optical systems you have built in lab. For the discussion below, solute refers to a substance which is dissolved by a solvent. For example, table sugar is a solute dissolved in a water solvent.

**Random Walk:**
Molecules in a solution are in constant motion, even if the fluid is not flowing. These molecules collide trillions of times per second and each collision results in random motion of the molecules, giving rise to diffusion. Solutes in a fluid are constantly being pushed around by molecules of the solvent. Even though the molecular collisions are random, the result is net movement of the molecules. We call the net molecular motion arising from molecular collisions a 'random walk' (see diagram at right from Truskey). The net movement of a molecule between two points after some number of steps is characterized by root-mean-squared (RMS) displacement ($\sqrt{< x^2 >}$) where *x* is the total average distance traveled by the molecule. But since half of the motions were in the positive or negative directions, we consider the displacement to be the RMS of the total distance traveled. Here's more on random walks (and also in 6.5 of Truskey): http://www.mit.edu/~kardar/teaching/projects/chemotaxis%28AndreaSchmidt%29/random.htm

**Brownian Motion:**
Random motion of particles suspended in a fluid (liquid or gas) due to their collisions is called Brownian motion, named after botanist Robert Brown who first observed the effect in 1828 while looking at pollen grains in water. Large groups of particles, each one moving randomly, show the Brownian motion behavior due to statistics (Brownian motion is a simple stochastic process). CC image at right from: http://en.wikipedia.org/wiki/Molecular_diffusion#mediaviewer/File:DiffusionMicroMacro.gif

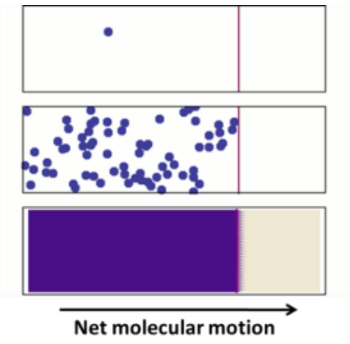


Yet the random movements of the molecules on the microscale lead to macroscopic observations. What happens if you put one drop of food coloring in a glass of water? We know that after a while, the drop will diffuse in to the glass and the whole volume will be evenly colored. Large groups of diffusing molecules tend to move from a region of high concentration to a region of low concentration. But why is this the case? The answer lies in Fick's laws of diffusion.

**Diffusion:**
We will describe this process using two approaches**. First by random walk**: A molecule travels distance δ over a time interval τ (n number of steps) before encountering another collision. Since the angle of collision is random, so will be the direction of motion. Energy transferred will follow the probability distribution above and so the time and distance traveled between collisions will be random. We can follow a random walk

argument (which I will not detail here) and arrive at the following relationship (assume 1D motion):

$$\sqrt{<x^2>} = \sqrt{n\delta^2} = \sqrt{n}\delta$$

After $n$ steps, the elapsed time $t = n\delta.$ And so $n = t/\tau$, thus:

$$\sqrt{<x^2>} = \delta\sqrt{\frac{t}{\tau}}$$

$$<x^2> = \delta^2\frac{t}{\tau}$$

Now we define a diffusion coefficient for 1D random walk as

$$D_{ij} = \frac{\delta^2}{2\tau}$$

And so we have:

$$<x^2> = 2D_{ij}t$$

This equation indicates the mean-squared distance sampled by the molecule increases linearly with the square root of time. The factor of 2 accounts for negative and positive directions (in +x or -x). For 3D diffusion, you can use the following relationship:

$$<r^2> = <x^2> + <y^2> + <z^2>$$

And if you follow through the same formulas above, you will derive mean square displacement in 3D:

$$<r^2> = 6D_{ij}t$$

Diffusivity ($D_{ij}$) is a measure used to characterize the ease with which a solute diffuses through a solvent or membrane. The units of diffusivity are: $m^2/s$

**Now we look at diffusion again using Fick's 1st law (1 dimensional in Cartesian coordinates):** Let's define flux, (J) which is the number of particles (n) crossing perpendicular per unit area (A = $m^2$) per unit time.

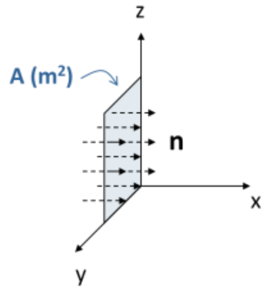


Let's look at the system in 1D. Let N(x) equal the number of particles at location x. We also have some step size δ away from x where the concentration of particles is N(x+ δ)

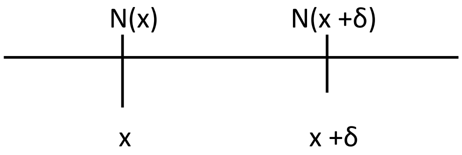

δ is the stepping size and τ is the stepping time. The net flux in the x direction is given by:

$$J_{x\ direction} = \left\{\frac{1}{2}N(x) - \frac{1}{2}N(x+\delta)\right\}\frac{1}{A\tau}$$

We have the ½ factors because we expect half of the particles to cross to the right and half to the left because the movement is completely random. We know that concentration of particles in an area is a function of the volume of the section we are looking at (Aδ) and the number of particles there (N(x)). Let's get $C(x) = \frac{N(x)}{A\delta}$ in the net flux formula:

$$J_x = \frac{-\delta}{2\tau}\left\{\frac{N(x+\delta)}{A\delta} - \frac{N(x)}{A\delta}\right\}$$

$$J_x = \frac{-\delta}{2\tau}\{C(x+\delta) - C(x)\}$$

We also know that $D_{ij} = \frac{\delta^2}{2\tau}$ from our random walk analysis above:

$$J_x = -D_{ij}\left\{\frac{C(x+\delta) - C(x)}{\delta}\right\}$$

We can see that$\left\{\frac{C(x+\delta)-C(x)}{\delta}\right\}$ is the definition of the derivative of C with respect to x such that:

$$J_x = -D_{ij}\left\{\frac{\partial C}{\partial x}\right\}$$

We just derived Fick's 1st law which relates the diffusive flux to the concentration. This law shows us that flux goes from high to low concentration regions (due to the negative sign showing the direction of flux). The flux of molecules results in a concentration gradient ($\frac{\partial C}{\partial x}$).

Now we can also derive Fick's 2nd law. Imagine a membrane which devices two 'baths' of liquids. The total number of particles must be conserved as they travel through the membrane.

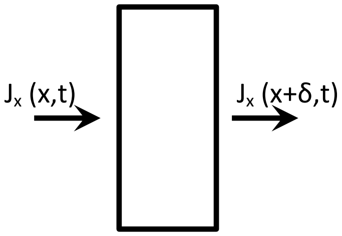


Then we can write the following formula:

$$\{C(t+\tau) - C(t)\}A\delta = J_x(x,t)A\tau - J_x(x+\delta,t)A\tau$$

$$\left\{\frac{C(t+\tau)-C(t)}{\tau}\right\} = \left\{\frac{J_x(x,t)-J_x(x+\delta,t)}{\delta}\right\}$$

$$\left\{\frac{C(t)-C(t+\tau)}{\tau}\right\} = -\left\{\frac{J_x(x,t)-J_x(x+\delta,t)}{\delta}\right\}$$

$$\left\{\frac{\partial C}{\partial t}\right\} = -\left\{\frac{\partial J}{\partial x}\right\}$$

And we know already from Fick's 1st law that $J_x = -D_{ij}\left\{\frac{\partial C}{\partial x}\right\}$
So we substitute to derive Fick's 2nd law:

$$\left\{\frac{\partial C}{\partial x}\right\} = D_{ij}\left\{\frac{\partial^2 C}{\partial x^2}\right\}$$

Fick's 2nd law shows how diffusion causes concentration to change over time.

You might have noticed in your daily life that diffusion happens faster in warm versus cold liquids (think about how long it takes a teabag to 'brew' (evenly disperse) in cold versus hot water). Speed of the diffusion gradient depends on:
-temperature
-size of the solute and solvent (as well as their interactions)
-interactions between solute and solvent
-barriers such as membranes which also provide size and interaction effects

**Boltzmann Distribution:**
As we mentioned above, diffusion on the molecular scale involves molecules undergoing random interactions. Thus, we can use probabilities to describe the average position or state of the molecules. We also mentioned that the diffusion arises from molecular interactions of thermal origin. Thermal energy is transferred to kinetic energy when the molecules collide. We can state that a molecule has energy $E_i$ following the Boltzmann distribution:

$$e^{\frac{-E_i}{kT}}$$

Where k is the Boltzmann's constant 1.38 E-23 J/molecule*K, E is state energy (which depends on the state), and K is temperature in Kelvin. From the formula above, we can see that if the temperature is 0K (absolute zero), we get the minimum energy for the system ($e^0$ = 1). Such a system is impossible to build (you can't escape thermal fluctuations). Current laser cooling techniques can produce temperatures less than a billionth of a kelvin (1E-9K) and the current world record (set in 1999) is 100 picokelvins (1E-10 K).

Following this, the probability that a molecule has energy $E_i$ is $p(E_i)$

$$p(E_i) = \frac{e^{\frac{-E_i}{kT}}}{\sum_{i=1}^{n} e^{\frac{-E_i}{kT}}}$$

Thus, the system receives energy by random collisions between molecules due to thermal contributions. Because the exponent is negative on the energy relation, there is little chance of the system receiving so much energy from molecular collisions such that the energy of the system is greater than kT. The system will most likely receive energy of the order of kT due to thermal contributions.

**Stokes-Einstein relation to get particle diffusivity or size:**

We will now combine the results of our random walk calculations and derivations of Fick's laws with drag on a sphere at low Reynolds number (flow dominated by viscous parameters, no inertial flow) to see how we can estimate the diffusion coefficient of solutes. It turns out that the diffusion coefficient ($D_{ij}$) is not just affected by the random walk, but also differences in the solute size and noncovalent interactions with the solvent (van der Walls forces). The diffusion coefficient is a function of temperature, fluid viscosity, and solute size. To get an idea of how these factors impact the diffusion coefficient, check out the following table, copied from Truskey's Transport Phenomena in Biological Systems

| **Binary diffusion coefficients of molecules in water at infinite dilution at 25°C** | |
|---|---|
| **Solute** | **D (1E-5 $cm^2$/s)** |
| Oxygen | 2.10 |
| Sucrose | 0.52 |
| Urea | 1.38 |
| Glycine | 1.06 |
| Ethanol | 0.840 |
| Albumin | 0.061 |
| Hemoglobin | 0.069 |
| Fibrinogen | 0.020 |

For low Reynolds number flow, drag force ($F_{Drag}$) on a particle is proportional to the particle velocity:

$$F_{Drag} = fv$$

Where $f$ is the friction coefficient (for a uniform istotropic body) and $v$ is the particle velocity. For a sphere of radius R, $f = 6\pi\mu R$ where μ is the viscosity of the fluid.

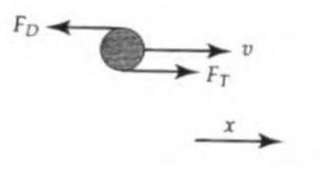


We find that at equilibrium, the concentration profile is uniform unless an external force is applied to the system. When the external force is applied (drag on the particle), a negative flux must balance it out to bring the system to equilibrium. Thus:

$$J_{Drag} + J_{Diffusion} = 0$$

Local force on a particle would be $F_{Drag} = -\frac{\partial E}{\partial x}$ as E is the energy function of the system

$$J_{Drag} = vC(x) = \frac{F_{Drag}}{f}C(x) = -\frac{\partial E}{\partial x}\frac{C(x)}{f}$$

As before by Fick's 1st law:

$$J_{Diffusion} = -D_{ij}\left\{\frac{\partial C}{\partial x}\right\}$$

Now we substitute:

$$J_{Drag} + J_{Diffusion} = 0$$

$$-\frac{\partial E}{\partial x}\frac{C(x)}{f} - D_{ij}\left\{\frac{\partial C}{\partial x}\right\} = 0$$

From the Boltzmann distribution discussion above:

$$C(x) = Ae^{\frac{-E_i}{kT}} \text{ where A is a constant}$$

$$\frac{\partial C}{\partial x} = \frac{-1}{kT}\frac{\partial E}{\partial x}Ae^{\frac{-E_i}{kT}}$$

$$\frac{\partial C}{\partial x} = \frac{-1}{kT}\frac{\partial E}{\partial x}C(x)$$

Now substitute back into the flux balance:

$$-\frac{\partial E}{\partial x}\frac{C(x)}{f} + D_{ij}\left\{\frac{-1}{kT}\frac{\partial E}{\partial x}C(x)\right\} = 0$$

$$-\frac{\partial E}{\partial x}\frac{C(x)}{f} = D_{ij}\left\{\frac{-1}{kT}\frac{\partial E}{\partial x}C(x)\right\}$$

$$\frac{1}{f} = D_{ij}\left\{\frac{1}{kT}\right\}$$

$$\frac{kT}{f} = D_{ij}$$

As before, for a sphere of radius R, $f = 6\pi\mu R$(where μ is the viscosity of the fluid). So in the end we have the Diffusivity in terms of the Stoke's relation:

$$\frac{kT}{6\pi\mu R} = D_{ij}$$

**What do the RMS random walk and Stoke-Einstein relations enable us to do?** We can measure diffusivity (D) experimentally using the RMS relationship ($< x^2 >= 2D_{ij}t$ ) knowing the bead's displacement through time (plugging in x and t into the formula). We can then verify our calculating using this Stokes-Einstein relation $\frac{kT}{6\pi\mu R} = D_{ij}$ to get the theoretical D we would expect based on the particle size (which we know).

**Problem Set Questions:**

**1.)** How long would it take for (deoxy)hemoglobin (64.7 kDa) to diffuse across an erythrocyte (red blood cell, 8 µm) in the body (37°C)? Assume the fluid inside of the RBC is close to water with viscosity 1 mPa*s. Use diffusion in 1 dimension (across the red blood cell). Estimate the diffusion coefficient of hemoglobin by calculating the volume occupied by a protein of mass M (in Daltons) as follows (formula origin here). The $0.73\frac{cm^3}{g}$ comes from partial specific volume occupied by a protein (be careful to plug in Daltons [Da] and not kDa):

$$Volume\ [nm^3] = \frac{0.73\frac{cm^3}{g}(1E21\frac{nm^3}{cm^3})}{6.023E23\frac{Da}{g}} = 1.212E-3\frac{nm^3}{Da}) * MW\ [Da]$$

To find the radius, approximate the protein as a sphere.

**2.)** Extension on question 1: how long would it take the hemoglobin to diffuse in 3 dimensions? How does the time compare to 1D diffusion?

**3.)** Explain how random motion of molecules produces a concentration gradient and net movement of solute.

**4.)** You are Robert Brown, observing pollens move about on your microscope in the lab. You construct the following graph. What is the slope of the graph and what does it tell you about diffusivity?

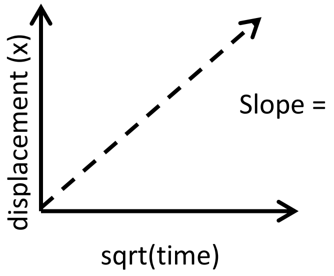


**5.)** If you turn off the microscale propellers on bacteria (flagella, cilia), then how long would the bacteria coast without stopping (in seconds)? Assume the bacteria is a sphere 1 micron in diameter. The bacteria is equal in density to water (1 g/cm$^3$). Assume the viscosity of water is 0.001 Pa*s. Hint: start with Newton's second law and balance force of the bacteria propelling through the fluid and drag on the bacteria from the fluid.

**6.)** Extension of question 5: how far does the bacteria coast before it comes to a complete stop? The bacteria moves at an initial velocity of 30 microns per second
