# Supplementary material for: A Biotic Game Design Project for Integrated Life Science and Engineering Education
Source: PLoS Biol. 2015 Mar 25;13(3):e1002110. doi: 10.1371/journal.pbio.1002110 (PMC4373802; doi:10.1371/journal.pbio.1002110)
Supplement: S19 Text — This document provides all laboratory instructions for the optics section of the course. (DOCX) [file pbio.1002110.s019.docx]

**Optics Labs Sections for BioE 123 Course developed at Stanford University**

Instructors: Ingmar Riedel-Kruse, Gabriel Sanchez, Stephen Quake

Compiled and updated by: Aleksandra K. Denisin

Aleksandra K. Denisin would like to acknowledge Prof. Dan Fletcher and Neil Switz, whose practical lab microscopy class at UC Berkeley provided inspiration for the following optics lab modules.

**Copyright statement:** All images and figures included here have been generated by the BioE 123 teaching team, except where otherwise noted.

**Overview:** Students will learn the basic fundamentals of optics and integrate conceptual understanding with hands-on experience in designing and building optical systems for microscopy. There are 5 optics modules with the following learning objectives:

**1] Optics basics, focal lengths of lenses, finite imaging, exploring the thin lens equation, building an finite optical system**

***Learning Objectives:***

-how lenses shape light, ray tracing, Snell’s law

-finite optical systems & the Thin Lens Equation

-magnification

-origins of spherical aberrations

**2] Setting up a camera focused at infinity, exploring magnification in a simple microscope, identifying infinity spaces and practicing ray tracing**

***Learning Objectives:***

-how cameras record images

-“imaging at infinity” using a tube lens

-properties of lenses, how focal lengths can help magnify or enlarge objects/light sources

-infinite optical systems & why they are useful

**3] Setup Kohler illumination, explore conjugate planes, dark field and oblique illumination**

***Learning Objectives:***

-critical vs. Kohler illumination, steps of how to get a microscope into Kohler

-conjugate planes & image formation

-how to setup darkfield illumination & why it is useful for some specimens

**4 & 5] Quantitative microscopy, imaging the Point Spread Function, exploring resolution versus contrast**

***Learning Objectives:***

-theory of resolution from perspective of Point Spread Function (PSF) & Airy disks

-tradeoffs between resolution & contrast

-how to practically measure the PSF of any optical system (using dispersed beads)

-distribution of high & low resolution light in the back focal plane of the objective (in Kohler)

**Parts/Equipment:** part names/numbers correspond to Thorlabs catalog. The following parts are for 1 teaching setup

1. **Aluminum Breadboard, 12" x 12" x 1/2", 1/4"-20 Taps** (MB12)

4 Tennis ball halves keep the breadboard from scratching table top & help with damping

1. **Light Source & Webcam**

Our students built their own light source from 3 LEDs arranged in a custom laser cut holder. You can also use a commercial light source for bright and consistent signal like the 525 nm Green LED Array Light Source (LIU525A). *Warning:* the LIU525A light source is very powerful and students should be fully instructed on safety before operating this equipment.

We used a Logitech QuickCam Pro webcam from which the CCD chip could be extracted from the housing to attach to the back of a tube with adjustable lens position to focus the camera chip at infinity using the tube lens.

Large V-Clamp with PM4 Clamping Arm, 2.5" Long (VC3)

1. **Rail System**

1 Dovetail optical rail, 24” imperial (RLA2400) & 2 14” imperial rails (RLA1200)

12 Dovetail clamps, 1” (RC1)

1. **Posts & Post Holders**

10 post holders with spring loaded hex-locking thumbscrew, 3” long (PH3)

10 Ø1/2" x 4" Stainless Steel Optical Post, 8-32 Stud, 1/4"-20 Tapped Hole (TR4)

1. **Lenses & Lens Mounts**

3 lens mounts (LMR1)

3 plano-convex lenses, uncoated (LA1986 f = 125 mm, LA1509 f = 100 mm, LA1131 f = 50 mm, LA1951 f = 25.4 mm)

2 achromatic doublet lenses (AC254 -100 - B, AC254 - 60 - B)

5 Mounts for lenses (LMR1)

1. **Stage & Target**

Stage/microscopy slide holder (similar to MAX3SLH)

Positive 1951 USAF Test Target, 3" x 3" (R3L3S1P)

1. **Apertures**

3 Post-Mounted Iris Diaphragm, Ø25.0 mm Max Aperture  (ID25)

1. **Mirrors**

1 mounted mirror, protected silver (PF10-03-P01P)

1 45 degree mount for mirror (H45)

1. **Lens Tube Holders**

Tubes (SM1L30, SM1V10, SM1L10, SM1L40)

1. **Converters**

4 Right-Angle Post Clamp, Fixed 90° Adapter  (RA90)

1. **Lens Mounting Tools**

1” lens mounting tool (SPW602)

1. **Bases**

5 fork bases (BA1S)

1. **Objective lenses**

100x and 4x Nikon objectives

1. **Filters**

Ø25 mm BG18 Colored Glass Bandpass Filter, 412 - 569 nm (FGB18)

Ø25 mm UG1 Colored Glass UV-Passing Filter, 325 - 385 nm (FGUV)

Ø25 mm KG5 Colored Glass Bandpass Filter, 330 - 665 nm  (FGS600)

1. **Neutral Density Filters (optional)**

Reflective Ø25 mm ND Filter, SM1-Threaded Mount, OD: 0.2 (ND02A)

Reflective Ø25 mm ND Filter, SM1-Threaded Mount, OD: 0.5: (ND05A)

1. **Tools & Miscellaneous**

5 hex keys of different sizes

Ball driver

Lens paper

Screws

Index cards

Rulers

Aluminum foil

Immersion oil

**Lab Module Instructions:**

**Optics 1: Optics basics, focal lengths of lenses, finite imaging, exploring the thin lens equation, building an finite optical system**

***Learning Objectives:***

-how lenses shape light, ray tracing, Snell’s law

-finite optical systems & how to use the Thin Lens Equation to find where real images of samples will focus

-finding the focal length of an unknown lens using the Thin Lens Equation & the concept of images at infinity

-magnification

-infinite optical systems & why they are useful

-becoming familiar with how to align optics, dealing with optical components & optical breadboarding

-origins of spherical aberrations

**Important take home point:** projecting images to infinity and focusing objects from infinity allows us to find the focal length of lenses

***Background:*** Be sure to have read the background material for this lab! We will be exploring lenses and how they shape light and images by building two optical setups: “finite” and “infinity-corrected” systems. You will see why they are called “finite” and “infinity-corrected” as we practice ray tracing, build the systems, and take some measurements.

***Experiments/Tasks***

**1] Set up your optical rail on the optics breadboard and assemble rail carriers**

dovetail rail

90°


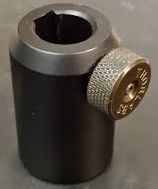

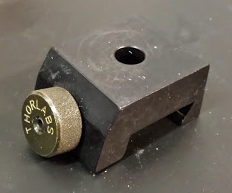
a.) Take your 24” dovetail optical rail and place it horizontally on your optical table leaving equal room on the left and right (see picture at right). Aim to have the rail as parallel to the edge of the breadboard as possible. Begin to screw in the rail by aligning the holes with the optical breadboard. It’s a good idea to first put in all the screws and twist each into the breadboard by hand a little bit. To ease stress on the rail and keep it aligned, use the ball screwdriver to slightly tighten each screw the same amount and cycle through to tighten all instead of fully tightening one and moving on to the next.

b.) Now assemble the rail carriers and post holders. Make 5 of these to start. You will use a screw through the bottom of the rail carrier (on the left) and attach it to the bottom of the post holder. We will then use posts which fit into the post holder to position optical elements on the rail.

Note that the post holder and post will give you control over the height of the optical element via the thumbscrew on the post holder. The thumbscrew on the rail carrier allows you to secure its position on the optical rail. Alignment in all directions (distances between elements, vertical alignment, etc) is very important in optics and that is why we are using the rail system.


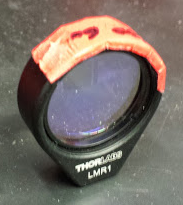


c.) Now we will mount the illumination LED source (which you have made in a previous electronics module), sample holder, and imaging screen.


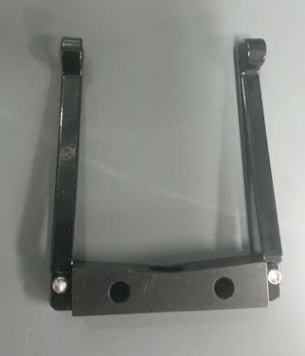
Attach the LED to a lens mount (see at left) and attach the lens mount to a post. Then put the post into a post holder already attached to a carrier.

We will be using a fixed filter holder as our stage/sample holder (see at right). Also mount this to a post and post holder + rail carrier.

Lastly, take an index card and tape it to a lens mount to serve as a white screen to project your images on. Screw this onto a post as well.

**2] Measure the focal length of a converging plano-convex lens**

Choose **any** plano-convex (PCX) lens (except for f = 25.4mm) in your kit and mount it on a post. Then put in the Stanford logo transparency into your stage and secure it to serve as your sample/object. You will be illuminating the sample using the LED light source. For now, the position of the light source is not important. Arrange your optical elements on the rail like this:


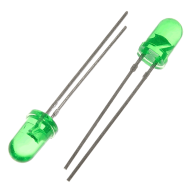


sample/object

LED light source

imaging screen

PC lens (aka objective)

Now change the position of the sample holder **OR** PC lens to focus the image of the imaging screen. A sample/object is in focus when **features at the center** are “crisp” on the imaging screen. You can test that you have true focus by moving the sample away from the point at which it was in focus and then past it to see the feature blur on both sides.

If you cannot bring the sample in focus at the imaging screen, increase the distance between the sample and PC lens as you may be at the focal point of the lens (we will cover this in more detail below).

***** Important terminology note**: the lens located just after the sample/object is called the **objective**. From this point on, we will use sample and object interchangeably and refer to lenses right after the object as objectives. Memorize this!*******

*Prove the focal length of your lens using the thin lens equation and distances to the object and imaging screen. What was the error between your calculation and what is indicated on the lens collar? What could be the source of that error?*

ANSWER:

Where and are distances to the object and real image.

*What is the magnification of your system?*

ANSWER: M = -S_image_/S_object_

*Using the system you have now, how would you increase the magnification of object by the single lens? Where would the real image be?*

ANSWER: You would move the object closer to the lens. The real image will now be further away from the back of the lens, but at a higher magnification.

Now move the other component (sample **OR** PC lens) and refocus your system.

*What is the magnification of your system now? Has the focal length of your lens changed?*

f

object

f

*What is the name for what you see on the imaging screen and why is it upside down? HINT: think back to your background reading. Ray trace the system below to demonstrate why the image is upside down.*

ANSWER: real image


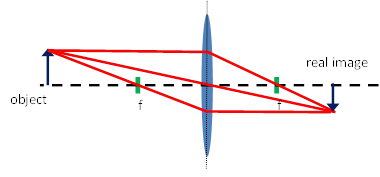


*How would you create a virtual image (one which is right-side-up and on the objective side of the lens)?*

ANSWER: put the object less than the focal distance away from the objective

Try creating virtual images with your system and if you have enough time ray trace the setup to convince yourself of how the virtual image is right-side-up.

**3] Origins of spherical aberrations & “which way should my lens face?”**

From the reading, spherical aberrations exist because spherical surfaces are “not thick enough” at the edges compared to the ideal curves and so light “bends back” prematurely before the focal point when focused from the edges of the lens.

You can examine spherical aberration by noting that when the center of your object is in focus, the edges may not be precisely in focus and vice versa.

Replace your lens with a very low focal point one (i.e., f = 25.4 mm). Get the system in focus. Note that the spherical aberrations with this lens are more pronounced than in a f = 100 mm lens. Also note that the radius of curvature for a f = 25.4 mm lens is much higher than f = 100 mm. As the radius of curvature of the lens increases, spherical aberration also increases.

So you might be wondering: these lenses are plano-convex, which means one side is curved and the other flat…which way do I turn the lens in my system?

Take the 25.4 mm PC lens and try both orientations.

Which one produces the most crisp image with least spherical aberration? We will discuss the physics behind the situation below, but first try out the experiment for yourself.

Think about how light rays would hit the surface of the lens. When we ray trace individual light rays, we assume lenses are thin and don’t worry much about how light interacts with lens surfaces in our high-level analysis. The following analysis below from [Khare & Swarup Engineering Physics](http://books.google.com/books?id=UfztNNnSFckC&pg=PA140&lpg=PA140&dq=spherical+aberration+which+side+of+the+lens&source=bl&ots=l7ZGUdGl22&sig=w7eisN8gF14YElaRwC6OxopWCtA&hl=en&sa=X&ei=oNy5UoDfC8jTsATq34CQDQ&ved=0CFgQ6AEwBQ#v=onepage&q&f=false) shows that spherical aberration is smallest (the distance between marginal and paraxial rays) when the curved side of the lens is facing the parallel incident beams of light (situation on the left). In our case, this means that the curved side of the lens should face either the largest object distance or an infinite conjugate (more on infinity later) because the further an object is, the more parallel the incident beams of light coming from it.


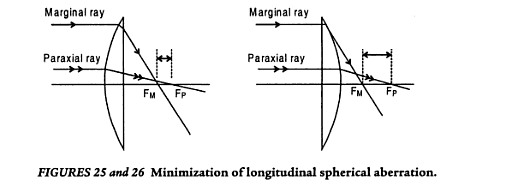


**4] Set up a finite optical system & find the real images of objects**

Now we will venture into two-lens finite optical systems. You will again be illuminating the sample using the LED light source and the position of this light source does not matter. Arrange optical elements on the rail like this (it is easiest to keep your setup from **2]**, move the imaging screen further away and add the 2^nd^ PC lens). The imaging screen should be as far back on the rail as possible:


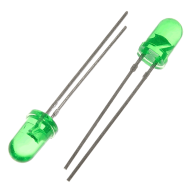


sample/object

LED light source

imaging screen

objective

PC lens 2

It is very important to have the elements all at the same height because we have multiple lenses in the system. This is most difficult to do with the light source. For the lenses, you can put them side by side on the rail directly touching and raise/lower the posts until they are at the same height.

The easiest way to get this system in focus is to first focus the first part (identical to **2]**) using only the objective. Using an index card, find the real image made by the objective. Then adjust the position of the 2^nd^ PC lens to get the image in focus on the imaging screen.

*Knowing how objects focus into real images using 1 lens, ray trace this system (using arbitrary distances between the object, objective, and PC lens 2).*

**

*Where are the real images in the system? For each real image, is it right-side-up or up-side-down? Use your constructed system for help in answering this question.*

ANSWER:

*How do you figure out the magnification of this system? Hint: dissect the system into two single lenses*

ANSWER: You can multiply the magnification of both lenses to find the final magnification of the system:

M = -S_image_/S_object_ since the object of the 2^nd^ PC lens is the real image of the first

Play around with this system and see if you can achieve a specific magnification you want. Notice that it requires quite a bit of moving around of parts.

**4] Focusing images to and from infinity**

In **2]** you used the Thin Lens Equation to figure out the focal length of a lens. Now we will lean an alternate technique to figure out the focal length of an unknown lens using infinity imaging.

We saw that if you put an object further from the focal point of the lens, we will form a real image a specific distance away from the focal point of the lens on the other side. If the object is closer than the focal point, a virtual image is formed on the object’s side of the lens.. side of the lens...rmed on the object'point of the lens on the other side. If the object is closer than the focal point, becau

*What happens when we put an object right at a lens’ focal length from the reading? Ray trace the scenario (hint: this was discussed in the reading):*

**

ANSWER: the object is projected to infinity

**

When an object is exactly at the focal point of the lens, it is projected to infinity. And the opposite is true, if the imaging plane is at the focal length of a lens, infinity is focused onto it.

So how does this help us get the focal length of a lens easily? Take one of your PC lenses and hold it above the bench. Try to focus the overhead light (which we will assume are at infinity in relation to your lens) on the table. Then measure how far above the table your lens was. Is it close to the focal length?

Now we’ll use objects far away to approximate infinity and position our optical elements precisely as it is difficult to measure focal lengths with mounted lenses (as you have already experienced). Start by leaving only a lens and imaging screen on your rail, use the thumbscrew to secure these components, and take your rail off of your optical breadboard. Try to focus a bright object from across the room or hallway (exit signs work well) onto the imaging screen and record the length between the lens and imaging screen.

Now you can also project objects to infinity. In place of the imaging screen, put your LED and draw on the LED with a marker a happy face ☺ (don’t worry, you can erase it later with ethanol). See how far you can project the happy face from the LED onto an index card by stepping further and further away from the optical rail. Then change the position of the LED relative to the lens and observe the difference in where the real image of the happy face forms.

*When projected to infinity, what is the orientation of the happy face? (right-side-up or upside-down?)*

ANSWER: images projected to infinity are right-side up and this can be shown through ray-tracing

At the end of this activity, make sure your PC lens is near the end of your rail and place the imaging screen back at the focal length of the lens (by focusing an object at infinity onto it). Now add the objective and sample to the left, do not worry about the length between the sample and objective. We are just setting up for next lab.

f_objective_


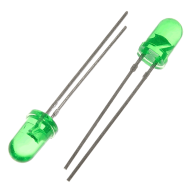


sample/object

LED light source

imaging screen

objective

PC lens 2 aka tube lens

f_tube_

**Optics 2: Setting up a camera focused at infinity, exploring magnification in a simple microscope, identifying infinity spaces and practicing ray tracing**

***Learning Objectives:***

-cameras & “imaging at infinity” using a tube lens

-properties of lenses, how focal lengths can help magnify or enlarge objects/light sources

-infinite optical systems & why they are useful

**Important take home point:** infinite optical systems are extremely useful & are the basis of all microscopy platforms. Cameras record real images by gathering photons which are converted to digital signals.

***Background:*** Be sure to have read the background material for this lab! We will be putting cameras into our infinite imaging systems and exploring properties of camera chips, magnification, and resolution (as determined by camera chip)

***Experiments/Tasks***

**1] Building an infinite optical system**

Modify your setup from lab 1 to resemble the following picture.

f_objective_


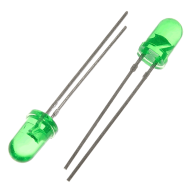


sample/object

LED light source

imaging screen

objective

PC lens 2 aka tube lens

f_tube_

Make sure your sample is approximately one focal length away from your objective. To get precise, you can present an object to the objective as far away as possible and project it onto an index card at the sample holder position. Move the stage position until you get the object to focus to place the stage right at the focal length of the objective.

***** important terminology note**: the lens located exactly one focal length away from your imaging screen is called the **tube lens**. It focuses objects at infinity onto the imaging screen. Memorize this!*******

*Ray trace this system before aligning the components to predict what will happen. Predict what you would see on an index card in the path between the objective and tube lens.*

**

ANSWER: In the “infinity space” between the objective and tube lens, you will not see a de-focused version of your object because none of the light rays converge.

**

What you have created between your objective and tube lens is infinity space. The huge benefit is that now you have lifted a design constraint in your system. You can make the infinity space as long or short as you want and maintain your image in focus (as long as the distance from the object to objective is f_objective_ and distance between tube lens and imaging screen is f_tube_).

Prove that you have infinity space between your objective and tube lens by sliding both the sample holder + objective (or tube lens + imaging screen) keeping the distance between them, but shortening/lengthening the distance in between two lenses. (see picture below) Make small adjustments to get your system in focus.

Alternatively, you can keep the objective + sample in place and move the tube lens, then adjust the imaging screen to get your image in focus. Play around with the system and complete the following table with some of your observations:

f1

object

f1

f2

f2

f1

object

f1

f2

f2

Infinity space

| **Distance between [mm] …** | | |
| --- | --- | --- |
| objective & tube lens | sample & objective | tube lens & imaging screen |
|  |  |  |
|  |  |  |
|  |  |  |
|  |  |  |
|  |  |  |

*Calculate the magnification of your infinite optical system.*

ANSWER: M = -f_tube lens_/f_objective lens_

So you can see that infinite optical systems are very useful as they allow for adjustable lengths in infinity spaces. In addition, anything you put into the light path in an infinity space doesn’t get focused on your imaging screen. Try it by putting a transparency with an image in the infinity space and see if it appears in your image.

*If you want to put a filter in your system, you should put it in at an infinity space. Why?*

ANSWER: So that particles on the filter surface don’t appear in your final image since objects in the infinity space are completely defocused.

By building an optical system with infinity space, we have just built the basics of a microscope! To see how our setup differs from a common light transmission microscope, look at this online tutorial. Note that the illumination path differs and that we will build it in Lab 3.

<http://zeiss-campus.magnet.fsu.edu/tutorials/basics/objectivemagnification/index.html>

**2] Prepare the CCD for imaging and focus the tube lens to infinity**

a.) Carefully crack open the webcam. Inside you will find a focusing lens in front of a bare CCD.

b.) Mount the camera onto the back of a 4 inch tube. Now make sure you have gloves on and place the f = 100 mm PC lens into the adjustable lens tube using the retaining ring (using the red tool in your box). Which way should the curved side of the lens face? Infinity space will be outside of the lens tube, so the curved surface should be pointing to toward it (flat side of lens into the tube facing the camera chip). Please ask the TAs/instructors if you have questions.

c.) Watch the animation on the following page on how to operate the adjustable tube and put in a lens: <http://www.thorlabs.us/newgrouppage9.cfm?objectgroup_id=4109>

d.) Plug in the USB from the camera into your laptop and get the Logitech software running to view the live webcam. Aim your tube to a feature far away from you (it may be a sign on the other side of the room, etc). Twist the adjustable part of the tube until the feature is in focus on your camera. Once you have found the focus, secure the retaining ring on the adjustable tube to secure the position. You now have focused your camera to infinity!

e.) If you have time and it’s not raining outside, you can venture outside with your laptop and tube lens + camera to find an object which more accurately resembles an object at infinity. Try focusing a tree or building far away onto your camera.

**3] Mount your camera on the optical rail**

You probably left your system from last time in a configuration like this:

f_objective_


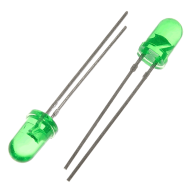


sample/object

LED light source

imaging screen

objective

PC lens 2 aka tube lens

f_tube_

*Since you have now focused your camera at infinity and it captures images, which two elements will you remove and replace? Ray trace the system*

**

ANSWER: The second lens of the system will be replaced by the tube lens and imaging screen is now the camera CCD.

**


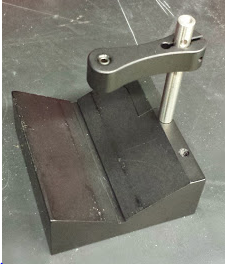
Place your tube lens + tube + camera into the system using the large clamp attached to a post and rail carrier, as usual. Make sure to relieve strain of the electrical wires by clamping down the USB cable to the breadboard so accidental tugs on the wire will not shift or damage the detector.

**4] Capture images from your infinity system**

Make any adjustments to your system (objective/sample) to get the image focused on the camera chip. Verify again that infinity space exists between your objective and tube lens.

Now capture an image of your sample (transparency with Stanford logo) onto your camera chip.

*Since we ripped out the CCD out of a commercial webcam, we don’t know the precise properties of the camera chip. How can we figure out the specs of the chip given a ruler, our sample, and the image you just captured? HINT: you can use ImageJ/Fiji to figure out how many pixels make up an image. *Describe what you would do, you don’t have to follow through with the actual measurement using this sample**

ANSWER: Perform the calculations for pixel resolution and field of view backwards. After saving the image, import it to ImageJ and determine the number of pixels in the image in both dimensions. Then measure on the actual sample the distance between features in the saved image, correct by the magnification, and determine the microns per pixel which were recorded.

The process using an un-calibrated sample is quite tedious. So we will use a resolution target called the US Air Force 1951 target. This target is chrome on glass (chrome blocks out all light so the contrast is very good). There is a series of small and smaller 3-bar test patterns on the plate. The smallest set of bars you can resolve gives you the resolution of your system. The resolution is 1/(line spacing) so if you can resolve 8 line pairs per mm, then you have a resolution of 1/8 mm = 0.125 mm. Because we know the exact size of each marker on the target, it becomes much easier to back-calculate the field of view of the camera and resolution of each pixel.

**group**

**elements**

How can you tell if bars are resolved? We will use quantitative analysis for this using ImageJ and the plot profile tool.

Here’s a quick primer on how to do that in Fiji/ImageJ:

| 1.) Drag your image to Fiji to open it and make sure it is a greyscale image (pick the highest bit-rate possible) by selecting Image > Type > 32-bit | 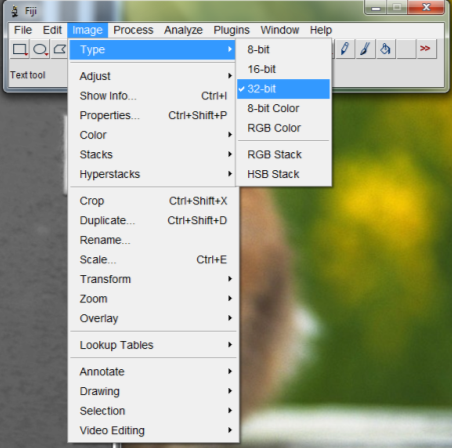 | 2.) Invert the image so that white becomes black and vice versa by selecting Edit > Invert | 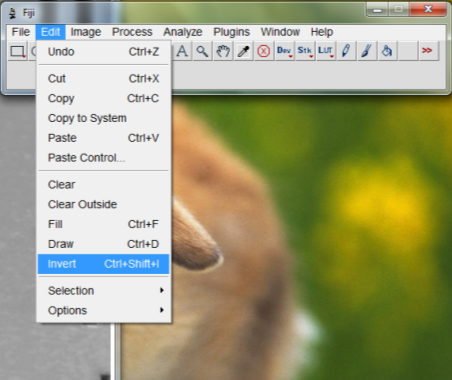 |
| --- | --- | --- | --- |
| 3.) Draw a line through the smallest elements you are able to distinguish. | 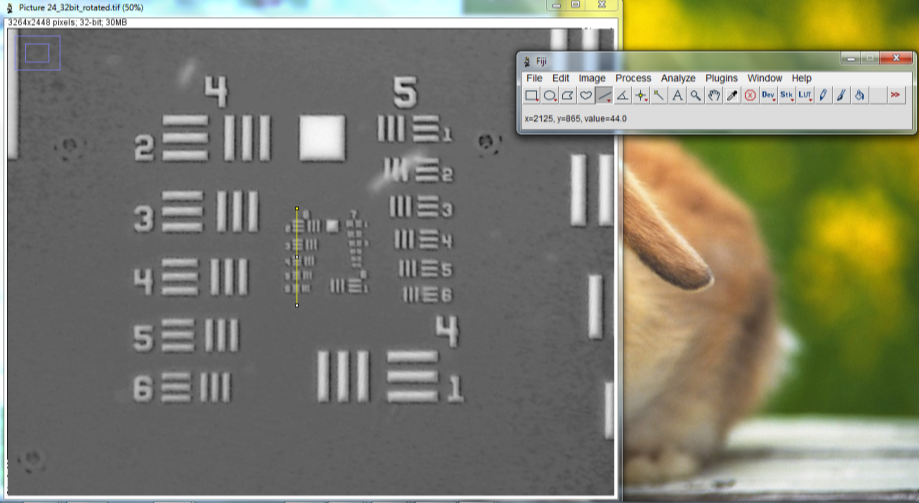 | | |
| 4.) Select the plot profile function and get the intensity plot of the features. | 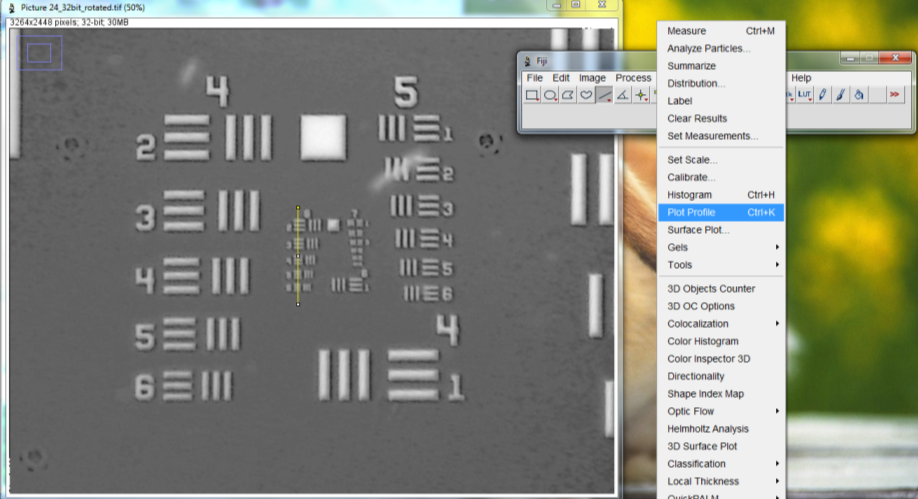 | | |
| 5.) Identify the peaks which are the last ones ~20% different in intensity from each other. | 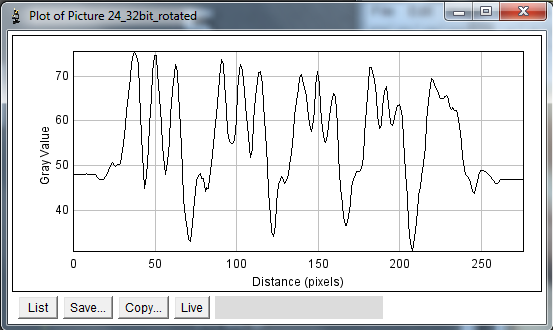In this case the very last peaks are not resolved and so the limit of our system is: Group 6, element 5, which corresponds to 102.0 lines/mm so the resolution of this system is 1/102 mm = 0.0098 mm = 9.8 microns | | |

So let’s put in the resolution target. Put on gloves. Carefully insert the USAF target with the chrome side facing the tube lens (so that you can read the writing on the surface) into the sample holder. Take great care not to put torque on the target as this will crack it. Avoid scratching the target with the stage clips.

Now it’s time to determine to specs of your camera CCD chip using the resolution target. Take an image using your webcam, import it into Fiji and follow the steps above. What is the resolution of your system?

Since you know how many line pairs per mm are in each group/element, use one of them to determine the number of pixels in that bar. From that, calculate the resolution per pixel of your system.

**5] Change the magnification of your system to get the highest possible scenario**

You currently have a 100X lens installed as your tube lens and your objective was chosen at random by your group. Compute the magnification for each system to fill in the following chart. What is the maximum magnification you can achieve with your tube and objective lenses?

| F_tube_ | F_objective_ | Magnification = - f_tube_/f_obj_ |
| --- | --- | --- |
| 50 | 25.4 |  |
| 50 | 50 |  |
| 50 | 60 |  |
| 50 | 100 |  |
| 50 | 125 |  |
| 50 | 150 |  |
| 50 | 300 |  |
| 50 | 400 |  |
| 100 | 25.4 |  |
| 100 | 50 |  |
| 100 | 60 |  |
| 100 | 100 |  |
| 100 | 125 |  |
| 100 | 150 |  |
| 100 | 300 |  |
| 100 | 400 |  |
| 125 | 25.4 |  |
| 125 | 50 |  |
| 125 | 100 |  |
| 125 | 125 |  |
| 125 | 150 |  |
| 125 | 300 |  |
| 125 | 400 |  |

*If you are using a PC lens for the tube lens, why is it important to use a longer focal length lens? Why would it be not good to use a f = 25.4 mm lens for the tube lens?*

ANSWER: PC lenses with short focal length have high spherical aberrations and you want to limit those especially at the camera where you are recording the image.

From the above analysis, it is best to keep the tube lens a longer-length lens. A reasonable focal length is 100 mm, please use a 100 mm lens for your tube lens. Try 3 different lenses for the objective, analyze the collected images, and record your observations below. Use the pixel resolution you determined above to calculate the “measured magnification” using one of the bars of the AF target. Of course, in this analysis don’t use the objective you used to find the pixel resolution as you took that magnification as a given.

It’s a lot easier to change just the objective and keep the tube lens the same, but feel free to modify it if you want (you will have to refocus it to infinity). * The f = 60 mm lens below is actually an achromat, so it is a lot more precious than the other PC lenses. Be careful with it!!!*

| **Objective Lens, f =** | **Tube Lens, f =** | **Predicted Magnification**  **M = - f_tube_/f_obj_** | **Measured Magnification** | **Resolution** |
| --- | --- | --- | --- | --- |
|  | 100 |  |  |  |
|  | 100 |  |  |  |
|  | 100 |  |  |  |

**Optics 3:** Setup Kohler illumination, explore conjugate planes, end with a high magnification microscope setup (with Kohler)

***Learning Objectives:***

-critical vs. Kohler illumination

-conjugate planes & image formation

-steps of how to get a microscope into Kohler

-evaluate the conjugate planes for light source & object in Kohler setup

-why is Kohler useful?

**Important take home point:** Evenly dispersed, bright, glare-free illumination is key to optimum imaging with any optical setup. Kohler is recommended by all modern microscope manufacturers and should be second nature when you start brightfield, darkfield, and phase imaging on a microscope. Evaluating conjugate planes is important to troubleshooting.

***Background:*** Be sure to have read the background material for this lab! We will be modifying the illumination path of our infinite imaging system.

***Experiments/Tasks***

**1] Set up an infinite imaging system with critical illumination**

You should already have an infinite system set up without any lenses in the illumination portion (LED shining directly onto the sample). Add a lens after the LED source with focal length of 50 mm and an iris to serve as the field stop. Ensure all optical elements are at the same height. Follow the diagram below:

object

f1

f2

f2

objective

tube lens

LED source

f3

f3

f1

collector lens

object

f1

f2

f2

objective

tube lens

LED source

f3

f3

f1

field stop

Make sure your system is in focus. Take an image of your AF target and figure out the resolution of the system (using ImageJ processing).

*Can you change the intensity of a light source using collector lenses of different focal length?*

ANSWER: No, there will not be a significant change in light intensity based on focal length. A PC lens numerical aperture will change with focal length (given lens shape) but this will influence contrast achieved in the system more than intensity. Only by modifying lamp voltage or power can we achieve greater intensities.

Now play with field stop iris and take images of your sample. Compare what you see in the images. Is the resolution the same? Complete the following table:

| **Image Filename as saved on your computer** | **Field stop is …** | **Smallest element is … (lines per mm)** | **Resolution is … (1/lines per mm)** | **Uniformity (qualitative assessment)** |
| --- | --- | --- | --- | --- |
|  | 100% open |  |  |  |
|  | 75% open |  |  |  |
|  | 50% open |  |  |  |
|  | 25% open |  |  |  |
|  | Almost closed |  |  |  |

*Where are the conjugate planes in this system?*

ANSWER: This is an infinite system with critical illumination, so the LED source, object, and detector are all conjugate to each other.

**2] Set up Kohler illumination on your infinite optical system**

Follow the following steps to first get your optical elements in position on the optical rail. We will give you some specific lengths which will hopefully make your first time getting to Kohler a little easier.

Let’s briefly review conjugate planes:

*What planes are in conjugate with the illumination source in a Kohler system? Which planes are conjugate with the sample plane in Kohler?*

ANSWER: Illumination: light source, aperture stop, back focal plane of the objective

Sample: field stop, sample, camera

*What is the formula for magnification in a Kohler-illumination infinite imaging setup?*

ANSWER: Magnification is not affected by the type/source of illumination and is still M = - f_tube_/f_obj_

First make your optical rail look like the following diagram with all the elements at the same height. Use the table below to determine which focal length lenses to use.

LED source

collector

tube

object

objective

condenser

camera

field

stop

aperture

stop

| **Lens** | **f = [mm]** | **Lens Type** |
| --- | --- | --- |
| Collector | 50 | PC |
| Condenser | 50 | PC |
| Objective | 60 | Achromat |
| Tube lens | 100 | PC |

Now use your ruler to put the components at the following distances (note that the diagram is not to scale, distances given in millimeters):

LED source

collector

tube

object

objective

condenser

camera

field

stop

aperture

stop

100

95

70

60

130

100

With the lenses close to the appropriate locations for Kohler, you can now proceed with the protocol for how to achieve Kohler (identical to your Background Notes, except we start with step 4 as we have already done 1 – 3):

1. Put an illumination source on the edge of your rail and a sample in the middle. Turn on the illumination source.
2. Make sure your tube lens focuses a camera to infinity. Add the tube lens and camera on the opposite edge of your rail. Place the objective between the object and tube lens. Add the collector and condenser lenses as well as the field and aperture stops.
3. Adjust the height of all the optical elements to get the lenses at the same height as the tube lens + camera. It is most difficult to adjust the position of the light source, approximate for now.
4. Make sure all aperture stops are fully open. Makes sure the field stop is as close as possible to the back of the collector.
5. Place your tube lens + camera behind the objective. Focus your objective and sample to get a crisp image on the camera.
6. Put the condenser about a focal length away from the sample.
7. Moving the collector, focus the illumination source at the aperture stop. It is easier to focus the source if it has a particular feature, such as a lamp filament. You can also draw something on the surface, such as a dot/splotch/happy face.
8. Close down the field stop (carefully, do not force it or go too fast as the diaphragm of the iris will break!!!).
9. Moving the condenser, bring the field stop into focus at the sample (and camera, a conjugate plane). You should be able to see the edges of the iris clearly.
10. Make sure the illumination source is still in focus at the aperture stop. You may need to adjust the collector again.
11. Open the field stop enough to illuminate the sample without extra light. Do this by looking at your camera and open the field stop enough such that the edges of the field stop are barely visible on your imaging plane. Close down the aperture stop to optimize the contrast. The aperture stop usually has to be adjusted such that it allows transmission through ~70% of the back focal plane of the objective.

Verify that you have indeed achieved Kohler illumination by showing the setup to your TA/instructor and walking through the appropriate conjugate planes for the illumination and sample. You may have to iterate through the steps above multiple times to achieve Kohler and the height of your light source and lateral shifting of lenses may lead to difficulties in adjusting the condenser so you get your closed field stop to focus at the sample/camera planes. Be careful and patient.

Now you have the power to control both the field stop and aperture stop. Explore the effect of each on your image by completing the following chart. Save your images for later analysis.

| **Image Filename as saved on your computer** | **Field stop is …** | **Aperture stop is…** | **Smallest element is … (lines per mm)** | **Resolution is … (1/lines per mm)** | **Uniformity (qualitative assessment)** |
| --- | --- | --- | --- | --- | --- |
|  | 100% open | Adjusted such that the objective BFP is 75% open |  |  |  |
|  | 75% open | “ … “ |  |  |  |
|  | 50% open | “ … “ |  |  |  |
| **Image Filename as saved on your computer** | **Field stop is …** | **Aperture stop is…** | **Smallest element is … (lines per mm)** | **Resolution is … (1/lines per mm)** | **Uniformity (qualitative assessment)** |
|  | 25% open | “ … “ |  |  |  |
|  | Almost closed | “ … “ |  |  |  |
|  | 75% open | 100% open |  |  |  |
|  | 75% open | 75% open |  |  |  |
|  | 75% open | 50% open |  |  |  |
|  | 75% open | 25% open |  |  |  |
|  | 75% open | Almost closed |  |  |  |

*What does the aperture stop control?*

ANSWER: The angular distribution of light hitting the sample.

*How would you go about improving contrast in your system?*

ANSWER: Adjust the condenser aperture (making it smaller increases contrast but decreases resolution).

Record the lengths that you ended up with in your Kohler illumination setup in the figure below:

LED source

collector

tube

object

objective

condenser

camera

field

stop

aperture

stop

**3] Re-establishing Kohler**

Change out the objective to get a different magnification in your system and readjust it to get the microscope into Kohler.

*Is it easier to achieve Kohler in high magnification or low magnification systems? Can you predict the distances between elements before you start the process? Record your observations:*

Conditions of Kohler are actually said to be difficult to achieve at magnifications of 5X and below (all of our possible systems fall into this criteria). Microscope manufacturers advise to start putting a microscope into Kohler illumination alignment using the 10X objective. So in a microscope with 10X magnification setup, it should be significantly easier for you to achieve Kohler. We encourage you to try getting a commercial microscope into Kohler illumination next time you are around one!

**4] Why is Kohler useful?**

Compare the types of images you obtained using critical illumination with the ones which you got using Kohler-illumination.

How does the resolution change? What effects do the field and aperture stops produce on your images?

You can even experiment with different samples. We will make biological samples in a later lab, but if you have time you can ask your TA/instructor for how to isolate some of your own cells to evaluate how the uniformity of light across a sample impacts the resolution with which we can distinguish its features.

**5] Bring your infinity system into the highest possible magnification with Kohler illumination**

We advise to stick with the 100 mm tube lens but chose the objective to give you the highest magnification while still achieving Kohler.

**We have built a whole microscope! We are able to magnify samples, resolve features, generate contrast, and capture images!**

**Optics 4:** Quantitative microscopy, imaging the Point Spread Function, exploring resolution versus contrast, introduce dark field and oblique illumination

***Learning Objectives:***

-theory of resolution from perspective of Point Spread Function (PSF) & Airy disks

-tradeoffs between resolution & contrast

-how to practically measure the PSF of any optical system (using dispersed beads)

-distribution of high & low resolution light in the back focal plane of the objective (in Kohler)

-darkfield illumination

**Important take home points:** numerical aperture, not magnification, sets the smallest details you can resolve in an image. Increasing NA increases the amount of light collected by the lens, thereby increasing the brightness of the image & reducing contrast. By masking some of the low resolution light coming from the sample, we can increase contrast of transparent samples.

***Background:*** Be sure to have read the background material for this lab! We will be evaluating the resolution of our images using quantitative microscopy techniques and measuring the point spread function of our setups. We will also explore dark field illumination and what kinds of samples it is best suited for.

***Experiments/Tasks***

**1] Resolution comes from two sources: optical & imaging (camera)**

From last lab task 2, calculate the theoretical optical Rayleigh resolution & imaging (camera) resolution of your system and compare your data to measured resolution from the AF target. Comment on any patterns you notice as your field and aperture stop changed shape. The calculated camera resolution should remain constant throughout your analysis. Use the pixel pitch (μm / pixel) dimensions you derived in a previous lab.

We can use 500 nm for the wavelength since we are using green light LEDs for illumination. To be more precise, we could have measured the spectrum of our LED to find the appropriate wavelength.

*HINT for calculating Rayleigh resolution: d = 1.22*λ/(condenser NA + objective NA)

You will need the NA of your lenses. Remember, NA ≈ n*(D/2f) where n is the refractive index (assume we are working in air, so n ≈ 1), f is the focal length, and D is the diameter of the lens. Keep in mind that D is the entrance pupil diameter of the lens, so if you use a diaphragm to restrict light into your lens, this becomes the effective D.

| **Image Filename as saved on your computer** | **Field stop is …** | **Aperture stop is…** | **Measured resolution of the whole optical system is … (1/lines per mm)** | **Rayleigh Resolution (calculate)** | **Your qualitative observations** |
| --- | --- | --- | --- | --- | --- |
|  | 75% open | 100% open |  |  |  |
|  | 75% open | 75% open |  |  |  |
|  | 75% open | 50% open |  |  |  |
|  | 75% open | 25% open |  |  |  |
|  | 75% open | Almost closed |  |  |  |

**2] Modify the back focal plane of your objective for darkfield microscopy**

We will now modify the back focal plane of our objective to see what kinds of effects it has on our sample.

First, replace your sample with the AF target. Make sure your microscope is aligned in Kohler and that an aperture for back focal plane of the objective is set up at the correct place.

*How do you check that your aperture is located right at the back focal plane of the objective?*

ANSWER: You should be able to visualize the field stop at the back focal plane of the objective as it is conjugate to the objective BFP.

Make your aperture stop as small as you can and set the BFP of the objective to 8 mm. Take an image of your sample and save it. Continue and make the BFP 6 mm, 4 mm, and 2 mm. Save images each time.

Open the BFP to 6 mm again. Block the middle of the objective BFP. Aluminum foil works well. It’s good to secure it on holder so you can more precisely adjust it. Your image should look very interesting and close to darkfield illumination. The background should be dark and the sample should show up as white elements.

Now we will try to create a similar image to the darkfield technique using image subtraction. In ImageJ, open the BFP = 6 mm and BFP = 1 mm images.

Use the box tool and outline a large box in a bright area of the BFP = 6 mm image. Select Analyze > Histogram and read off the mean pixel value for the bright area. Write it here:

Now, move on to the BFP = 1 mm image and use the box tool to outline a large bright area. Select Analyze > Histogram and read off the mean pixel value for the bright area. Write it here:

**What is the ratio BFP = 6mm / BFP = 1mm in pixel intensity? Write it here:**


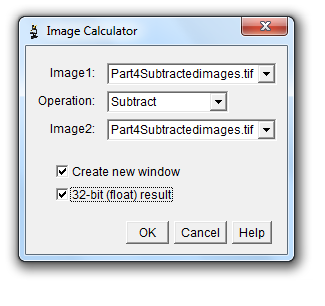


Now switch back to the BFP = 1mm mage. Select Process > Math > Multiply and input the ratio you just calculated.

We will now subtract the two images. Select Process > Image Calculator to subtract the two images, use the 32-bit mode and select “Create a New Window.” Take the absolute value of the result by selecting Process > Math > Abs

*Compare the images you generated when you physically changed the objective BFP to achieve a darkfield image and the image processing from subtracting images taken with different objective BFPs.*

*Why did subtracting these two images generated with BFP = 1 mm and BFP = 6 mm give you results similar to your darkfield technique?*

*What did we do when we blocked out the middle of the objective BFP with the tin foil mask? Explain terms of NA and resolution.*

ANSWER: When we decrease the size of the aperture at the objective BFP and take the BFP = 1mm image, the NA decreases so resolution of the image declines (since resolution = (1.22*λ)/(NAobj + NAcond)). When we subtract the image resulting from the low NA (BFP = 1 mm) and subtract that from the image captured using high NA (BFP = 6mm), we .get rid of the light rays in the middle of the objective BFP which are giving us low resolution, and keep only the high resolution light rays that are at the edges of the BFP. This is exactly the same thing as physically changing the objective BFP by putting a mask there to block the low resolution light from hitting our detector.

*Complete this sentence: _____ resolution image information appears in the outer part of the objective back focal plane.*

ANSWER: High resolution.

Blocking the center of the BFP is equivalent to subtracting a low resolution image from the total image

**3] Explore oblique illumination microscopy with dark field imaging**

Take out your mask and open the objective BFP back to 8 mm. Keep the aperture stop small.

Use aluminum foil and tape to mask off the aperture stop such half of it is covered (see diagram at right). Also use some aluminum foil to block the objective BFP just enough so that the image is mostly dark. Take a picture of your image.

*Describe what features you see on your sample?*

ANSWER: When the mask is horizontal, only the vertical features of the image should be highlighted.

*Make a prediction about which features of the sample will be highlighted if you turn the mask to block off vertical components of the aperture stop.*

**

ANSWER: When the mask is vertical, only the horizontal features of the image should be highlighted.

Try out the experiment by rotating your aperture stop mask by 90 degrees.

*How do your images taken with oblique and darkfield illumination differ? Where in the objective BFP is information about vertical and horizontal features stored?*

ANSWER: Vertical edges were highlighted in oblique illumination when the aperture stop mask was horizontal (and vice versa). These observations imply that the vertical edges and horizontal edges of the image are distributed in an opposing way on the objective BFP. Resolution of horizontal and vertical edges is contained in different planes of the BFP as changing the axis of illumination in oblique darkfield results in resolution of different parts of the USAF target

What if you make a strip across the aperture stop like the figure to the right. Which features in your sample are illuminated?

**4] Prepare a biological sample to explore the benefits of dark field microscopy (skip if you are running behind)**

We will now prepare a sample of your own buccal (cheek epithelia) cells!

Prepare a clean microscopy slide, cover slip, and nail polish.

Take a clean pipette tip and scrape the side of your cheek gently for a couple of minutes. Dab the saliva + cells on the clean slide. Pop any bubbles that form using the pipette tip. Gently put the cover slip on top and seal the edges with nail polish. Wait 5 – 10 minutes for the nail polish to dry.

Image your sample using brightfield illumination and dark field. Try oblique illumination combined with dark field. Which features are easier to resolve with each illumination and imaging method? Take some pictures and think of other samples which would be more compatible with either brightfield or darkfield.

**5] Setup to measure the Point Spread Function of your system**

a.) You will need to change your scope such that the stage, objective, and tube lens + camera sits vertically while the illumination optics (light source, collector, condenser) are horizontal. Please see the teaching rig in the back of the room, take notes, and replicate at your own setup.

b.) We will now use an actual microscope objective ([Edmund DIN 10X NA=0.25, focal length = 16.6mm](http://www.edmundoptics.com/microscopy/finite-conjugate-objectives/international-standard-microscope-objectives/36-132)) in place of our objective lens to increase the magnification of our system. Please use the objective holder (ask TAs/instructor) to mount your objective. Objectives contain many lenses to correct for spherical and chromatic aberrations but you can treat them as a single optical element.

c.) Ensure that your system is in Kohler illumination by going through the steps we did in lab 3. The key is to have the light source defocused at the sample (before it hits the objective).

d.) Prepare your slide by first drawing a small marker line on one side of the slide to make focusing easier. Then add microbeads (0.5 μm diameter spheres) by tapping the cap of the spheres on the stage to the same side of the slide by carefully opening the bottle and tapping the cap once on the slide to distribute a small amount of beads. They should stick to the slide, or you can put a coverslip on top and attach it with nail polish.

e.) Place your slide into the microscope, ensure your system is in focus. Use the marker line to help you.

f.) Turn the room lights off.

g.) Place an iris at the objective’s back focal plane. Set the back focal plane diameter to the largest (~8 mm).

h.) Open the aperture stop all the way.

i.) Zoom into your live camera screen on your laptop and scan around the sample screen until you find an isolated bead. Your bead is a point source of light because it’s acting like a pinhole. Make sure you are in good focus. You should be able to see one of the rings of the Airy disk.

j.) Adjust your exposure and take multiple frames to average out the background noise.

k.) Repeat the protocol above starting from step e with the objective BFP set at 3.5 mm wide. How is the PSF different?

l.) Repeat the protocol above starting from step e with the objective BFP set at 1 mm wide. How is the PSF different?

We hope the protocol above was successful. If not, we will provide you with images of the point spread function (PSF) for another system and you can analyze them in the step below. You can always quickly measure the PSF of your system using microscale beads as pinholes.

**6] Analyze the PSF of your system**

Open the PSF taken with fully open BFP in ImageJ. Crop out the PSF point leaving some space on all sides. Save as a new file (.tif format).

| 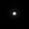 | 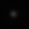 | 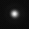 |
| --- | --- | --- |
| BFP dia = 8.0 mm | BFP dia = 3.5 mm | BFP dia = 1 mm |


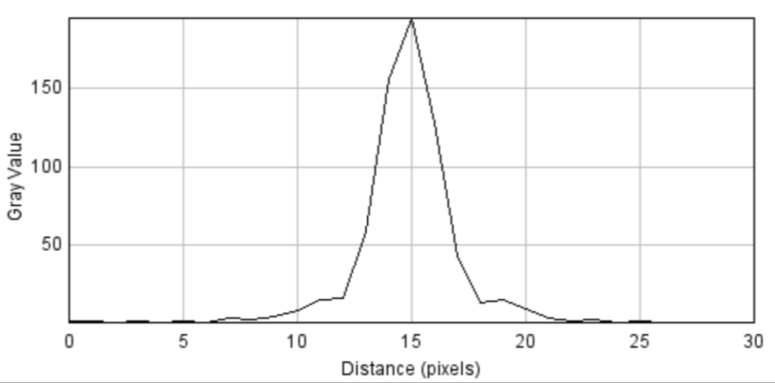
Zoom into the image. Draw a line through the middle of the spot (keep it horizontal by holding shift). Select Analyze > Plot Profile of the line to get the intensity throughout the point. It should look something like the figure on the right.

Now generate a surface plot for your PSF by selecting Analyze > Surface Plot. Make the Polygon Multiplier 100, select Draw Wireframe and Draw Axis. You will get something like the figure below:


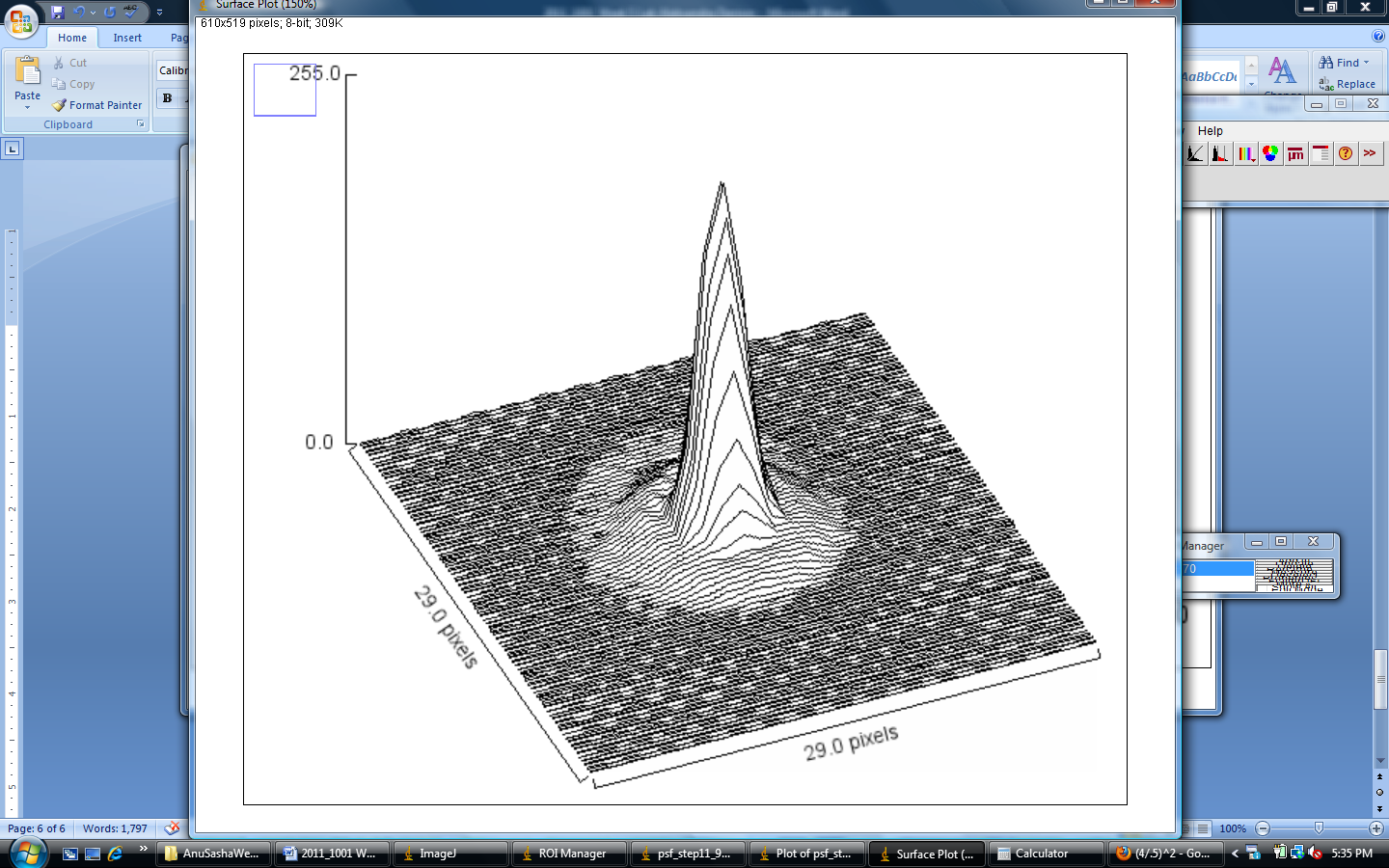

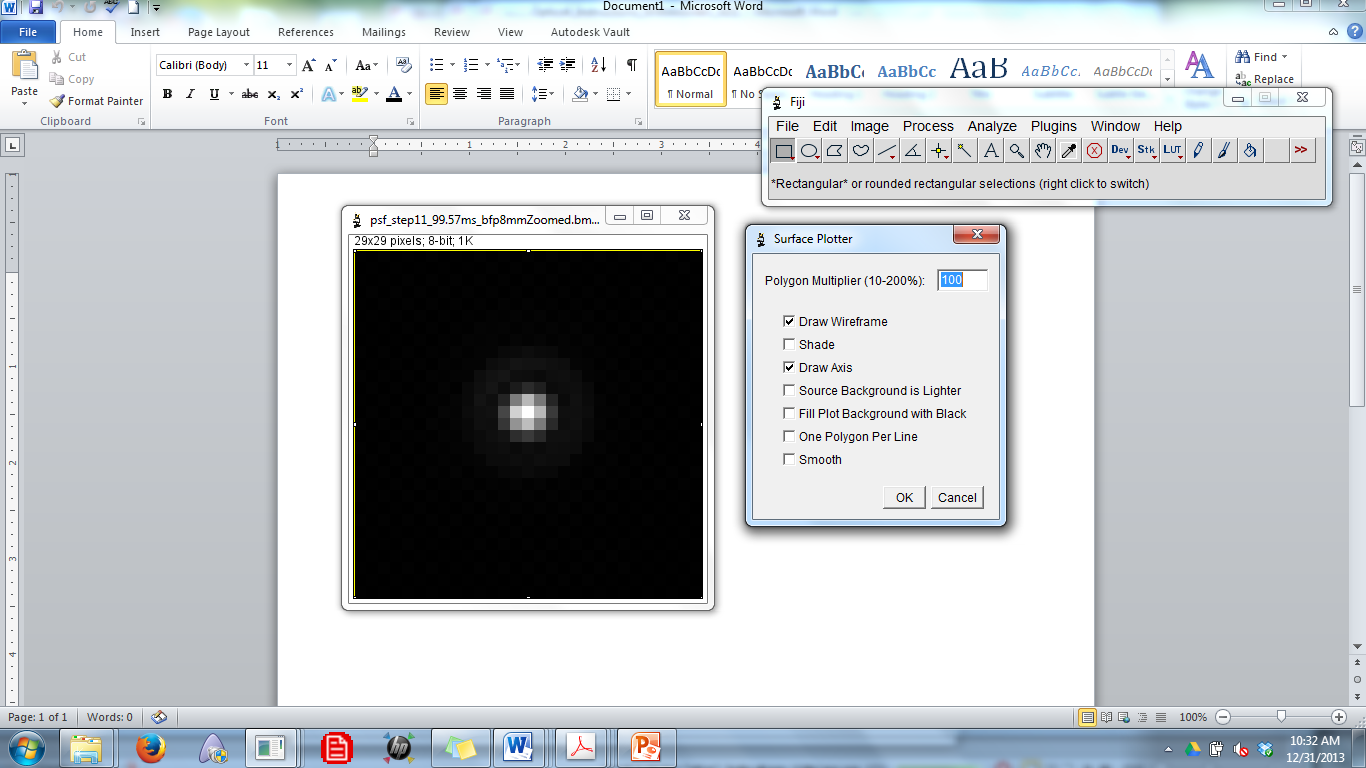


Save the plot profile and surface plot.

*What is the diameter of the Airy disk in your PSF images? Give the measurement in pixels and microns. Hint: Use the line scan.*

ANSWER: Measure the diameter of the 1^st^ ring of the PSF. Use the conversion factor of the detector (pixel/mm) and magnification system to find the size of the PSF in microns.

*What do you expect the diameter of the Airy disk to be in your system? Calculate the smallest resolvable distance (Airy disk diameter) if you ignore the condenser NA contribution. Assume you use 500 nm illumination light.*

ANSWER: Assuming that students are using an objective with NA = 0.25, we use the smallest resolvable distance according to Rayleigh (do not account for the condenser):

d = 0.61*λ/NA

d = 0.61*λ/NA = 0.61*500 nm/0.25 = 1220 nm = 1.22 μm

*Measure the resolution of your system (using the AF target) and compare it with the value you just calculated for the Rayleigh resolution. What could be possible sources of discrepancies?*

*Now calculate how the Airy disk diameter changes as a function of the BFP diameter. Assume max BFP diameter is 8.3 mm, objective focal length is 16.6 mm, λ = 500 nm. Do not account for the condenser.*

| *BFP Diameter* | *Objective NA* | *Smallest resolvable distance (Rayleigh Limit) [μm]* |
| --- | --- | --- |
| 1 mm |  |  |
| 3.5 mm |  |  |
| 5 mm |  |  |
| 8 mm |  |  |
| 8.3 mm |  |  |

ANSWER: Assuming students use f = 16.6 mm:

| *BFP Diameter* | *Objective NA = D/2*f* | *Smallest resolvable distance (Rayleigh Limit) [μm]* |
| --- | --- | --- |
| 1 mm | 0.03 | 10.13 |
| 3.5 mm | 0.11 | 2.89 |
| 5 mm | 0.18 | 1.69 |
| 8 mm | 0.24 | 1.27 |
| 8.3 mm | 0.25 | 1.22 |

*Now take into account the NA of your condenser. You did not modify the condenser dimensions while gathering the PSF images (it stayed at 75% open). Measure the field stop opening to calculate the NA of the condenser. What do you expect the diameter of the Airy disk to be in your system taking both the objective and condenser into account? Assume you use 500 nm illumination light and that the condenser is a 50 mm lens, 0.75% open (max diameter 8 mm).*

| *Condenser NA* | *BFP Diameter* | *Objective NA* | *Smallest resolvable distance (Rayleigh Limit)* |
| --- | --- | --- | --- |
|  | 1 mm |  |  |
|  | 3.5 mm |  |  |
|  | 5 mm |  |  |
|  | 8 mm |  |  |
|  | 8.3 mm |  |  |

ANSWER: We use the formula for condenser and objective NA contribution to resolution:

d = 1.22*λ/(condenser NA + objective NA)

| *Condenser NA* | *BFP Diameter* | *Objective NA* | *Smallest resolvable distance (Rayleigh Limit)* |
| --- | --- | --- | --- |
| 0.75% open 8 mm = 6 mm  NA = D/2f  NA = 6 mm/(2*50 mm) = 0.06 | 1 mm | 0.03 | 6.77 |
|  | 3.5 mm | 0.11 | 3.69 |
|  | 5 mm | 0.18 | 2.53 |
|  | 8 mm | 0.24 | 2.03 |
|  | 8.3 mm | 0.25 | 1.97 |

**Optics 5:** Exploring Brownian motion & measuring diffusivity

***Learning Objectives:***

-design an experiment and conduct measurements on own custom built optical setup

-measure diffusivity of a particle from its coordinates of displacement vs time

-use the Einstein relation to derive the size of a particle from diffusivity (and vice versa)

***Background:*** Be sure to have read the background material for this lab to understand the origins of the Stokes-Einstein relation and laws of diffusion!

*This lab is a write-up. There will be no questions during the lab, you are to follow the outline on the last page to hand in a written lab report due 1 week from the lab.* We will give you less direct guidance on this lab in the directions as you are now familiar with building, calibrating, and characterizing optical systems.

***Experiments/Tasks***

**1] Observe microbeads undergoing Brownian motion in solution to measure the diffusivity constant**

Remember that you will need to collect data about displacement of the microbeads versus time. Thus, you will need to know how displacement of your sample (in microns) correlates to displacement in pixels that you see on images on your computer screen from the camera. The easiest way to do this is to calibrate your system first by imaging a bar of your AF target, counting how many pixels correlate to that feature, and determining the pixel pitch of your camera. You will be changing objectives, so it’s easier to find the pixel pitch of the camera using the 10X objective and then multiply by different magnifications later on to find how many pixels correlate to a micron in your images. Make sure to take note of your measurements and the pixel pitch you calculate.

You will need to make a ‘flow cell’ to create your bead sample. Take a 1 inch length of double-sided tape and cut it in half length-wise. Make a window from the tape on a clean glass slide. Deposit 20 microliters of diluted bead solution into the flow-cell. Place a coverslip on top and seal the channel with nail polish. Let it dry for 5 minutes.

Place your sample on the stage such that the coverslip is closest to the objective. First, use a 10X objective to get your sample in focus. Then replace the 10X with a 100X objective (oil immersion). Deposit 1 drop of immersion oil on the objective. Put the glass slide back onto the slide holder with the coverslip down. The coverslip should be touching the oil on the objective.

Focus your sample by adjusting the z stage. Once you have the beads in focus, take a 5 minute movie of the beads moving around using the webcam software. If the software gives you trouble, try taking ‘snapshots’ of your setup every 30 seconds. Snapshots will also be easier to analyze later with ImageJ. You will use this movie/a stack of your snapshots (position vs displacement data) to derive the diffusivity of your beads in solution.

You can trace the location of the beads in a simple way using a transparency taped to your computer monitor to track the displacement of a single bead over 30 second intervals. Or you can use ImageJ to open your movie file and find the X and Y pixel locations of your bead. You can later convert the pixel locations to microns that the beads moved in the sample.

Tracking beads: This can either be done manually (not recommended) or using ImageJ’s plugins for tracking beads ([MultiTracker](http://rsbweb.nih.gov/ij/plugins/multitracker.html)). You can also use the built-in particle analysis steps in ImageJ. Select ‘set measurements’ in the Analyze menu, check ’center of mass,’ then change the image threshold (image>adjust>threshold) to select a dark background so that only the bright center of a bead is selected. Select ‘apply’ and then ‘analyze particles’ from the Analyze menu. Check ‘display results’ and save the results so that you can open them in a spreadsheet. The results should give you the X and Y positions (in pixels) of your particle over the image stack.

Make sure you track at least 3 beads in your sample (30 second intervals, 5 minute movie). You should then have 33 sample points total (make a spreadsheet like the one below). Calculate the total displacement (in microns) of each bead (abs[starting point – ending point]) in two directions (some Pythagorean relations will help here).

|  | **Bead (x and y coordinates)** | | |
| --- | --- | --- | --- |
| **Timepoint** | **1** | **2** | **3** |
| 0 sec |  |  |  |
| 30 sec |  |  |  |
| 60 sec |  |  |  |
| --- |  |  |  |

Calculate the diffusion coefficient based on these measurements (see background reading for relevant formulas). Then calculate the expected diffusion coefficient according to Stokes-Einstein. Compare the values.

Please remember that your write up for this lab is due in a week. Follow the instructions below.

**Sections to include:**

Introduction: The introduction includes any background needed to understand the work. It also includes motivation for the work.

Methods: The methods section describes what was done in sufficient detail to replicate the experiment.

Results: The results section reports the outcomes of the experiments.

Discussion: The discussion section describes what the results mean. Commonly addressed topics include: comparison to literature values, discussion of errors, implications, and future work.

References: This section contains any other relevant documents which were important to the report.

**General guidelines and points of style:**

| - type your lab reports   - include a title  - include your partner's name  - include a date  - write in sentences, not lists   - turn in lab instruction worksheet with the report, but do not rely on it as part of the report. | - use an equation editor to format all equations   - include equations and calculations  - include labels on all plots  - include units on all values   - use a reasonable number of significant figures  - include the data collected in the lab  - when possible organize and format data into easy to understand formats, such as a plot or table  - space out large blocks of text into paragraphs |
| --- | --- |

**Optics 5:** Error analysis for exploring Brownian motion & measuring diffusivity

The objective of this lab and lab report is to show how you measured diffusivity with a confidence interval of error (report D = X ± Y m^2^/s). Compare diffusivity to the theoretical diffusion constant based on the Stokes-Einstein relation. Can you tell if the error was due to sample variation (systematic) or from measurements (random) or due to human mistakes?

You might be wondering, how do I calculate the error in my measurement? There are several ways to do this, it’s up to you which one you choose (as long as you give a logical explanation). I will detail some methods below (you are not limited to these!). Use the error analysis textbook which we gave you at the beginning of term.

**1] Standard Deviation**

This will give you a measure of the dispersion of data around the mean of that sample. It doesn’t tell you about the population distribution (remember that your samples are just ‘tastes’ of the true population and it’s the true population you always care about).


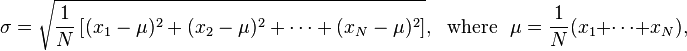


N is the number of samples, μ is the sample average, xn are the values which make up your sample

**2] Standard Error of the Mean**

The standard error will give you the distance between your sample mean and population mean. It will tell you how close your samples came to your ‘true population’ values. If you are reporting the standard error (standard deviation) of your sampling distribution, you assume the data is normally distributed (follows the Gaussian distribution bell curve). Thus you need to ensure that you pass these criteria: measurements you are putting into your standard deviation formula are normally distributed and independent from each other.

You can ensure normality of your sample distribution in two ways (info here: <http://stattrek.com/sampling/sampling-distribution.aspx>):

1) sample size is ‘large enough’ so that the distribution will be nearly normal due to the central limit theorem. Many statisticians advise a sample size of 30 as large enough.

2) make a quantile-quantile (q-q) plot to check the normality of a distribution. q-q plots are super useful if your sample size is not quite 30, but you want to show that it is normally distributed to use standard error analysis. In general, you compute the theoretically expected value for each data point based on the distribution in question and compare it to the value you measured. If the data follows the distribution you assumed (Gaussian), then your q-q plot will show a straight line. See here for how to make it step-by-step:

<http://onlinestatbook.com/2/advanced_graphs/q-q_plots.html>

Only when you have verified that your sample distribution is normal can you apply the standard error of the mean (standard deviation) measurements. Note that with increasing sample size n, your standard error of the mean decreases.


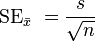


s is the sample standard deviation (sample-based estimate of the standard deviation of the population)

n is the number of sample points

**3] Uncertainty Analysis**

This is a very cool and super useful method (put it in your engineering toolbox!) to investigate which variables in your system can lead to the greatest uncertainty in measurements. You could calculate the uncertainty of your analysis for a particular variable (in our case, diffusivity) and relate how you expect the standard deviation to be (from uncertainty value) with the sample standard deviation. Uncertainty analysis then lets you also see which one of your variables may be the leading cause of your error (a great device design tool).

Ok, here’s how you go about it (refer to Holman for a good overview):

1.) Make a governing equation for your system. We are using one example where the calculation for electric power is as follows:

$$P=EI$$

Where E and I are measured as:

E = 100V ± 2V

I = 10A ± 0.2A

2.) Determine which variable you want the uncertainty of…Let’s say Power for this case

3.) Take partial derivatives of the equation with respect to each variable:

$$\frac{\partial P}{\partial E}=I$$

$$\frac{\partial P}{\partial I}=E$$

4.) Plug into the uncertainty relation where $w_{n}$is the uncertainty in the n variable:

$${w_{p}}^{2}={(\frac{\partial P}{\partial E}w_{E})}^{2}+{(\frac{\partial P}{\partial I}w_{I})}^{2}$$

5.) Now put in your differentials into the equation:

$${w_{p}}^{2}={(Iw_{E})}^{2}+{(Ew_{I})}^{2}$$

6.) Determine the individual uncertainties of your measurements on the right hand side of the equation.

For example, $w_{E}$ is 2V from above because we know our E measurement can be ±2V

$w_{I}$is 0.2A from above

The nominal values for E and I are also given above

7.) Plug everything in to find the expected uncertainty in power given your individual measurement uncertainties!

$${w_{p}}^{2}={(Iw_{E})}^{2}+{(Ew_{I})}^{2}={(10A*2V)}^{2}+ {(100V*0.2A)}^{2}$$

$${w_{p}}^{2}=20W^{2}+20W^{2}=40W^{2}$$

$$w_{p}=6.32W$$

So the expected uncertainty in our measurement is ± 6.32 W
