# Supplementary material for: A Biotic Game Design Project for Integrated Life Science and Engineering Education
Source: PLoS Biol. 2015 Mar 25;13(3):e1002110. doi: 10.1371/journal.pbio.1002110 (PMC4373802; doi:10.1371/journal.pbio.1002110)
Supplement: S22 Text — (PDF) [file pbio.1002110.s022.pdf]

Microfluidics

Problem Set

Due: Date at the end of lab.

Text: Posted readings (In 'MicrofluidicsReadings' folder on coursework)

Estimated reading time: 1 hr for Core reading, additional up to 3+ hrs for optional reading

Learning Goals

- Develop an understanding of the origin of the field
- Develop a sense of what 'microfluidics' means
- Become familiar with considerations of working on small things

<u>CORE READING (Posted Papers)</u>
The origins and future of microfluidics
There's plenty of room at the bottom
Life at Low Reynolds Numbers

<u>OPTIONAL READING (Posted Papers)</u>
A passive pumping method for microfluidic devices
Microfluidics: Fluid physics at the nanoliter scale
Microfluidics-Theoretical Aspects

<u>PROBLEMS</u>

Problem 1: Thinking back to the electronics unit, what are the fluidic equivalents of:

- Resistance
- Voltage
- Current

Problem 2: approximate the surface area to volume ratio for a whale (10 m), human (0.5 m), paramecium (100 μm), *E. coli* (1 μm) (approximate each organism as a cube)

Problem 3: List three special considerations for engineers working at the microscale.

Problem 4: Suggest two uses for microfluidic devices in bioengineering/biotechnology.

Microfluidics

<u>Laboratory Instructions</u>

Date

Objective

- Develop hands on intuition for microscale fluid behavior
- Learn a simple method for rapid microfluidic device fabrication
- Solidify electronics concepts with fluidic analogies
- Learn a surface treatment
- Work with flexible polymers
- Use a working valve device
- Discover the microfluidics of wine

Background

The field of microfluidics continues to grow at a rapid pace. Most techniques used in research involve the process of photolithography to create a mold and soft lithography to cast polydimethylsiloxane (PDMS) devices off it. Here you will be introduced to a rapid prototyping technique which will allow you to design, create and test several devices in the span of a few hours using PDMS and direct cutting. As you go through the lab, keep in mind ways that you can use this fabrication method in your final project. Additionally you will have the chance to try a working device with integrated valves, and learn about the microfluidics of wine.

Parts List/Materials

- **Supply list:**
- 1 dish PDMS sheet “15” (Sylgard 184 Dow Corning)
- 1 dish PDMS sheet “40” (Sylgard 184 Dow Corning)
- 1 Scalpel (Amazon)
- 3 glass slides (Fischer Sci)
- 2 dead end connectors (Value Plastics)
- 2 syringes (Becton Dickinson)
- 2 turquoise blunt tipped needles (23 G Brico)
- 2 three way stopcocks (Cole Palmer)
- 3 small metal tubes (23 G Instech Solomon)
- 3 1 foot lengths of tubing (Tygon AAQ04103, Murdock industrial)
- 1 waste petri dish (Fischer Sci)
- 1 squeeze bulb pipette (Lab Depot)

- 1 roll scotch tape (3M)
- 1 sheet white paper
- Several paper towels
- 1 wine glass
- Food coloring (1 set /two groups)

Shared by all:

- Hole punches (TedPella/HarisUnicrore or)
- Plasma wands (Electro Technic, BD-20AC Laboratory Corona Treater, 115V)

Planning Phase (15 min)

- Please read the instructions, gather materials, and prepare your notebook including a time plan.
- The focus of this lab is on learning a technique rather than making a scientific measurement. It will be sufficient to largely refer to these instructions and keep the writing in your lab notebook to a minimum.
- For your notebooks, please draw and label the fluidic control setup, and draw the device for each section.

Experiments/Tasks Execution Phase (165 min)

Note: we will stagger groups for demonstration of tears of wine and the valve device

Tips:

Avoid debris!

Use tape to clean devices

Do not touch surfaces that will be bonded!

Make sure the cores are removed from the hole punches!

Use your PDMS economically-you have a limited amount

<u>Part I: Make a T intersection (1 hour)</u>

From the thin layer, cut out the outline of the following shape:

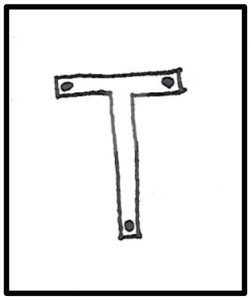

In the thick layer punch holes corresponding to the dots. Use the 0.75 mm (green hole punch) located at the hole punching station.

Plasma oxidize a glass slide, and each layer at the plasma station.

Assemble the layers together.

Place your assembled device on a hot plate at 100 ºC for 10 min to complete the bonding.

Copy the demonstration fluidic control setup using the tubing, stopcocks, syringes, connectors, and small metal tubes. Fill the reservoirs with tap water, and color one reservoir blue and one yellow (2 drops blue and 4 drops yellow should suffice)

Connect your device to the fluidic control apparatus. Flow both colors and observe. What is this effect called? Notice the effect of raising and lowering one or both reservoirs.

Show a member of the teaching staff that your setup works. As always, ask if you have questions!

_____TA check

<u>Part II: Make a Micromixer (30 min)</u>

Use the same method as in Part I to create a micromixer device of the following design:

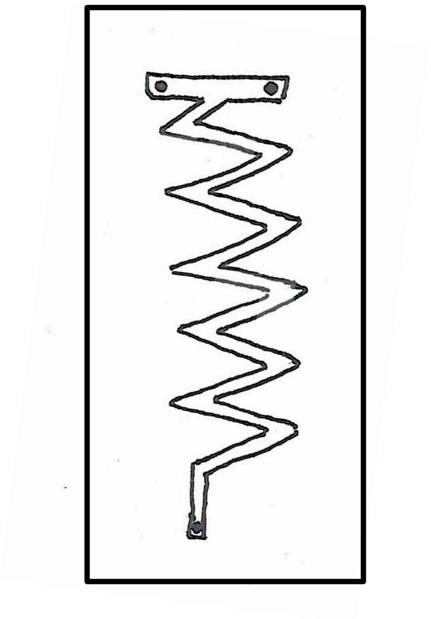

Connect this device to the same fluidic control setup as in Part I and make observations. Speculate why this device behaves differently than the device in part I.

Show a member of the teaching staff that your setup works.

_____TA check

Part III: Passive pumping (30 min)

Create the following simple device using the same method as in Parts I and II, except do not use the plasma wand for this device-pressures are low enough that reversible bonding (PDMS sticks to PDMS) will suffice. Also, larger hole punches are suggested for this design. (~ 2 mm)

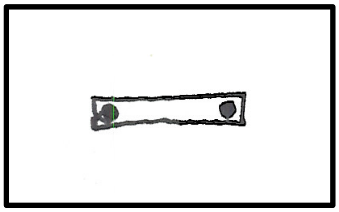

Fill the device by covering one port with a large droplet and aspirating with your squeeze bulb pipette on the other end.

Once the device is filled, place different sized droplets on either side and record what happens.

Have fun with colors and observe passive pumping.

Show a member of the teaching staff that your setup works.

_____TA check

If time permits, make additional devices of your own design and investigate this phenomenon.

Part IV: A microfluidic devices to control and observe euglena (15 min)

Here you will make the framework for a biotic game. First design a circuit with which you can create a variable intensity LED using a potentiometer. (Refer to your notes on LED circuits, ask for help if you get stuck)

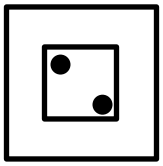

Next make the above device and load it with Euglena. Choose your favorite method for making and loading based on the two you have already tried.

After loading the device with Euglena, demonstrate that you can drive the organisms around with the light produced by your circuit, while viewing with the simple microscope you built in the optics unit.

TA check____

If time permits:

How does the organism response vary with light intensity? (Low, medium, high)

What fraction of the organisms respond?

Shoot videos of these responses.

<u>Part V: Integrated valves (30 min)</u>

See Nate for a demonstration of a micromixer with integrated valves TA check___

<u>Part VI: Microfluidics of wine (15 min)</u>

See Nate for an explanation of the 'tears of wine effect' TA check___

Summary Error analysis (0 min)

Not required for this lab.

Discussion (20 min)

Based on your activities in lab today, describe two ways that microscale fluid physics differs from macroscale fluid physics.

Describe one benefit and one disadvantage of rapid prototyping methods.

Report how long each section took. Give one thing you especially liked from this lab, and one change that would improve the material.
