# Supplementary material for: A Biotic Game Design Project for Integrated Life Science and Engineering Education
Source: PLoS Biol. 2015 Mar 25;13(3):e1002110. doi: 10.1371/journal.pbio.1002110 (PMC4373802; doi:10.1371/journal.pbio.1002110)
Supplement: S23 Text — This document provides requirements, notes, and starter modules on various components of the final biotic game project. (DOCX) [file pbio.1002110.s023.docx]

**Modules 13-20: Building a Biotic Gaming System**

## This final project has (at least) two objectives:

(A) This setup lets you integrate (nearly) all the techniques/topics that you would encounter when building an automated experimentation apparatus in the life sciences:

1. how to build a microscope / basic optics;
2. electronics;
3. I/O cards;
4. object tracking / image analysis;
5. rapid prototyping / CAD / 3D printing;
6. basic structural mechanics;
7. microfluidics / housing of biological material;
8. system integration;
9. documentation
10. creativity and teamwork.
11. and more….

(B) The setup is ultimately intended for educational use for children, students, or adults. The player should be able to steer the organism around and thereby experience and learn about these organisms.

**
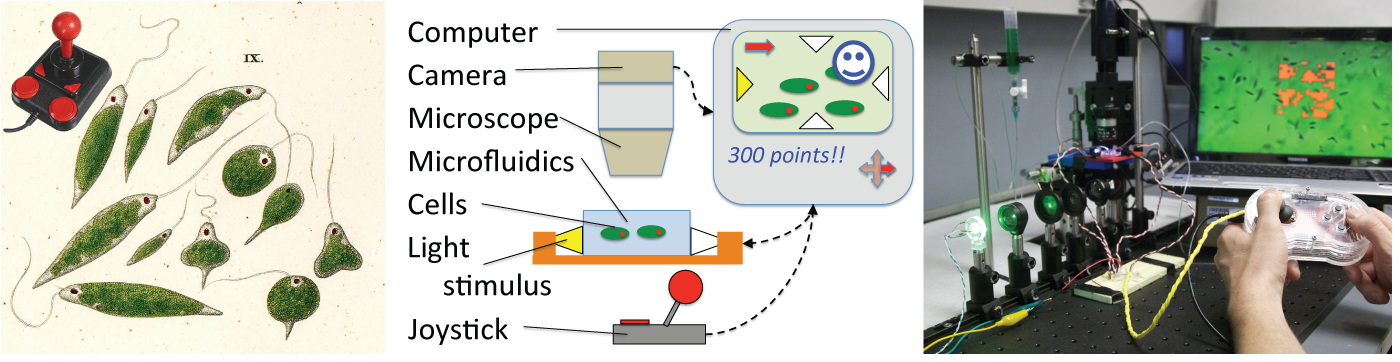
**

Fig. Schematic overview of project.

## Specific Task:

Build a biotic game setup that:

(1) houses microorganisms in a microfluidic chamber;

(2) enables a human player to influence the behavior of single celled micro-organisms (such as euglena via directional light stimuli);

(3) measures at least one state variables of the microorganism in real time (such as position) and displays them on a video screen via magnification by a microscope and webcam;

(4) integrates an Arduino into the setup that reads-in and outputs at least one variable more than once during the game;

(5) provides player with a game rules and goals;

(6) an individual game should not last longer than 60 sec; preparation time between games should not be longer than 60 sec;

(7) report and document your progress frequently on a project website,

(8) describe in 50-250 words what experience you aim the player to get (could be educational, emotional, etc.),

(9) at the end of the course give a 3 minute presentation to the class (in form of a 3 minute pre-recorded video) and a hands- on demonstration, and

(10) include on your website a final characterization, example video and discussion of your game device.

The following sections provided the milestones for the students, but students were encouraged to work ahead when ready or work out of order. During the first two years of teaching students built a microscope using Lego, in the third year we let them reuse the microscope they had developed during the optics session.

The tools and materials required to implement this project depend on intended learning outcomes, what is available, how much time there is in the course, how much one wants to prefabricate for the students, and how complex the final project is supposed to be. We refer to the materials in the preceding teaching materials on electronics, optics, and microfluidics. Generally one is assumed to have:

- Basic electronic equipment + Arduino + webcam
- Computer
- Programming language (such as MATLAB or Arduino environment)
- Basic microfluidics
- Optical components; lenses or objectives (an existing school microscope could be used as well)
- Basic fabrication and prototyping tools (such as drill, saw, 3D printer,…)
- Micro-organisms (see below)

**Special note on the used organisms:**

Many microorganisms are light responsive and could, in principal, be used. We found that *Euglena gracilis* (152800 Carolina Biological, NC, USA, #) was particularly suitable given its size (easy to visualize), robust response to light, long term viability (just leaving the organisms in the container as supplied by Carolina under ambient light for well over a month the organisms were still viable and responsive), already growing at a good density, i.e., they can be directly put into the chamber (potentially diluted) as they are in the container, and they are commonly used in school classrooms for phototactic experiments, i.e., they are safe to use and match current curriculum.

Various other microorgansism organisms can be added to the mixture such as Stentor, euglena, volvox etc., which can be obtained from supply companies like Carolina. The euglena culture was kept in the sealed original glass containers as supplied by Carolina or in the open reservoir of the setup at room temperature (~24ºC) and ambient room light (30-100 lux). Light cycle was determined by the building (usually only off between 2 a.m. and 6 a.m.). (Note: carolina recommends low light / 22ºC). Euglena can also be concentrated by centrifuging at 1000g for 10 minutes, but we did not find this to be necessary.

In previous years we let the students design games using paramecia under electrical stimulus (Riedel-Kruse, Chung, Dura, Hamilton, & Lee, 2011), but we found that euglena and light response are much easier to work with, furthermore the microfluidic chamber design is more straight forward.

## Specific sub-tasks

1. **Microscope**

Students build a microscope essentially making similar configurations to what they were doing during the optics session (see instructions there); choice of lenses and setup depends on student’s magnification desires and other project choices. The students should understand the basics of illumination, image formation, and magnification. One LED will illuminate the specimen and thus you must direct its light toward the specimen. Students can have a set up as simple as placing the LED under the specimen holder. To improve uniformity, they may opt to include a lens between the LED and the sample to make the illumination near collimated through the sample. A (thin) sheet of paper in the light path could act as a diffuser.

Microscopes can be built in various ways (such as with Lego as we did in the first year or with Thorlabs parts as in the last years).

Alternatively, instead of building a microscope, existing school microscopes that allow for camera attachment could be used (such as MI-5000 HD; Microscience tools). Choice of camera should also be considered as not all cameras interface well with the software used for tracking – using the webcam chip as described below (potentially with an adapter / mount to hold the chip at the correct position in the optical path if needed; such an adapter can be 3D printed, for example).

For scaled down versions, we found simple diy microscopes (ex: <http://www.instructables.com/id/10-Smartphone-to-digital-microscope-conversion/>) paired with a smartphone also work.


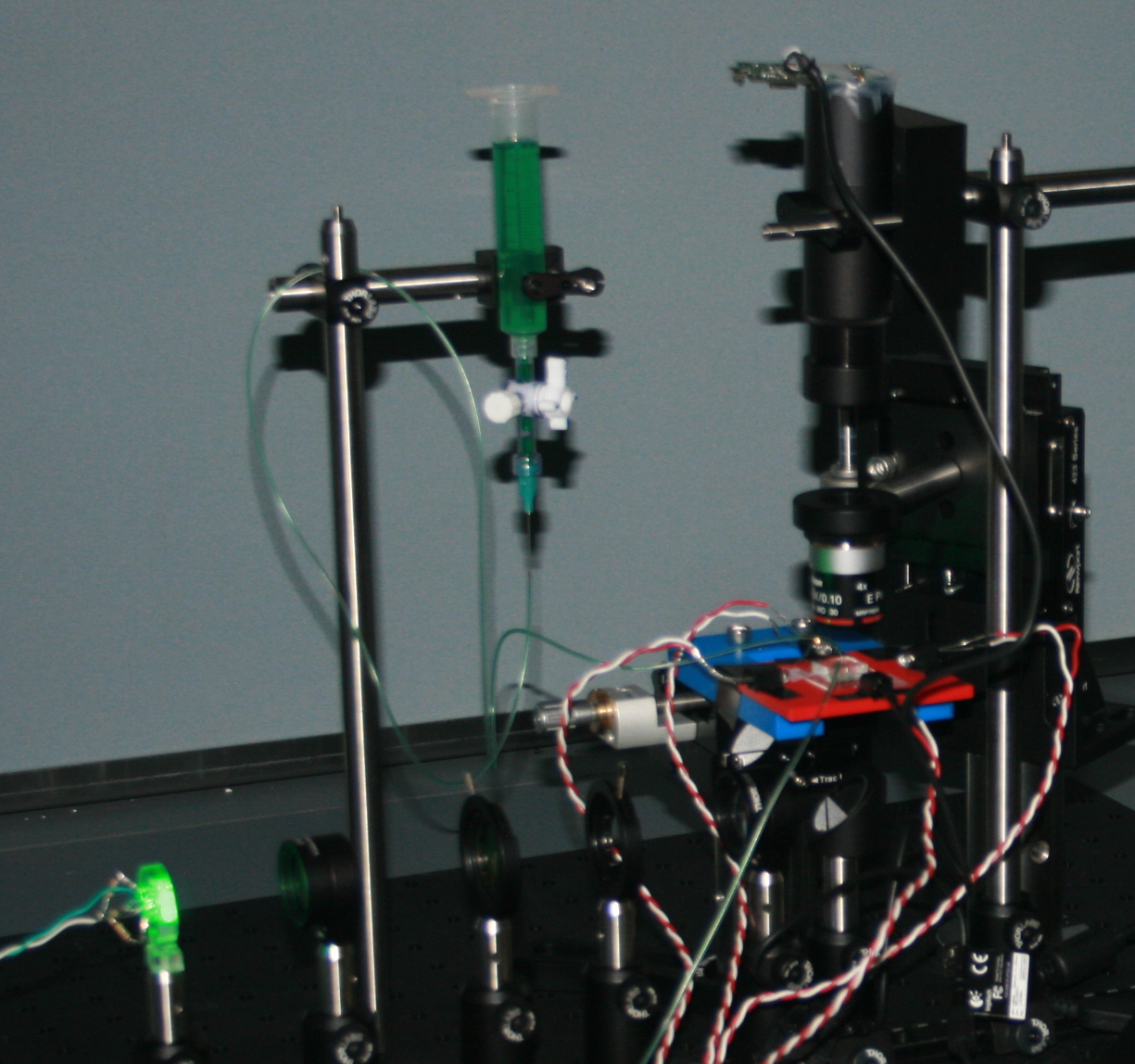

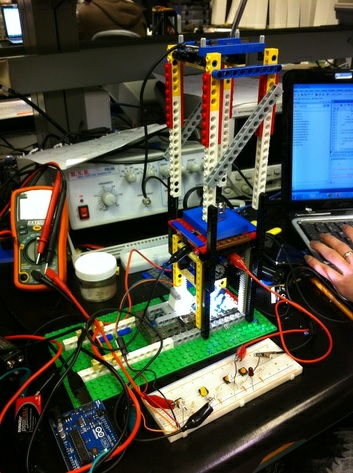


Fig.: Example of microscope setups with Thorlabs part (left) or Lego (right). Image credits: NJC

1. **Projection of Image on Exposed Webcam Chip:**For imaging a webcam is used where the lens is removed (see optics modules). The students should be able to remove the housing and lenses from the webcam to expose its CCD/CMOS chip, and place the naked webcam chip in the tube lens focal plane to sample the image of the magnified specimen. They should then find a way to attach the exposed webcam to their microscope in place of the eyepiece. At the end of the activity they should be able to see the image of the illuminated object on the webcam program of their computers. They should be able to observe cells through their microscopes. They should take pictures/videos to prove they have achieved the goal.
2. **Interfacing Arduino with MATLAB (or any other programming language):**

Students used an Arduino for the two-way communication between computer and hardware. The choice of programming language can significantly affect how easy it is to interface with the external world (Arduino as well as camera) and to implement real-time tracking algorithms. In our course we used MATLAB as one of the programming languages emphasized in college education, but many alternatives (especially open source) exist, such as the Arduino environment.

We used the different pin modes and I/O capabilities of the Arduino Uno. Students should understand the basics of programming the I/O pins of the Uno and downloading the code onto the card. They should be able to run the script created, which interfaces the Arduino card with MATLAB, and be able to program the digital and analog I/O pins. They should be able to see the results using an oscilloscope or multimeter.


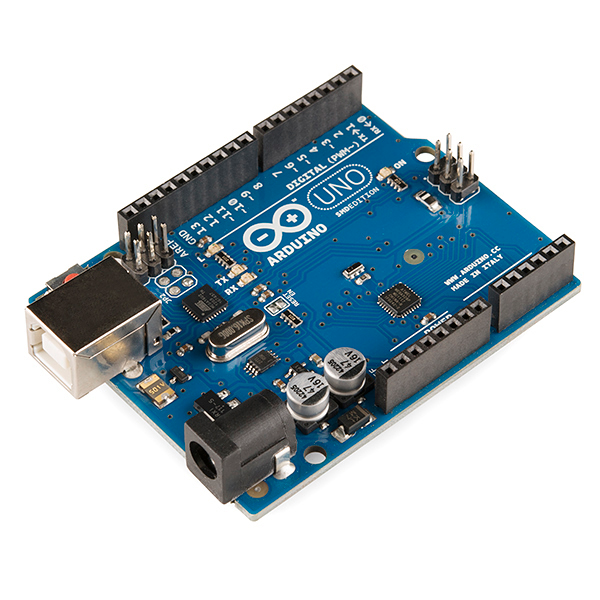


Fig.: Arduino board. Image from: http://commons.wikimedia.org/wiki/Category:Arduino_Uno#mediaviewer/File:ARDU-03.jpg

1. **Arduino based Joystick**

As a joystick the students can develop and test their own circuits on a bread-board that enables the control of 4 LEDs. Given the graded response of the organisms we found it advantageous to enable analog (rather than digital) control of the LEDs: If the light is too dim (compared to the background light) the euglena cells might not respond at all; if it is too bright, then the euglena cells perform local tumbling instead of directed motion. The usage of an analog control then enables the user to adjust with live feedback.

One simple solution (either after the students developed their own joystick on breadboard – or right away) is to use a pre-made joystick. Here the Arduino Joystick Shield Kit (DEV-09760) was very suitable – and the students can work on their soldering skills while assembling it.


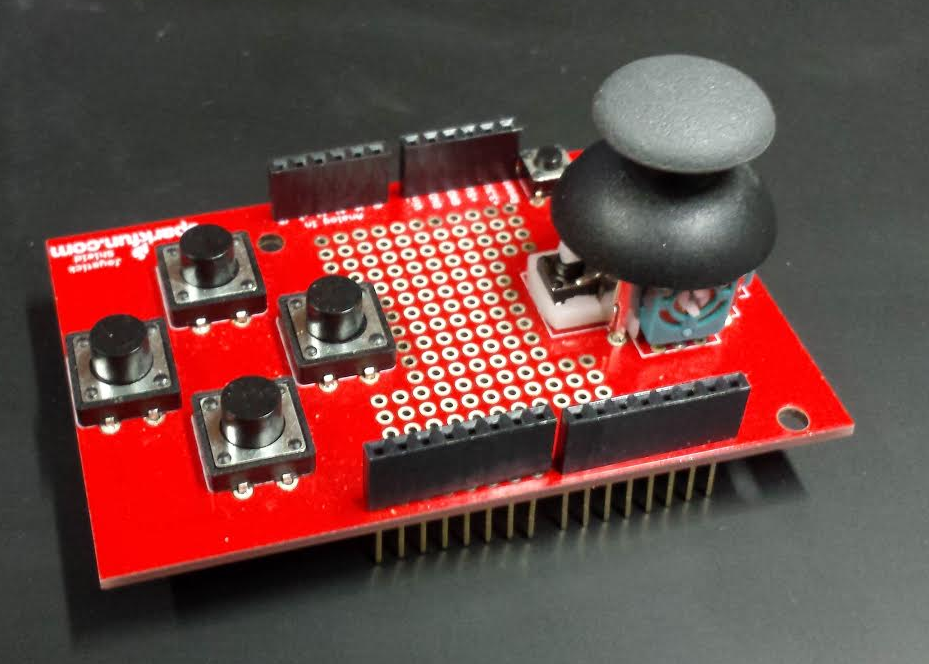


Fig.: Arduino Joystick Shield Kit (DEV-09760) Images credit: NJC

1. **Controlling LEDs using Arduino:**Students should understand the basics of how an LED works as well as what is required to power a high power LED. They should be able to interface with the LED through an I/O port of the Arduino card and be able to control the LED using a MATLAB script or command. They should understand the anode v/s cathode of the LED and be able to solder the wires from the LED to the driver circuit accordingly.
2. **Building Microfluidic Chamber with integrated long-term culturing and working with Euglena Cell**Students should be able to construct a microfluidic chamber by the silicone sheet method (see instruction material on microfluidics). The microfluidic chamber can be loaded with euglena but left unconnected to any tubing, which is fine for use on the scale of an hour, but would eventually dry out.


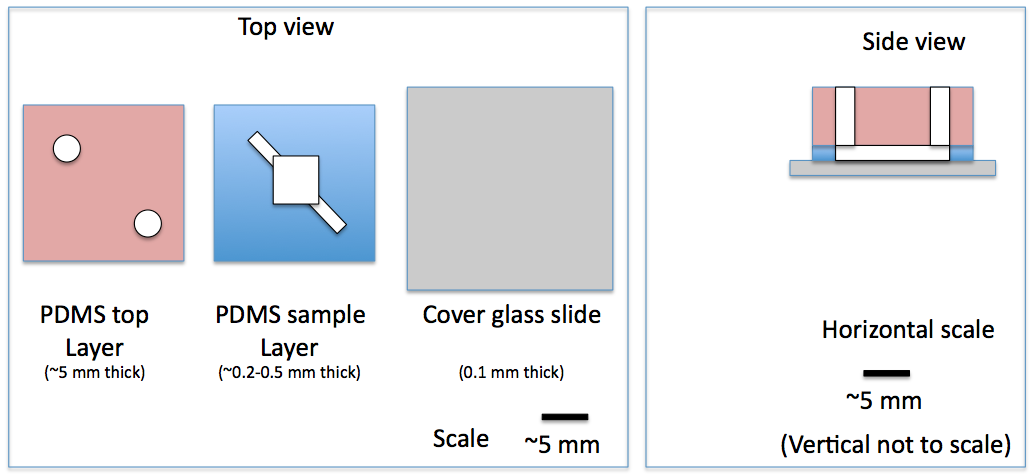

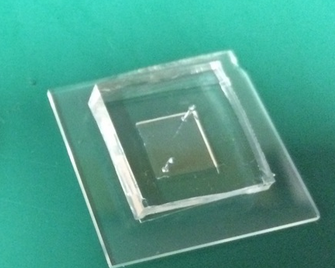


Fig.: Layout of microfluidic chip design.

Alternatively, the microfluidic chip can be connected to two reservoirs to keep euglena stable for days. The upper reservoir should be left open, while the lower ones should be left closed, so that any evaporation in the chamber will always be compensated for. Image credits: IRK

**
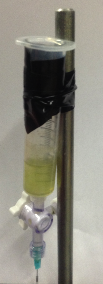

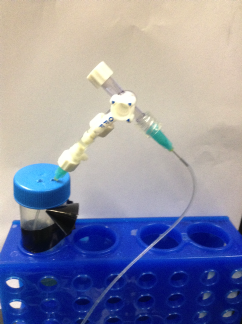
**

Fig.: Layout of connecting microfluidic chip to two gravity driven reservoirs - left: source (up); right: sink (down). Image credits: IRK

1. **Building Support Structure for Microfluidic Chamber and LED**Students design with Solidworks a structure that can hold the microfluidic chamber in place, and at the same time keep the LED at a level which guides light into the chamber for stimulating the Euglena. This design is then 3D printed. Another option for rapid prototyping is to fix the LED’s with clay on a flat plastic or cardboard support.


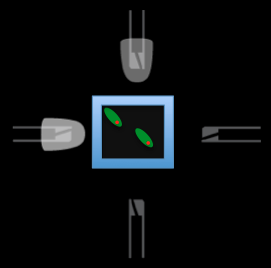

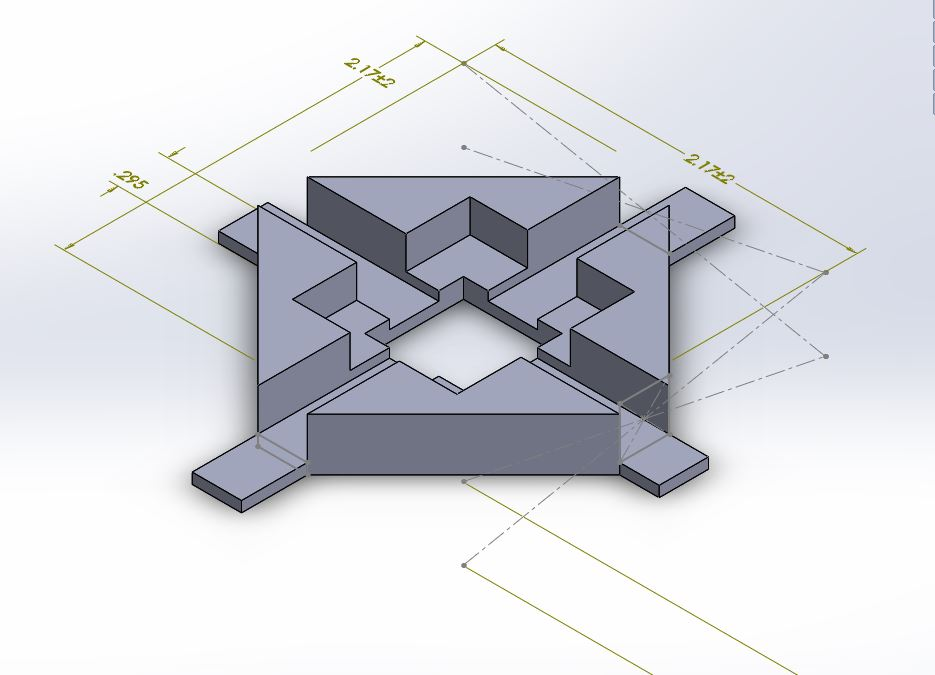

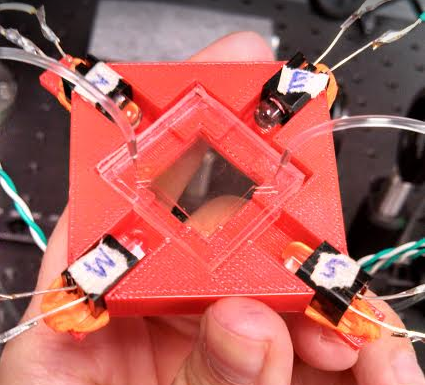

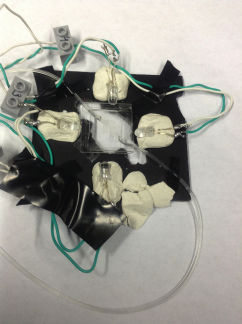


Fig.: Design of sample holder and LED actuation (from left two right): Schematic square chamber from top with 4 LED; Solidwork design; 3D printed piece assembled with LED and chamber; alternative rapid prototyping example using card-board with a hole and LED held by clay. Image credits: left to right, IRK, NJC, IRK, NJC

1. **Euglena phototaxis**At this point students should be able to stimulate the swimming motion of Euglena inside the chamber with the 4 LEDs and observe live on a computer screen via webcam.
2. **Controlling Euglena Movements using MATLAB Scripts:**

Students should be able to write short MATLAB scripts that control the direction of illumination and hence the direction in which the Euglena move via Arduino. The students should capture a video to prove that their euglena respond to the light stimulus in a directional manner.

1. **Live Webcam Image Capture and Object Tracking:**Students should write a MATLAB script with which they can capture live webcam video and process the images. They should be able to track any movements in the video and follow the object by drawing a box around it.

For developing this code, rather than directly work on tracking Euglena, students can first track a black dot made with a pen on a microscope slide and then move the slide around. This has the advantage that both number and motion of the object is controlled (while working with Euglena directly is not).

There are different methods for object tracking and different software packages provide many built-in functions. Many challenges (and trade-offs) exist, such as whether the algorithm is sufficiently fast to run in real time on the computer at hand, whether it is reliable enough, how complicated the algorithm is to be actually implement by the students, or how susceptible it is to imaging noise and fluctuations in illumination.

One can distinguish between tracking cells directly vs. instead setup “motion detectors” that provide feedback of whether for a specified area on the screen an object has moved in or out. We generally went with the later approach. The following pseudo-code with comments presents the main idea of computing a running average background image that is subtracted from the current image. A threshold is then applied and tested within any region of interest to determine whether the resulting intensity has sufficiently increased, which corresponds to a detected event:

LOOP in real time (10 times per second):

Average_image := 0.9*Averag_image + 0.1*Current_image

% Generates an average image that insensitive of any static or slow changing background

Object_image := Average_image - Current_image

% Generates an image that has information only about currently moving objects, i.e., is background corrected

Object_image := THRESHOLD [Object_image]

% Keeps only region in the image that are moving and of sufficient contrast

IF Object_image [Region_of_interest] > VALUE

% Determines whether in the specified region the intensity as sufficiently changed, which corresponds to a cell entering or leaving that area

OBJECT_DETECTED => EVENT

% initiates further events, such as scoring

END

END

This algorithm can be tuned and improved, but provides the main concept.

1. **Integrating Live Image Tracking and Euglena Control Scripts:**Students should be able to combine their scripts/functions so they can be called from a single MATLAB script. Through this single script, they should be able to take a live capture of the video feed coming from the webcam, track the movements in the euglena, be able to control the light stimulus in the chamber via Arduino. We included some starter codes below.
2. **Programming a simple game interface:**Once students have reached this stage where they can control the direction of the LED and hence the direction of euglena, and can track the euglena movements (or at least detect of whether the enter or leave regions of interest), they should use their imagination to create a game by rendering overlaid game images on the live camera feed.


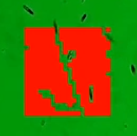

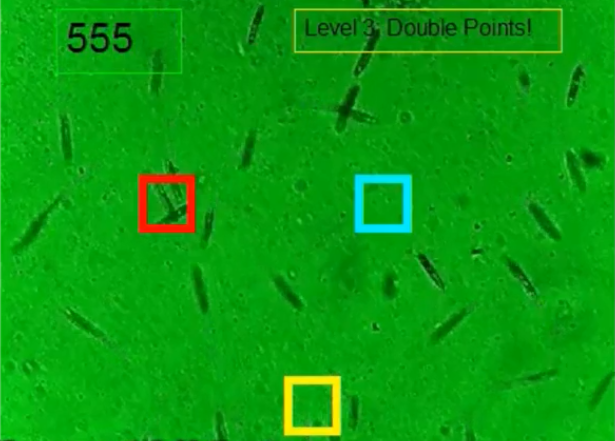

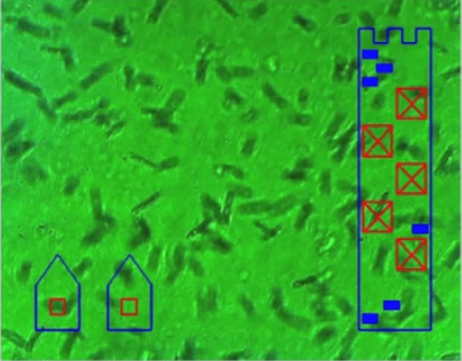


Fig.: Examples of games that can be programmed (see also supplementary movie). All games have the same underlying concept that certain areas of the screen detect of whether cell swim into them, which then triggers events. Image credits: NJC

Example on the left starts with an area of 20x20 boxes, where each box disappears when one cell has entered that area - the game score then is proportional to the number of boxes that disappeared during the game.

1. **Fabrication of game controller housing**

In order to facilitate some hands on practical skills like drilling, tapping holes, working with different materials we asked students to design and build a housing for their game controller. An alternative is to actually build all structural parts of the microscope – but this might have taken too long given the time we had available.


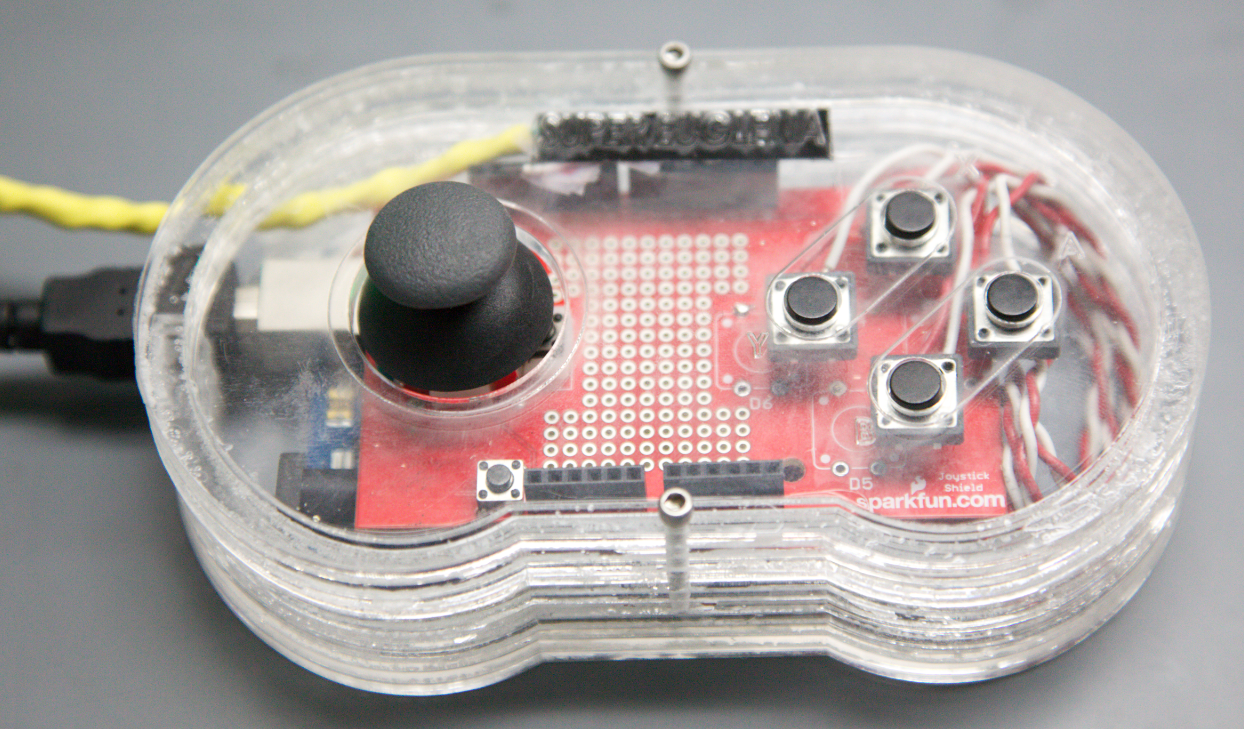


Fig.: Example of a game controller housing fabricated by the students. Image credits: NJC

1. **Optional: Game design**

We invited a game designer to the class who gave some perspectives and ideas. There are simple game design exercises for the students to go through (such as playing and modifying Tic-Tac-Toe – see (Zimmerman, 2014)). We also discussed with students simple strategies to setup a game – and what a game actually is in the first place (Salen & Zimmerman, 2004). Overall, given the complexity of the setup we kept this portion rather simple, and we emphasized to the students that the most basic way of implementing a game is to enable user actions (i.e., changing light, which in turn affect organisms responses), guide these actions towards a goal (such as hitting a virtual object on the screen), and then getting points.

1. **Optional: Ethical considerations**

Students were asked to discuss with each other the potential ethical considerations of playing with microorganisms – and they were also asked to read the corresponding paper: (Harvey, Havard, Magnus, Cho, & Riedel-Kruse, 2014) and also (Riedel-Kruse et al., 2011)). Students then wrote and submitted a short essay between 100 and 250 words why they would actively opt in or opt out to build a game. It was explicit that the course grade is not affected by either case. If they did not make a game (actually, no-one choose that option), they still needed to build an interactive device for a different purpose (such as research or art) that fulfills all the other criteria stated above.

1. **Project documentation on website and final presentation**

For documentation students used a lab notebook, furthermore each team created an online documentation on “weebly” (www.weebly.com). Students also recorded a 3 minute video (max) to be presented on the final day. After all video presentations we went back to the lab and everyone could play with everyone’s setup (one student of each team would walk around while the other would present the setup, and then switch).

1. **Optional: Public presentation**

Students were encouraged to demo their setups during a public outreach day

for high-school students.

**References**

Harvey, H., Havard, M., Magnus, D., Cho, M., & Riedel-Kruse, I. (2014). *Innocent fun or microslavery?: Public Responses to and Ethical Analysis of Biotic Games* (pp. 1–28). Hasting Center Report.

Riedel-Kruse, I. H., Chung, A. M., Dura, B., Hamilton, A. L., & Lee, B. C. (2011). Design, engineering and utility of biotic games. *Lab on a Chip*, *11*(1), 14–22. doi:10.1039/c0lc00399a

Salen, K., & Zimmerman, E. (2004). *Rules of play*. The MIT Press.

Zimmerman, E. (2014). How I Teach Game Design.Lesson 1: The Game Design Process. *Games and Culture*, 1–3.

**Appendix: MATLAB code examples to get started**

**Reading “key press”**

You can even use your computer keyboard direction keys for this. Through MATLAB, you can capture the “event”, when a keyboard key is pressed by the user and by using the “KeyPressFcn” function, even determine which key was pressed. Depending on which key is pressed, you can have the Arduino PWM outputs control the 4 electrodes accordingly.
To capture the key events, a figure window must be opened in MATLAB. While the figure window is open and a key is pressed, MATLAB’s “KeyPressFcn” function automatically jumps to the event processing function that you define in your code.
Here is how your code would look like:

function RegularFunction
 …
 figure('KeyPressFcn',@JumptoFunction); %When key pressed, jump
 …


function JumptoFunction(src,event)
    % Callback to parse keypress event data to print a figure
        if strcmp(event.Key,'leftarrow')
            TurnLeft()
            disp('Turning Left')
 …

**Image Tracking Pointers**

Live image tracking is not trivial. It take a lot of resources of the computer/MATLAB to display live video, capture video frames, and process frames without dropping the subsequent frames. Make sure your code is lean and efficient to ensure good performance from MATLAB/computer.

1. To display the live video captured by the webcam, use the command:

   vid = videoinput('winvideo',1);
2. There are many ways to display and capture this video which is being stored in the object “vid”. However, different methods use different amount of resources of the computer.
   To preview the image efficiently, use the following commands:

   vid = videoinput('winvideo',1);
   %KeyPressFcn to capture key presses while window open
   figure('KeyPressFcn',@JumptoFunction,'Name', 'My Custom Preview Window');
       uicontrol('String', 'Close', 'Callback', 'close(gcf)');   %creates close button on window

       %Create an image object for previewing without making computer slow.
       vidRes = get(vid, 'VideoResolution');
       nBands = get(vid, 'NumberOfBands');
       hImage = image( zeros(vidRes(2), vidRes(1), nBands) );
       preview(vid,hImage);
3. To capture the present image from the video use the command:

   image = getsnapshot(vid);
4. First take a few seconds average intensity of small target portions of the image. This will be the original intensity of those regions.
5. Continually get the new intensity of small portions of the image as objects move in front of the camera. When the object moves in front of the target area, the intensity changes. Compare when the intensity changes to the original intensity in that region.
6. Make 2 adjacent and adjoining target areas to figure out the direction of the movement of object.
7. You can display rectangles around your target areas by using the rectangle command. For E.g.:

   rectangle('Position',[x y SizeCols SizeRows],'LineWidth',2,'EdgeColor','b');

   % This creates a blue rectangle starting at coordinates x,y and of size SizeCols x SizeRows pixels.
8. Make sure you clear your unused matrices – else it will continually use more and more resources and it will be hard to do live image tracking. Especially true when making loops – clear your matrices before rewriting on top of them.
9. Take absolute values of intensity changes instead of pure difference.
10. Make sure you set thresholds of the intensity change carefully. This will require several testing iterations. Also make sure the threshold is a function of the current image intensity, instead of an independent fixed threshold.
11. Before ending your program you must close the video capture by the webcam, else you will not be able to re-execute the code without getting an error msg from MATLAB saying your camera is still in use. To avoid this use the following commands before ending your program:
     
       stop(vid); % stops video capture
        delete(vid); % deletes object
        clear vid; % clears any stored frames from memory
        close(gcf); % closes the figure window
12. In case the program hangs or ends abruptly without execution of the above commands, when you re-execute your code, you are likely to be given an error that camera is still in use. To avoid that use the command “imaqreset;” in the beginning of your code. This automatically closes any instances of the video before re-calling that function.

**Video tester for Matlab**

%%******!!!!!! check to see if save file at end of code is active!!!!!!

close all;

clear;

imtool close all;

clc;

imaqreset;

% info = imaqhwinfo('winvideo');

% info.DeviceInfo.SupportedFormats;

%initialize video

vid = videoinput('winvideo',1, 'RGB24_640x480');

vidRES = get(vid,'VideoResolution');

nBands = get(vid, 'NumberOfBands');

hImage = image(zeros(vidRES(2), vidRES(1), nBands));

figure(1);

hold on;

preview(vid,hImage);

% stop(vid);

% delete(vid);

% clear vid;

**Example Game Run (Matlab code)**

**The following program contains the major programming blocks to get a game run in Matlab: grabbing images from the camera, interfacing with the arduino,**

%%clear up any old variables/objects

close all;

clear;

imtool close all;

clc;

imaqreset;

% info = imaqhwinfo('winvideo');

% info.DeviceInfo.SupportedFormats;

a = arduino('COM9');

%Pin IO Methods: pinMode digitalRead digitalWrite analogRead analogWrite

%set up IO pins

%

%drive left

a.pinMode(4,'input');

a.pinMode(7,'output');

a.digitalWrite(7,0);

%drive right

a.pinMode(8,'input');

a.pinMode(12,'output');

a.digitalWrite(12,0);

%run stop switch

a.pinMode(2,'input');

%initialize video

vid = videoinput('winvideo',1, 'RGB24_640x480');

vidRES = get(vid,'VideoResolution');

nBands = get(vid, 'NumberOfBands');

hImage = image(zeros(vidRES(2), vidRES(1), nBands));

figure(1);

hold on;

preview(vid,hImage);

%initialize variables

tcurrent = clock;

tlastimage = tcurrent;

tlastgoal = tcurrent;

tlastsample = tcurrent;

tstart = tcurrent;

RUN = 1;

Left = 0;

Right = 0;

goalon = 0;

n = 0;

samplecount = 0;

Llaststate = 0;

Rlaststate = 0;

while RUN == 1;

%-----------------------------------------------

%%events

image = getsnapshot(vid);

%check time variables

tcurrent = clock;

%time since start of game

deltTgame = tcurrent(6) - tstart(6);

if deltTgame < 0;

deltTgame = deltTgame + 60;

end;

%time since last goal display

deltTG = tcurrent(6) - tlastgoal(6);

if deltTG < 0;

deltTG = deltTG + 60;

end;

%time since last sample

deltTS = tcurrent(6) - tlastsample(6);

if deltTS < 0;

deltTS = deltTG + 60;

end;

%check arduino inputs

if deltTS > 0.1;

%check RUN switch

RUN = a.digitalRead(2);

%check Left switch

Left = a.digitalRead(4);

%check Right switch

Right = a.digitalRead(8);

end

%-----------------------------------------------------------

%%services

%end game after 30 seconds

% if deltTgame > 30;

% RUN = 0;

% end

%control light direction (steer cell)

if Left > 0 && Llaststate <1;

a.digitalWrite(7,1);

Llaststate = 1

elseif Left < 1 && Llaststate > 0;

a.digitalWrite(7,0);

Llaststate = 0

else

end

if Right >0 && Rlaststate <1;

a.digitalWrite(12,1);

Rlaststate = 1

elseif Right <1 && Rlaststate > 0;

a.digitalWrite(12,0);

Rlaststate = 0

else

end

%toggle goal draw

if deltTG > 5;

tlastgoal = tcurrent; %reset time for goal display

if goalon==0;

goal = rectangle('Position',[220,4,200,472],'LineWidth',2,'EdgeColor',[255/255,0/255,0/255]);

goalon =1;

elseif goalon ==1;

delete(goal);

goalon = 0;

else

end

end;

%sample image area for cell

if deltTS > 0.1;

tlastsample = tcurrent; %reset time for sample

if goalon ==1;

image = image(220:419,4:475,:);%sample the goal area

goalcurrent = mean(image,3); %convert to gray from RGB

%goalcurrent =image(:,:,2); %convert to gray from green LED

if samplecount == 0;

goallast = goalcurrent;

samplecount = 1;

else

imageDiff = abs(goalcurrent - goallast);

[rows,cols,vals] = find(imageDiff>50);

diffpixels(samplecount) = length(vals);

goallast = goalcurrent;

samplecount = samplecount + 1;

end

else

end

end

end;

%nice to see on screen if out of while loop

finished = 1

figure(2);

plot(diffpixels);

score = sum(diffpixels)

a.delete;

stop(vid);

delete(vid);

clear vid;
