# Supplementary material for: A Biotic Game Design Project for Integrated Life Science and Engineering Education
Source: PLoS Biol. 2015 Mar 25;13(3):e1002110. doi: 10.1371/journal.pbio.1002110 (PMC4373802; doi:10.1371/journal.pbio.1002110)
Supplement: S25 Text — This document goes over the time, resources, and instructions needed to run a short version of the biotic game project. (DOCX) [file pbio.1002110.s025.docx]

Alternative biotic game project implementation

Overview:

We tested a low-cost, reduced time, version of the biotic game project for younger students. We implemented this during Stanford SPLASH, a weekend when Stanford affiliates can volunteer to teach classes to high school and middle school students. To do this, we simplified and streamlined the assembly of the hardware, electronics, and software. We also had students from our undergraduate course present their biotic game projects. Below we include a framework for this activity for others to use as a guide. We taught 2 sessions, each with around 8 students.

Learning goals:

- Get students assembling/building devices

- Show the types of skills used in creating life science devices

- Introduce the concept of biotic games

- Allow students to see the microscopic world

- Inspire students about science

Age range:

Advanced middle school to high school

Time frame:

3 hours for teaching the unit

+prep time (variable depending on instructor background and resources)

Outline:

*Introduction*

5 min overview of what the students will be doing, and brief explanations of how the various parts of the setup function.

*Microscope*

Students assemble the microscopes

30 min

*Samples*

Students collect and look at samples using their microscopes (plants, insects, and fibers are especially interesting)

30 min

*Electronics and carrier*

Students assemble parts to create a microcontroller circuit which controls the LEDs and attach this to the LED holder

30 min

*Fluidics and cells*

Students assemble fluidic components, and load the *Euglena*

30 min

*Assembly*

Students place all components together to complete the assembly (as shown in Figure 1 below)

15 min

*Use/Games*

Students use their now completed devices to steer the swimming direction of *Euglena* using the phototaxis response

Students observe and play other more advanced biotic games we had in the lab (half of our undergraduate teams volunteered to come in and show their games)

30 min

*Conclusion*

10 min discussion on what was learned, time to address questions

Tips:

- try the activity once to make sure you know what you are doing

- ask students to bring a smartphone if they have access to one

- take pictures and videos on the smartphones

- students appreciated taking the microscopes home (around $5 cost)

- have example setups that the students can replicate

Details:

*Optics and imaging:*

We had students build a simple smartphone microscope.

We followed instructions from:

Instructions: http://www.instructables.com/id/10-Smartphone-to-digital-microscope-conversion/

Parts:

plexiglass stage (3, 7x7 inches)

carriage bolts (3, 5 inches)

nuts (9)

wingnuts (2)

washers (5)

plastic lens(1, ex: Aixiz, Product number: [AIX-LENS-123])

light (1, optional)

smartphone with camera (1, ideally students bring this with)


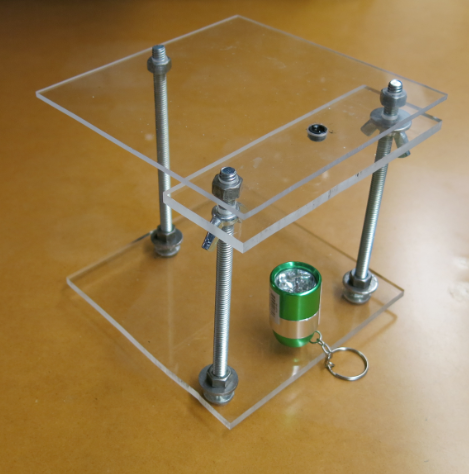
Figure 1. The assembled optical system. Note that a smartphone rests on top, with camera positioned over the lens. Image credits: NJC

*Electronics:*

We assembled a simple circuit to control LED intensity with a joystick.

Depending on available time, learning goals, and background, you may preprogram the Arduino for the students and solder the shield together (we did both of these this ahead of time)

Instructions: (refer to module 5 for circuits using LEDs, and basic programming of Arduino. Refer to the final project module section 4 and 5 for connecting the joystick)

Parts:

Arduino Joystick Shield Kit (1, Sparkfun: DEV-­09760)

white LEDs (4)

resistors (4)

breadboard (optional, depending on components chosen)

connecting wires (multiple)

Arduino Uno and USB cable (1)


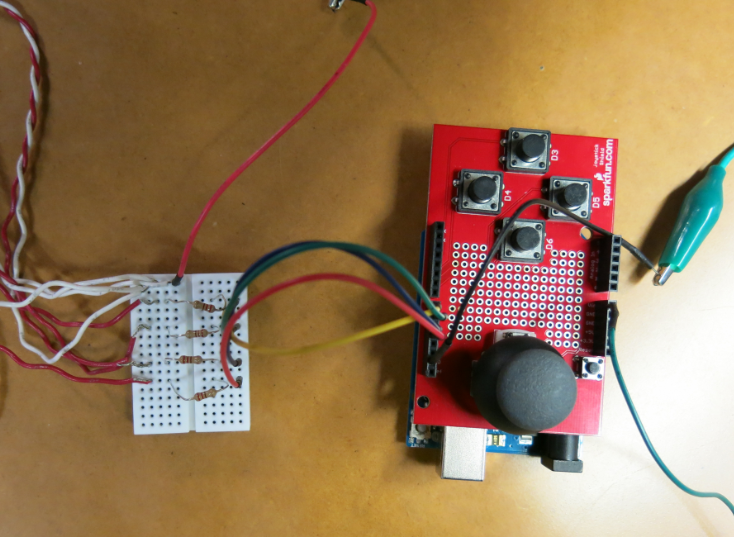
Figure 2. The circuit used to control the LED intensity with the Arduino mounted joystick shield. Image credits: NJC

*Fluidics:*

In this section students assemble the fluidics portion of the project.

Instructions: see point 6 on the final project module, and the entire microfluidics module

Parts:

Syringe (1, Becton Dickinson)

steel pins (2, 23 gauge Instech)

binder clip (1, to stop flow by pinching tube at outlet)

blunt needle (1, 23 gauge, Becton Dickinson)

tubing (2 feet, tygon: AAQ04103)

microfluidic chip (1, see * below)

tube of *Euglena* (1, Carolina)

*Microfluidic chip - here we premade these for students using standard softlithography techniques. For those without access to a clean room, following the instructions in the fluidics unit will result in equivalent chips. A functional device is an optically transparent flow cell with one inlet and one outlet, a height of around 100 um and a length and width of at least one mm.

*Fluidic chamber and LED holder:*

This holder secures the LEDs and the fluidic chip. Though we 3D printed the parts, a simple piece of cardstock would also work.

Instructions: see point 8 on the final project module

Parts:

3D printed holder (1)


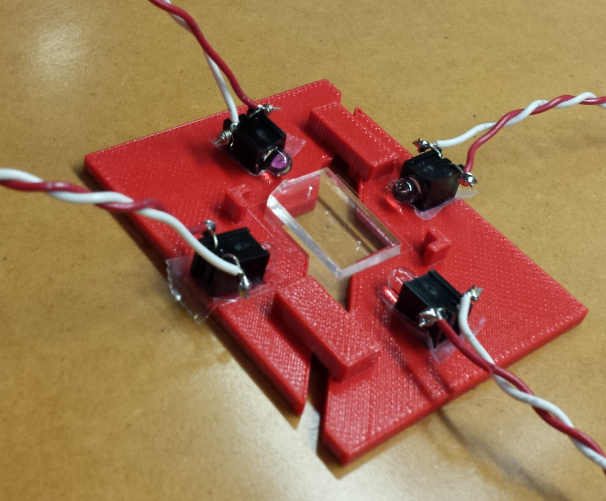
Figure 3. The fluidic chip and LED holder. The fluidic chip is made of clear PDMS in the center and holds the organisms. Image credits: NJC


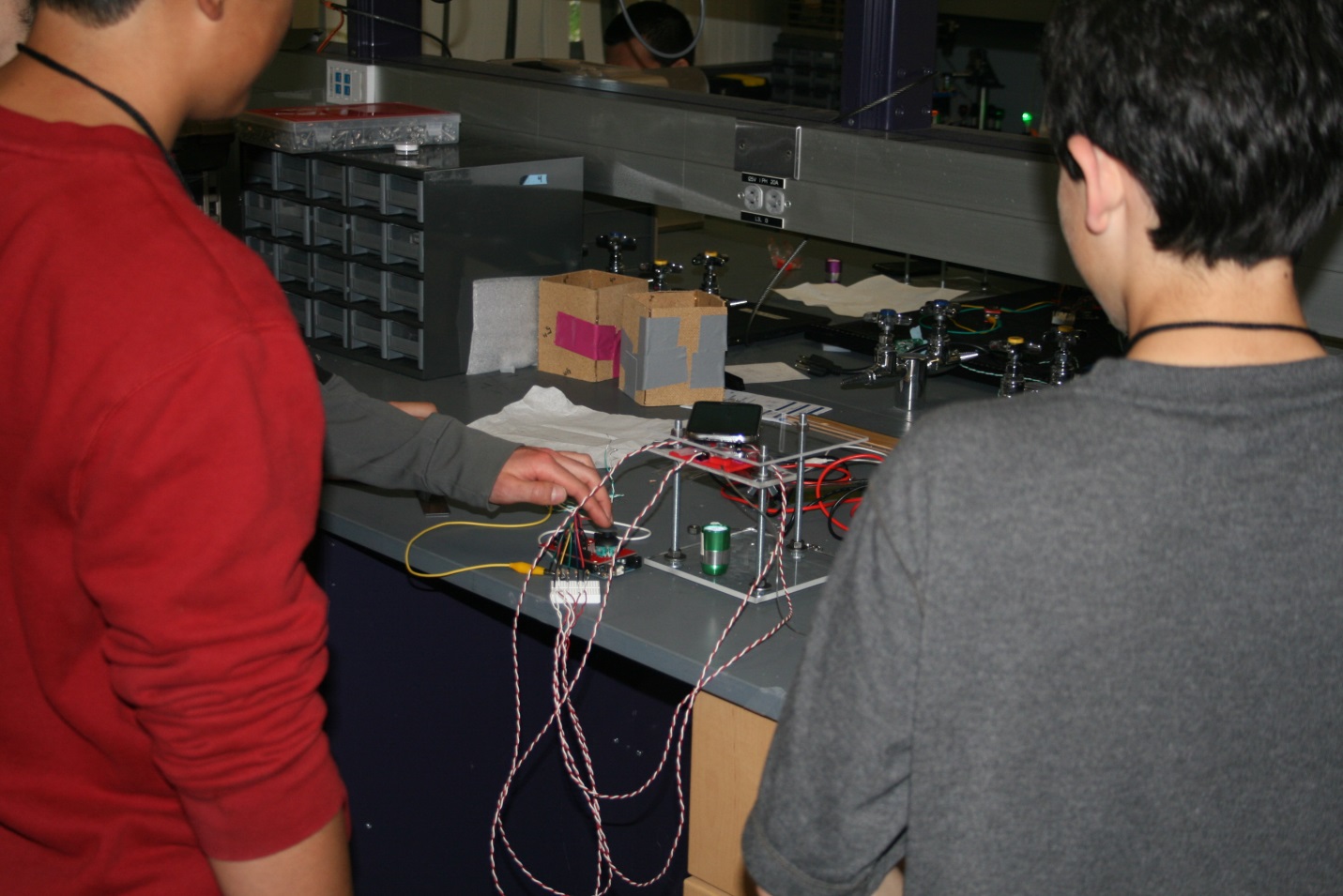


Figure 4. Students using assembled biotic game setup. Image credits: NJC
